# Supplementary figures and images for: Glucose hypometabolism prompts RAN translation and exacerbates C9orf72-related ALS/FTD phenotypes (part 2 of 2)
Source: EMBO Rep. 2024 Apr 29;25(5):21. doi: 10.1038/s44319-024-00140-7 (PMC11094177; doi:10.1038/s44319-024-00140-7)

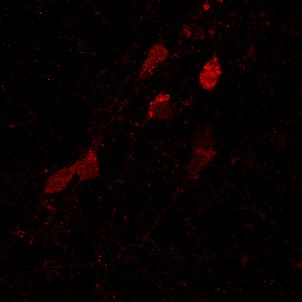

Supplement: Supplementary file 9 — Figure EVs Source Data [file 44319_2024_140_MOESM9_ESM.zip › SD EV figures/Supplementary Figure 4 - EV4/Panel S4C/representative images/JPGs/C3-Control(G3)_i3Ns_03_26_21_No_Glc_MAP2_ATF4_512_2_5-MaxIP-1.jpg]

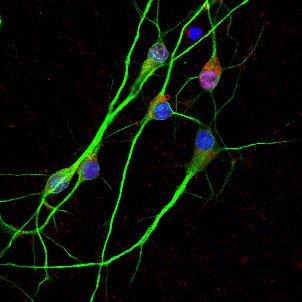

Supplement: Supplementary file 9 — Figure EVs Source Data [file 44319_2024_140_MOESM9_ESM.zip › SD EV figures/Supplementary Figure 4 - EV4/Panel S4C/representative images/JPGs/MERGE-Control(G3)_i3Ns_03_26_21_No_Glc_MAP2_ATF4_512_2_5-MaxIP-1.jpg]

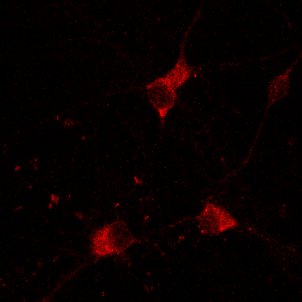

Supplement: Supplementary file 9 — Figure EVs Source Data [file 44319_2024_140_MOESM9_ESM.zip › SD EV figures/Supplementary Figure 4 - EV4/Panel S4C/representative images/JPGs/C3-Control(G3)_i3Ns_01_26_21_Complete_MAP2_ATF4_512_1_4-MaxIP-1.jpg]

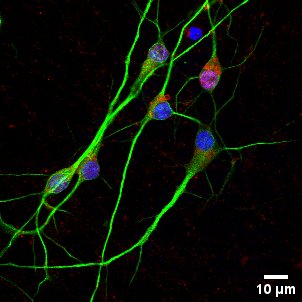

Supplement: Supplementary file 9 — Figure EVs Source Data [file 44319_2024_140_MOESM9_ESM.zip › SD EV figures/Supplementary Figure 4 - EV4/Panel S4C/representative images/JPGs/sb-Control(G3)_i3Ns_03_26_21_No_Glc_MAP2_ATF4_512_2_5-MaxIP-1.jpg]

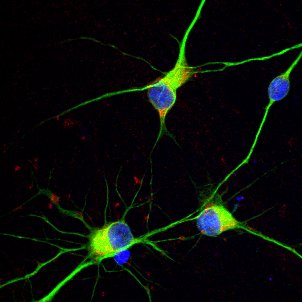

Supplement: Supplementary file 9 — Figure EVs Source Data [file 44319_2024_140_MOESM9_ESM.zip › SD EV figures/Supplementary Figure 4 - EV4/Panel S4C/representative images/JPGs/MERGE-Control(G3)_i3Ns_01_26_21_Complete_MAP2_ATF4_512_1_4-MaxIP-1.jpg]

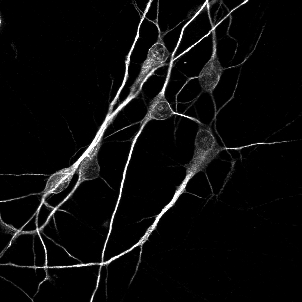

Supplement: Supplementary file 9 — Figure EVs Source Data [file 44319_2024_140_MOESM9_ESM.zip › SD EV figures/Supplementary Figure 4 - EV4/Panel S4C/representative images/TIFFs/C2-Control(G3)_i3Ns_03_26_21_No_Glc_MAP2_ATF4_512_2_5-MaxIP-1.tif]

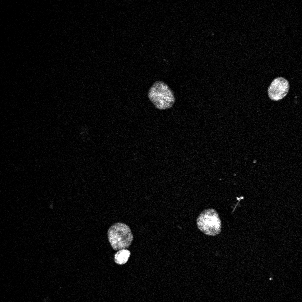

Supplement: Supplementary file 9 — Figure EVs Source Data [file 44319_2024_140_MOESM9_ESM.zip › SD EV figures/Supplementary Figure 4 - EV4/Panel S4C/representative images/TIFFs/C1-Control(G3)_i3Ns_01_26_21_Complete_MAP2_ATF4_512_1_4-MaxIP-1.tif]

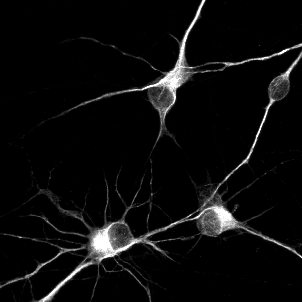

Supplement: Supplementary file 9 — Figure EVs Source Data [file 44319_2024_140_MOESM9_ESM.zip › SD EV figures/Supplementary Figure 4 - EV4/Panel S4C/representative images/TIFFs/C2-Control(G3)_i3Ns_01_26_21_Complete_MAP2_ATF4_512_1_4-MaxIP-1.tif]

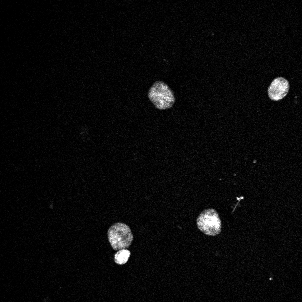

Supplement: Supplementary file 9 — Figure EVs Source Data [file 44319_2024_140_MOESM9_ESM.zip › SD EV figures/Supplementary Figure 4 - EV4/Panel S4C/representative images/TIFFs/MERGE-Control(G3)_i3Ns_01_26_21_Complete_MAP2_ATF4_512_1_4-MaxIP-1.tif]

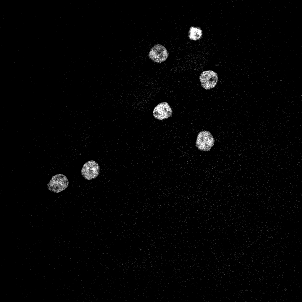

Supplement: Supplementary file 9 — Figure EVs Source Data [file 44319_2024_140_MOESM9_ESM.zip › SD EV figures/Supplementary Figure 4 - EV4/Panel S4C/representative images/TIFFs/MERGE-Control(G3)_i3Ns_03_26_21_No_Glc_MAP2_ATF4_512_2_5-MaxIP-1.tif]

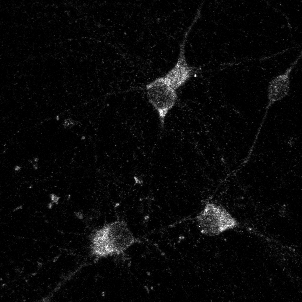

Supplement: Supplementary file 9 — Figure EVs Source Data [file 44319_2024_140_MOESM9_ESM.zip › SD EV figures/Supplementary Figure 4 - EV4/Panel S4C/representative images/TIFFs/C3-Control(G3)_i3Ns_01_26_21_Complete_MAP2_ATF4_512_1_4-MaxIP-1.tif]

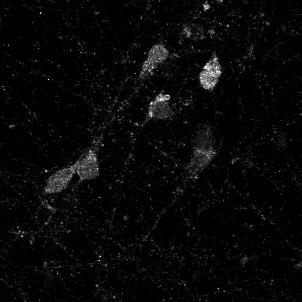

Supplement: Supplementary file 9 — Figure EVs Source Data [file 44319_2024_140_MOESM9_ESM.zip › SD EV figures/Supplementary Figure 4 - EV4/Panel S4C/representative images/TIFFs/C3-Control(G3)_i3Ns_03_26_21_No_Glc_MAP2_ATF4_512_2_5-MaxIP-1.tif]

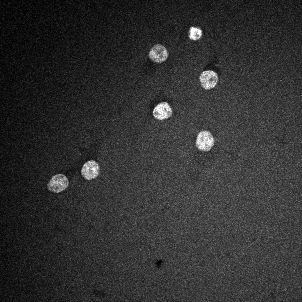

Supplement: Supplementary file 9 — Figure EVs Source Data [file 44319_2024_140_MOESM9_ESM.zip › SD EV figures/Supplementary Figure 4 - EV4/Panel S4C/representative images/TIFFs/Control(G3)_i3Ns_03_26_21_No_Glc_MAP2_ATF4_512_2_5-MaxIP-1.tif]

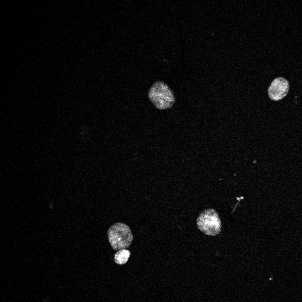

Supplement: Supplementary file 9 — Figure EVs Source Data [file 44319_2024_140_MOESM9_ESM.zip › SD EV figures/Supplementary Figure 4 - EV4/Panel S4C/representative images/TIFFs/Control(G3)_i3Ns_01_26_21_Complete_MAP2_ATF4_512_1_4-MaxIP-1.tif]

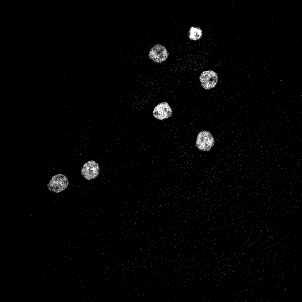

Supplement: Supplementary file 9 — Figure EVs Source Data [file 44319_2024_140_MOESM9_ESM.zip › SD EV figures/Supplementary Figure 4 - EV4/Panel S4C/representative images/TIFFs/C1-Control(G3)_i3Ns_03_26_21_No_Glc_MAP2_ATF4_512_2_5-MaxIP-1.tif]

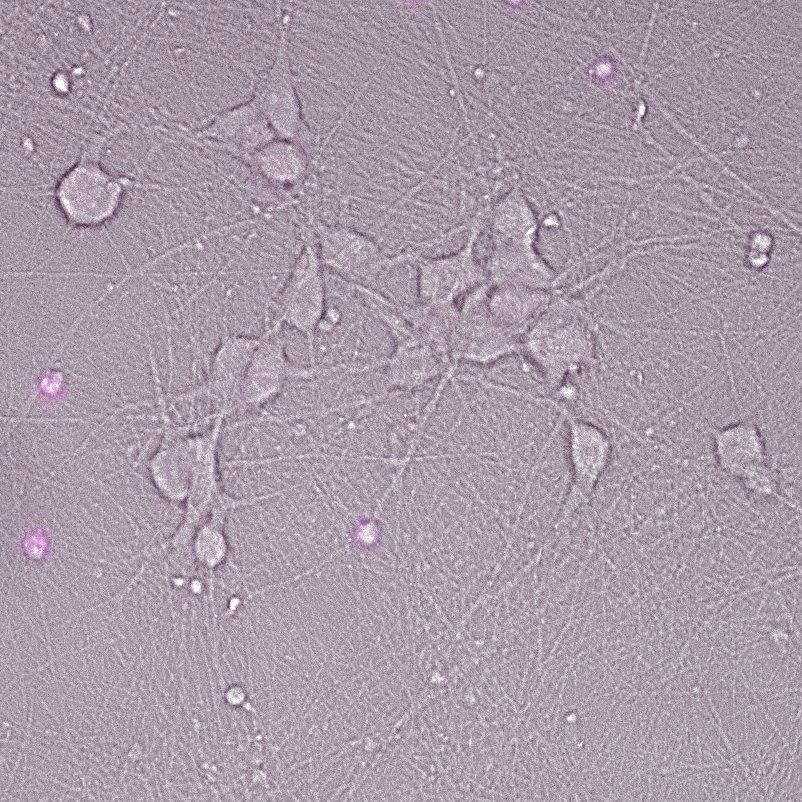

Supplement: Supplementary file 9 — Figure EVs Source Data [file 44319_2024_140_MOESM9_ESM.zip › SD EV figures/Supplementary Figure 3 - EV3/Panel S3B/representative images/cropped/G3_DMSO_48h-1.jpg]

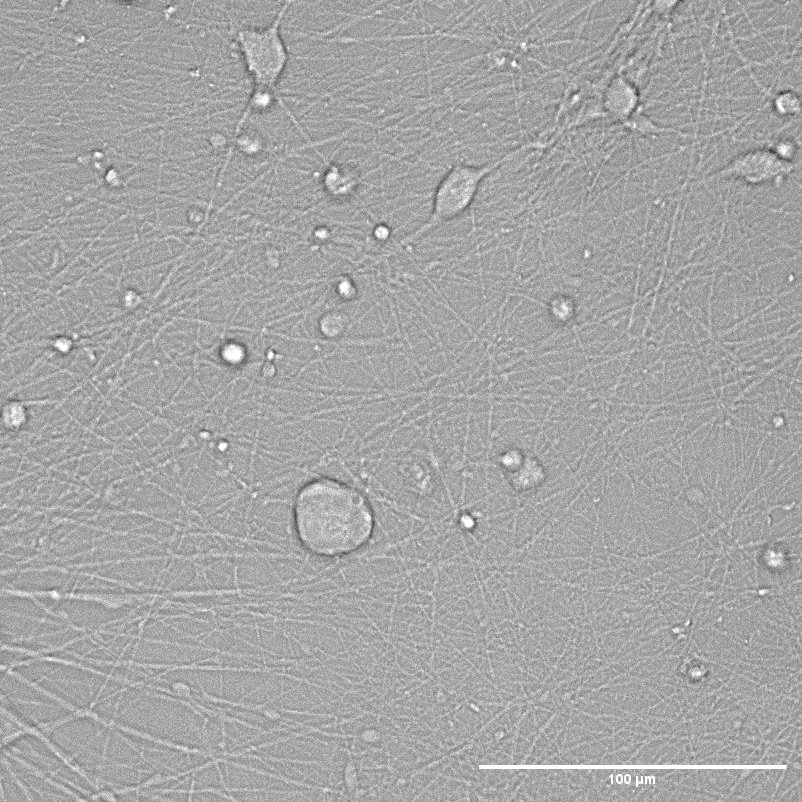

Supplement: Supplementary file 9 — Figure EVs Source Data [file 44319_2024_140_MOESM9_ESM.zip › SD EV figures/Supplementary Figure 3 - EV3/Panel S3B/representative images/cropped/scale bar-1-1.jpg]

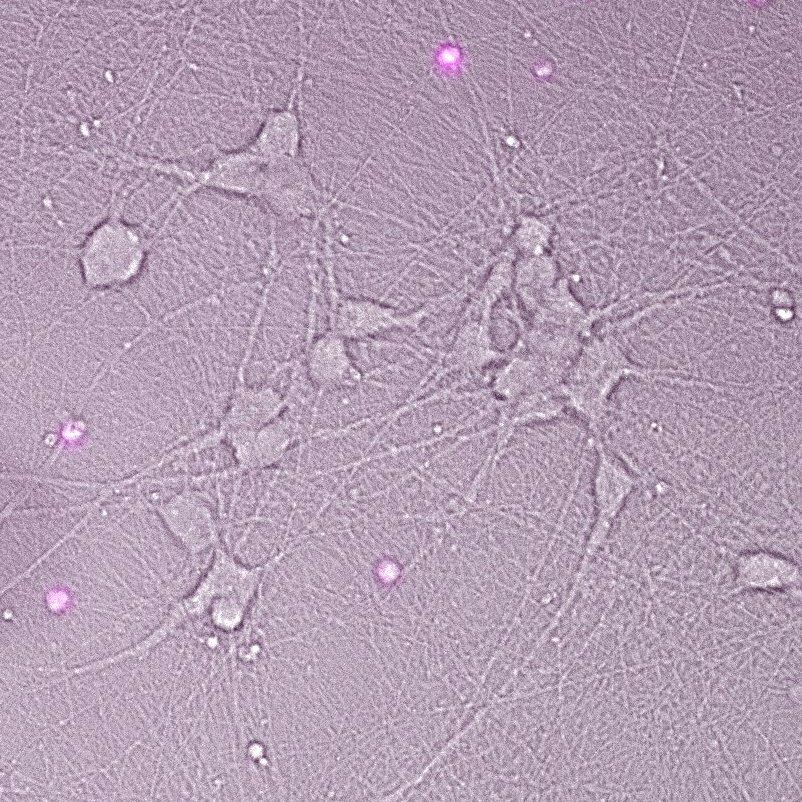

Supplement: Supplementary file 9 — Figure EVs Source Data [file 44319_2024_140_MOESM9_ESM.zip › SD EV figures/Supplementary Figure 3 - EV3/Panel S3B/representative images/cropped/G3_DMSO_0h-1.jpg]

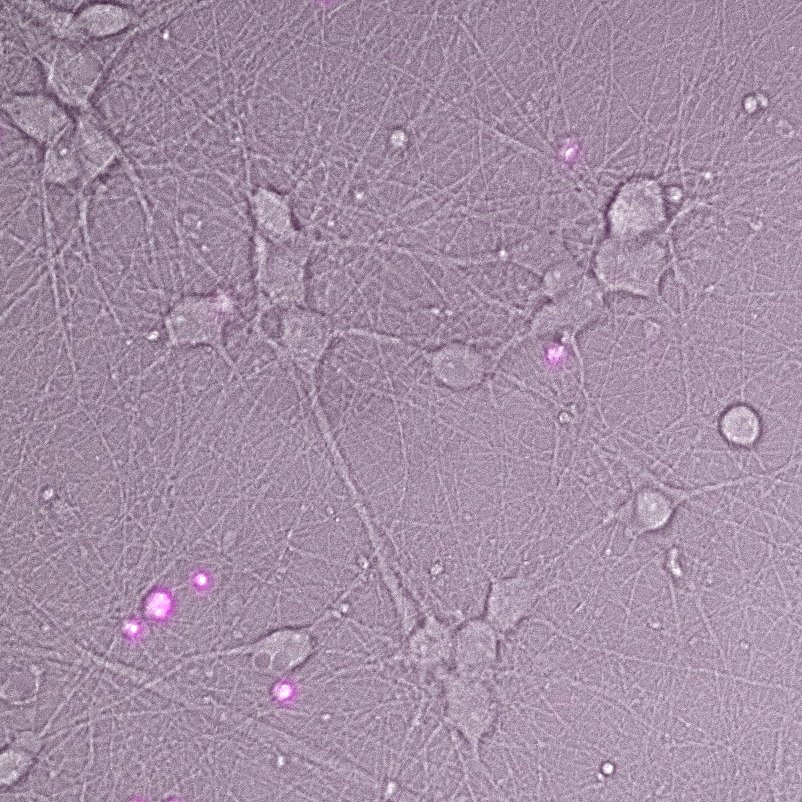

Supplement: Supplementary file 9 — Figure EVs Source Data [file 44319_2024_140_MOESM9_ESM.zip › SD EV figures/Supplementary Figure 3 - EV3/Panel S3B/representative images/cropped/G3_A92_0h-1.jpg]

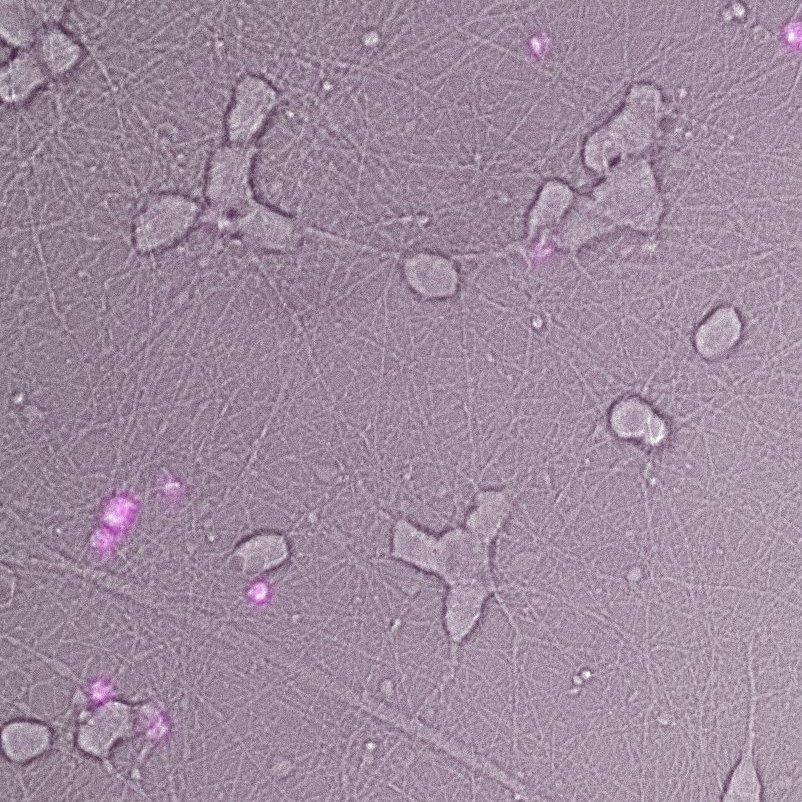

Supplement: Supplementary file 9 — Figure EVs Source Data [file 44319_2024_140_MOESM9_ESM.zip › SD EV figures/Supplementary Figure 3 - EV3/Panel S3B/representative images/cropped/G3_A92_48h-1.jpg]
