# Supplementary figures and images for: Glucose hypometabolism prompts RAN translation and exacerbates C9orf72-related ALS/FTD phenotypes (part 1 of 2)
Source: EMBO Rep. 2024 Apr 29;25(5):21. doi: 10.1038/s44319-024-00140-7 (PMC11094177; doi:10.1038/s44319-024-00140-7)

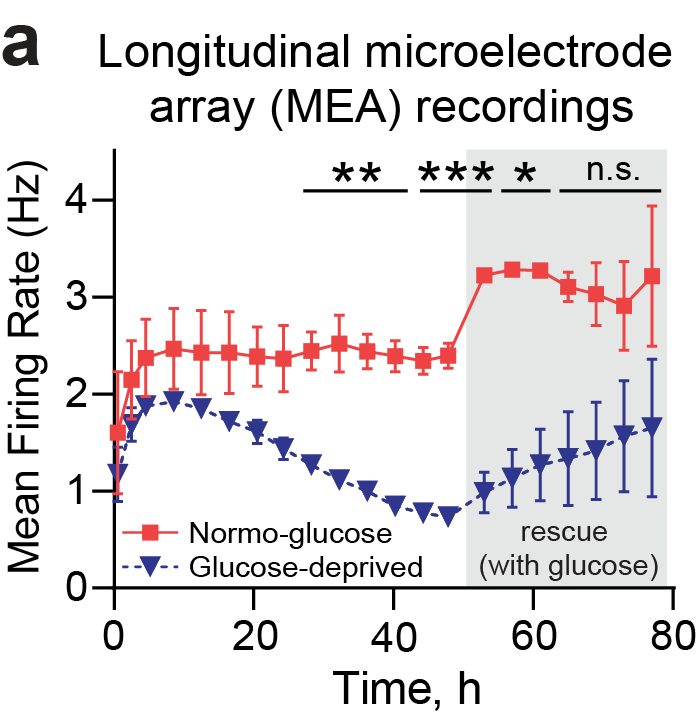

Supplement: Supplementary file 9 — Figure EVs Source Data [file 44319_2024_140_MOESM9_ESM.zip › SD EV figures/Supplementary Figure 4 - EV4/MEA data with rescue.png]

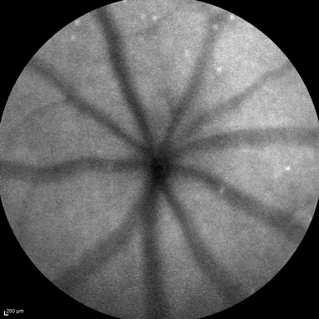

Supplement: Supplementary file 9 — Figure EVs Source Data [file 44319_2024_140_MOESM9_ESM.zip › SD EV figures/Supplementary Figure 6 - EV6/Panel S6B/OCT C9-2dg-2780 outer retina.tif]

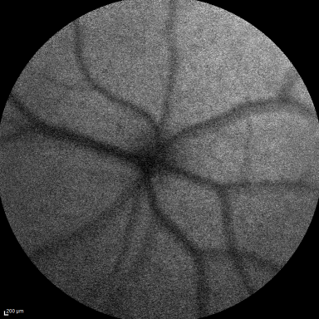

Supplement: Supplementary file 9 — Figure EVs Source Data [file 44319_2024_140_MOESM9_ESM.zip › SD EV figures/Supplementary Figure 6 - EV6/Panel S6B/OCT C9-saline-2785 outer retina.tif]

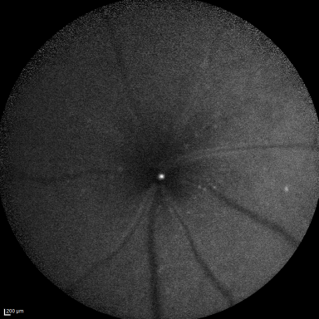

Supplement: Supplementary file 9 — Figure EVs Source Data [file 44319_2024_140_MOESM9_ESM.zip › SD EV figures/Supplementary Figure 6 - EV6/Panel S6B/OCT C9-2dg-2780 inner retina.tif]

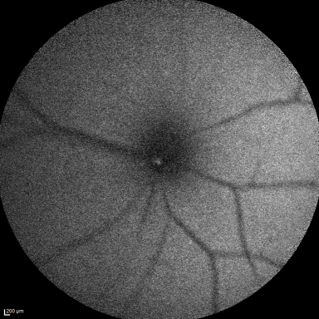

Supplement: Supplementary file 9 — Figure EVs Source Data [file 44319_2024_140_MOESM9_ESM.zip › SD EV figures/Supplementary Figure 6 - EV6/Panel S6B/OCT C9-saline-2785 inner retina.tif]

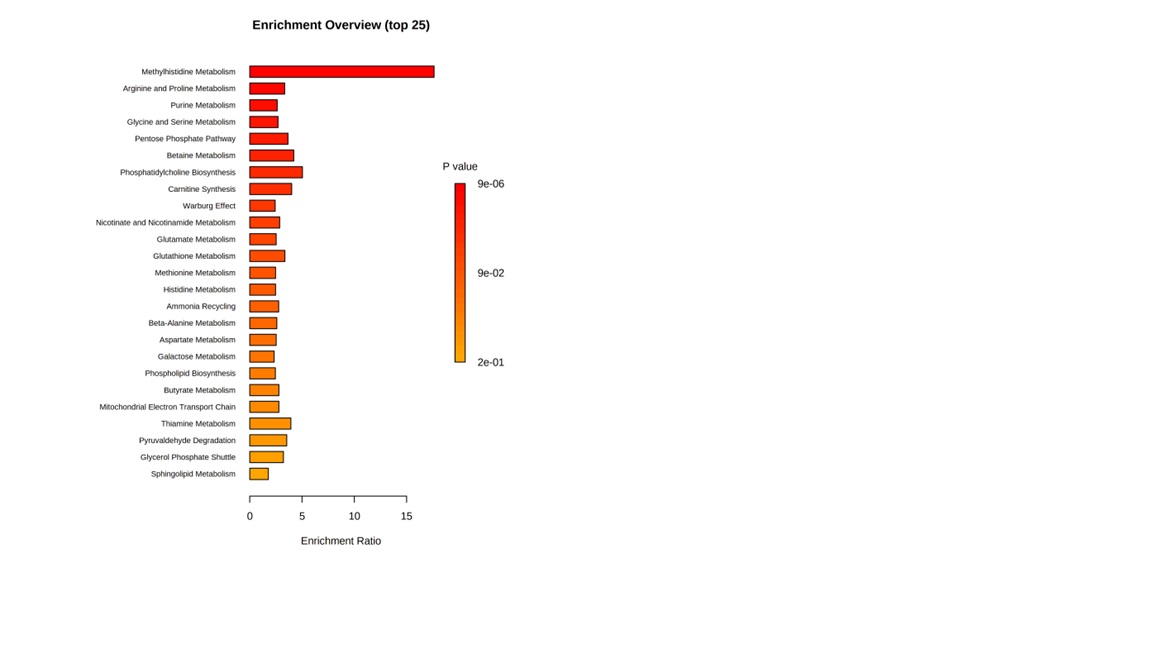

Supplement: Supplementary file 9 — Figure EVs Source Data [file 44319_2024_140_MOESM9_ESM.zip › SD EV figures/Supplementary Figure 6 - EV6/Panel S6A/enriched metabolite sets in 2DG animals (decreased) - plot from metaboanalyst.jpg]

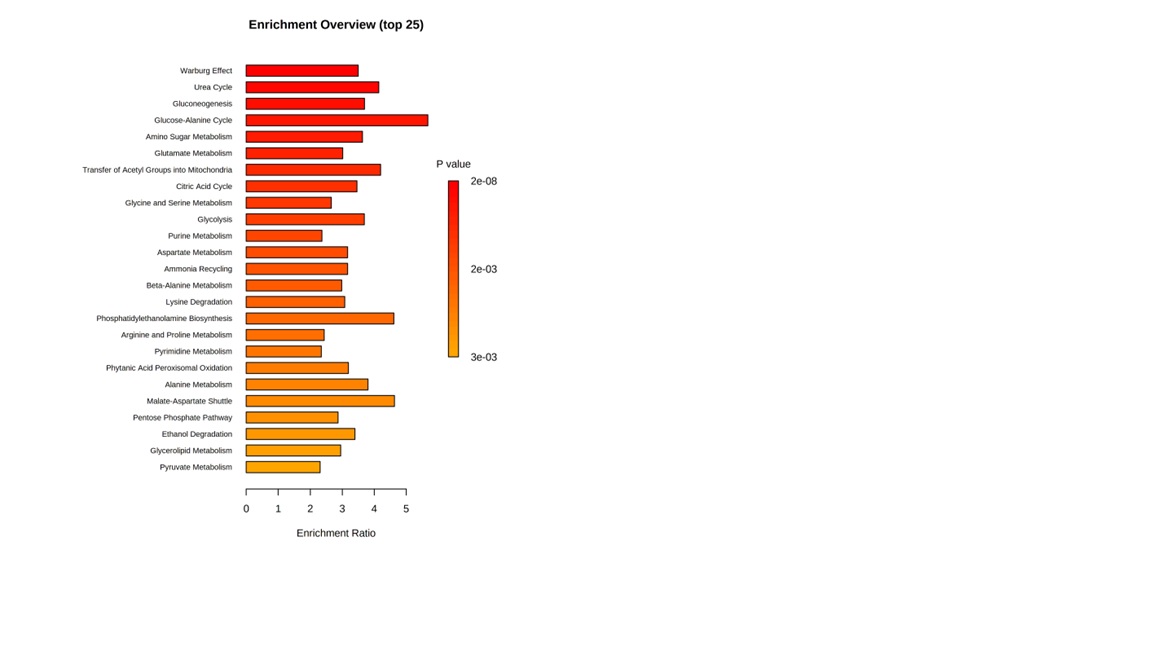

Supplement: Supplementary file 9 — Figure EVs Source Data [file 44319_2024_140_MOESM9_ESM.zip › SD EV figures/Supplementary Figure 6 - EV6/Panel S6A/enriched metabolite sets in 2DG animals (increased) - plot from metaboanalyst.jpg]

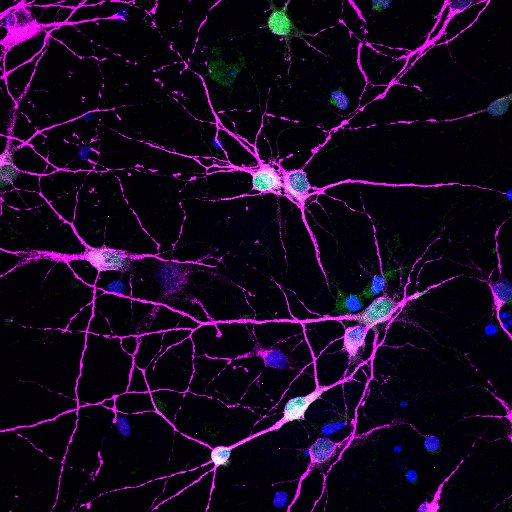

Supplement: Supplementary file 9 — Figure EVs Source Data [file 44319_2024_140_MOESM9_ESM.zip › SD EV figures/Supplementary Figure 7 - EV7/Panel S7B/merge GA.jpg]

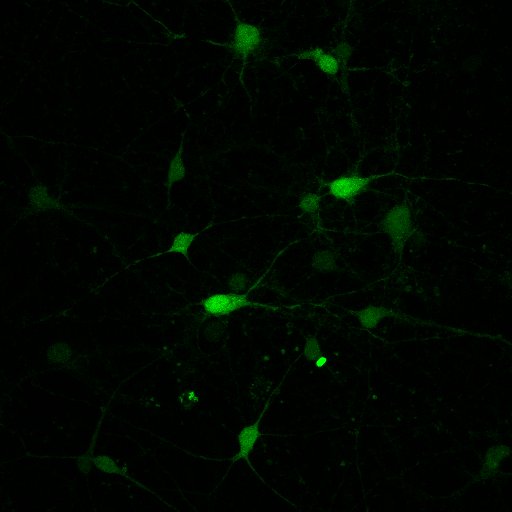

Supplement: Supplementary file 9 — Figure EVs Source Data [file 44319_2024_140_MOESM9_ESM.zip › SD EV figures/Supplementary Figure 7 - EV7/Panel S7B/C2-Primary_CNs_04_08_21_GFP_MAP2_ATF4_512_1_2-MaxIP.jpg]

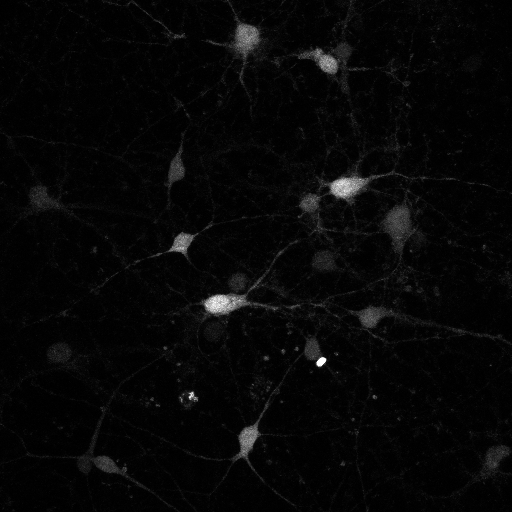

Supplement: Supplementary file 9 — Figure EVs Source Data [file 44319_2024_140_MOESM9_ESM.zip › SD EV figures/Supplementary Figure 7 - EV7/Panel S7B/merge GFP.tif]

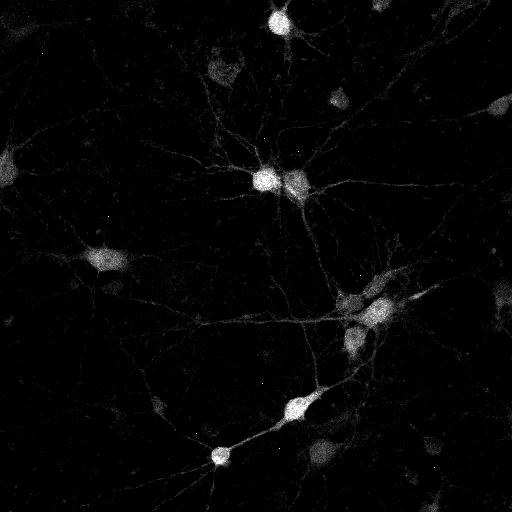

Supplement: Supplementary file 9 — Figure EVs Source Data [file 44319_2024_140_MOESM9_ESM.zip › SD EV figures/Supplementary Figure 7 - EV7/Panel S7B/C2-Primary_CNs_04_08_21_GA50-GFP_MAP2_ATF4_512_1_1-MaxIP.tif]

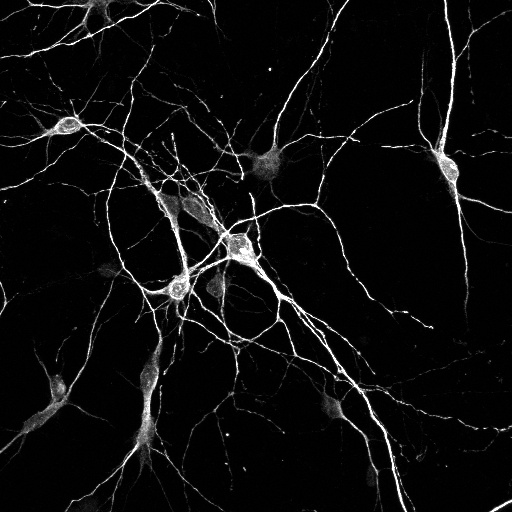

Supplement: Supplementary file 9 — Figure EVs Source Data [file 44319_2024_140_MOESM9_ESM.zip › SD EV figures/Supplementary Figure 7 - EV7/Panel S7B/C4-Primary_CNs_04_08_21_GR50-GFP_MAP2_ATF4_512_1_4-MaxIP.tif]

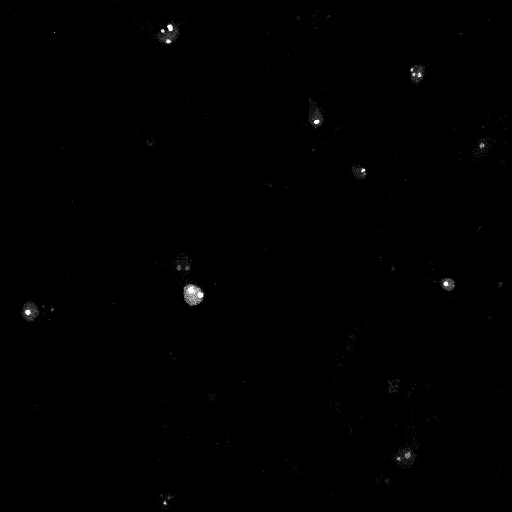

Supplement: Supplementary file 9 — Figure EVs Source Data [file 44319_2024_140_MOESM9_ESM.zip › SD EV figures/Supplementary Figure 7 - EV7/Panel S7B/C2-Primary_CNs_04_08_21_PR50-GFP_MAP2_ATF4_512_1_1-MaxIP.tif]

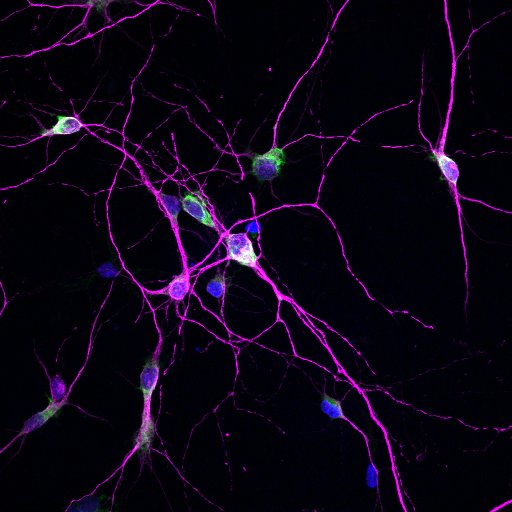

Supplement: Supplementary file 9 — Figure EVs Source Data [file 44319_2024_140_MOESM9_ESM.zip › SD EV figures/Supplementary Figure 7 - EV7/Panel S7B/merge GR.jpg]

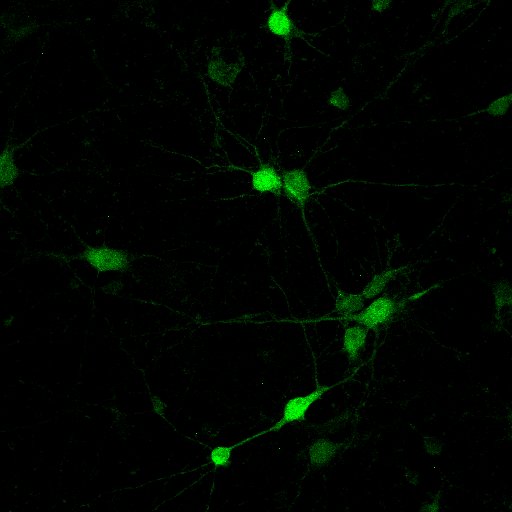

Supplement: Supplementary file 9 — Figure EVs Source Data [file 44319_2024_140_MOESM9_ESM.zip › SD EV figures/Supplementary Figure 7 - EV7/Panel S7B/C2-Primary_CNs_04_08_21_GA50-GFP_MAP2_ATF4_512_1_1-MaxIP.jpg]

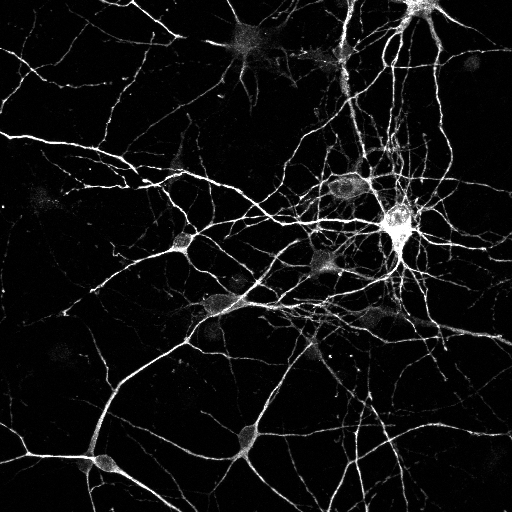

Supplement: Supplementary file 9 — Figure EVs Source Data [file 44319_2024_140_MOESM9_ESM.zip › SD EV figures/Supplementary Figure 7 - EV7/Panel S7B/C4-Primary_CNs_04_08_21_GFP_MAP2_ATF4_512_1_2-MaxIP.tif]

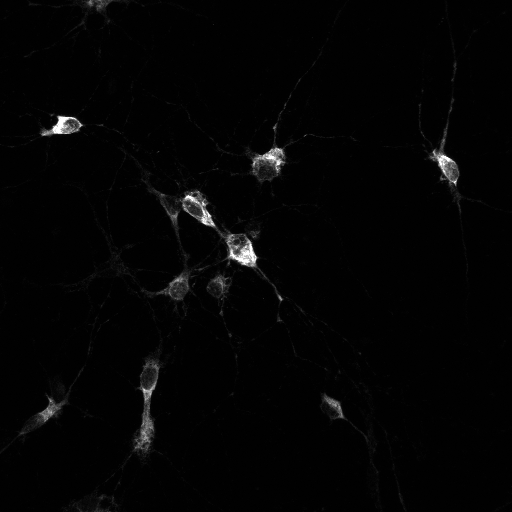

Supplement: Supplementary file 9 — Figure EVs Source Data [file 44319_2024_140_MOESM9_ESM.zip › SD EV figures/Supplementary Figure 7 - EV7/Panel S7B/merge GR.tif]

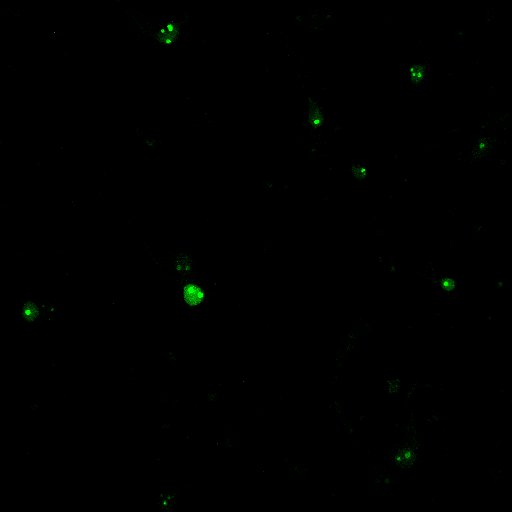

Supplement: Supplementary file 9 — Figure EVs Source Data [file 44319_2024_140_MOESM9_ESM.zip › SD EV figures/Supplementary Figure 7 - EV7/Panel S7B/C2-Primary_CNs_04_08_21_PR50-GFP_MAP2_ATF4_512_1_1-MaxIP.jpg]

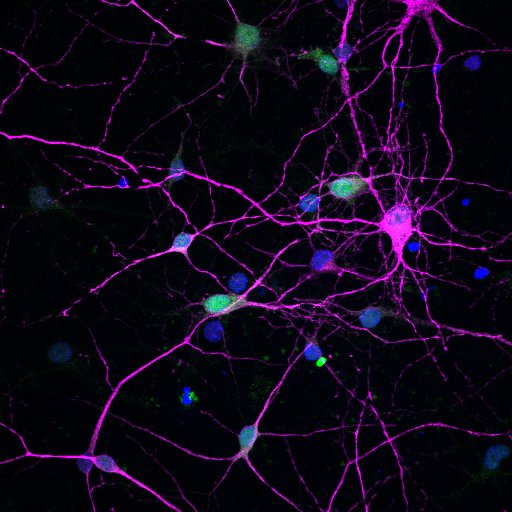

Supplement: Supplementary file 9 — Figure EVs Source Data [file 44319_2024_140_MOESM9_ESM.zip › SD EV figures/Supplementary Figure 7 - EV7/Panel S7B/merge GFP.jpg]

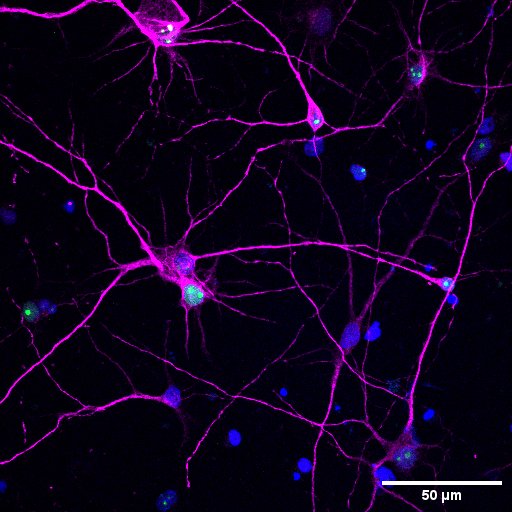

Supplement: Supplementary file 9 — Figure EVs Source Data [file 44319_2024_140_MOESM9_ESM.zip › SD EV figures/Supplementary Figure 7 - EV7/Panel S7B/merge PR_SB.jpg]

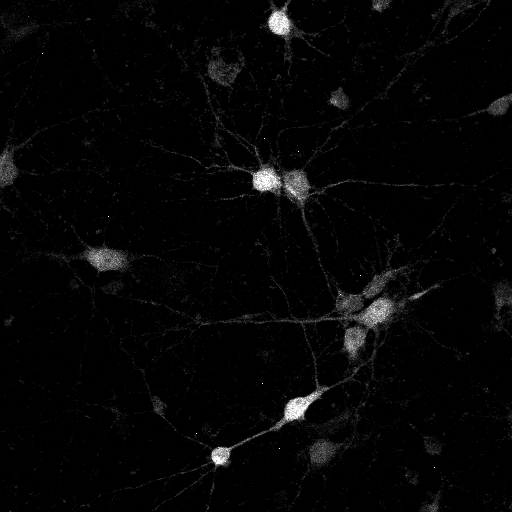

Supplement: Supplementary file 9 — Figure EVs Source Data [file 44319_2024_140_MOESM9_ESM.zip › SD EV figures/Supplementary Figure 7 - EV7/Panel S7B/merge GA.tif]

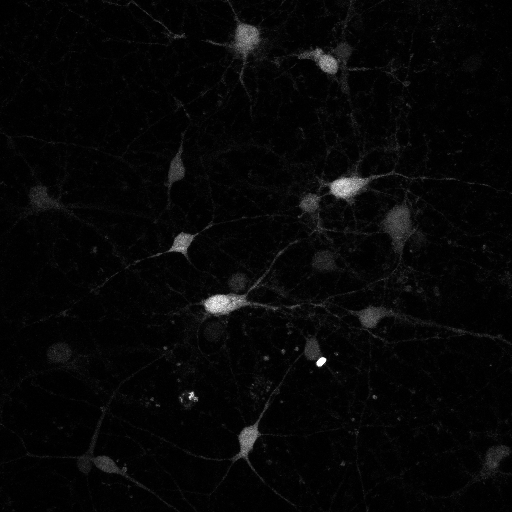

Supplement: Supplementary file 9 — Figure EVs Source Data [file 44319_2024_140_MOESM9_ESM.zip › SD EV figures/Supplementary Figure 7 - EV7/Panel S7B/C2-Primary_CNs_04_08_21_GFP_MAP2_ATF4_512_1_2-MaxIP.tif]

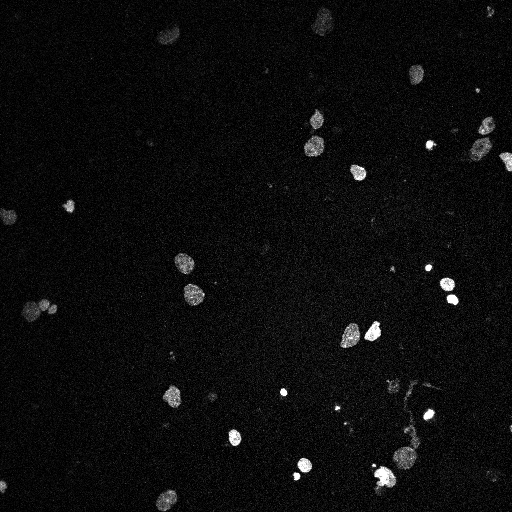

Supplement: Supplementary file 9 — Figure EVs Source Data [file 44319_2024_140_MOESM9_ESM.zip › SD EV figures/Supplementary Figure 7 - EV7/Panel S7B/C1-Primary_CNs_04_08_21_PR50-GFP_MAP2_ATF4_512_1_1-MaxIP.tif]

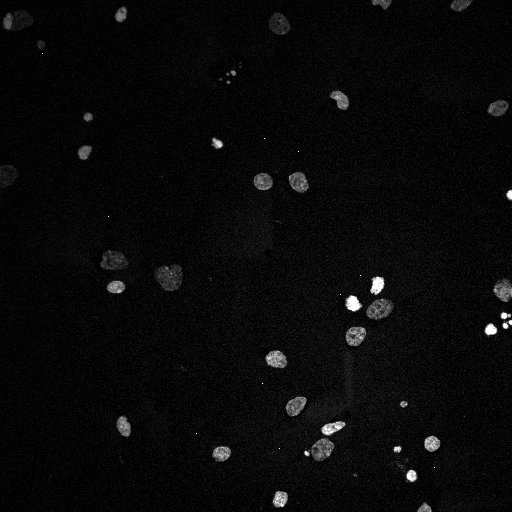

Supplement: Supplementary file 9 — Figure EVs Source Data [file 44319_2024_140_MOESM9_ESM.zip › SD EV figures/Supplementary Figure 7 - EV7/Panel S7B/C1-Primary_CNs_04_08_21_GA50-GFP_MAP2_ATF4_512_1_1-MaxIP.tif]

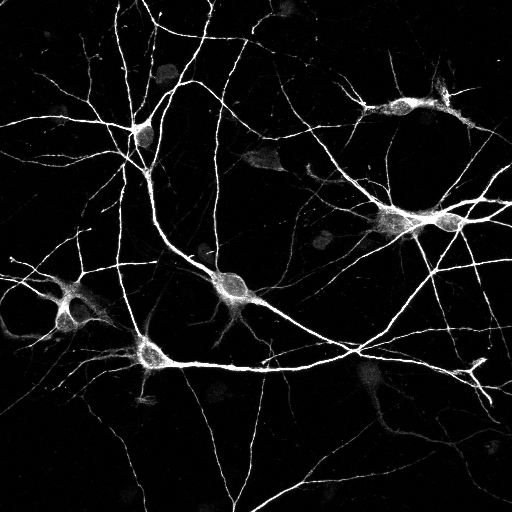

Supplement: Supplementary file 9 — Figure EVs Source Data [file 44319_2024_140_MOESM9_ESM.zip › SD EV figures/Supplementary Figure 7 - EV7/Panel S7B/C4-Primary_CNs_04_08_21_PA50-GFP_MAP2_ATF4_512_1_5-MaxIP.tif]

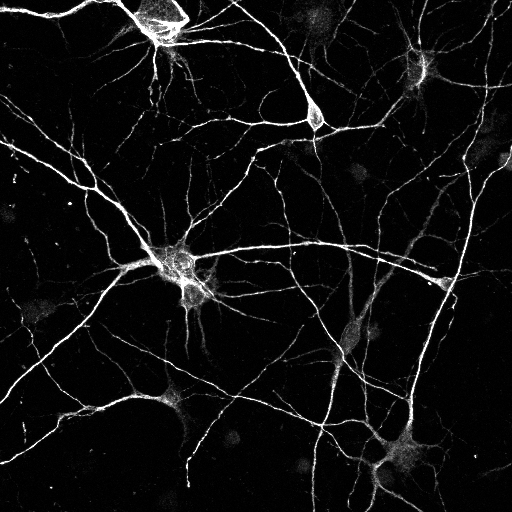

Supplement: Supplementary file 9 — Figure EVs Source Data [file 44319_2024_140_MOESM9_ESM.zip › SD EV figures/Supplementary Figure 7 - EV7/Panel S7B/C4-Primary_CNs_04_08_21_PR50-GFP_MAP2_ATF4_512_1_1-MaxIP.tif]

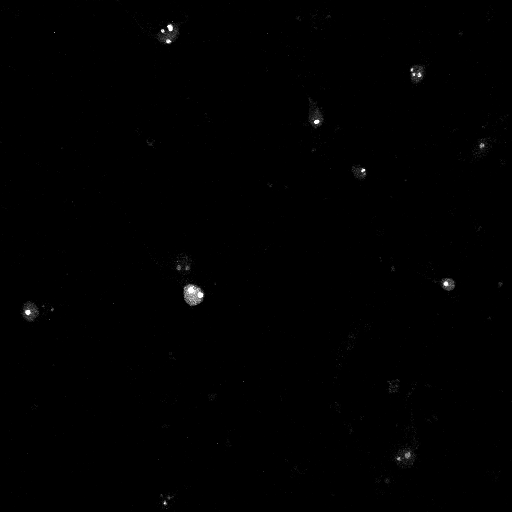

Supplement: Supplementary file 9 — Figure EVs Source Data [file 44319_2024_140_MOESM9_ESM.zip › SD EV figures/Supplementary Figure 7 - EV7/Panel S7B/merge PR.tif]

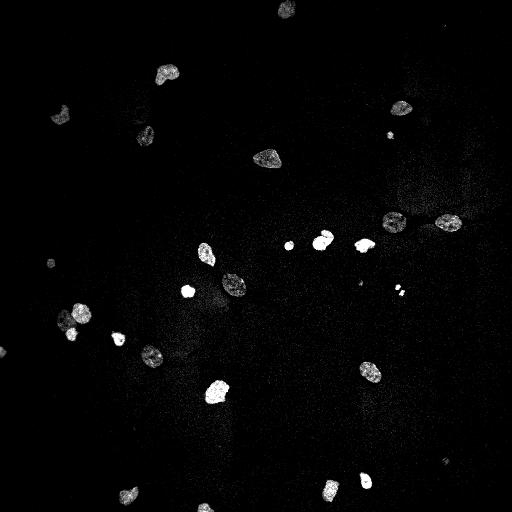

Supplement: Supplementary file 9 — Figure EVs Source Data [file 44319_2024_140_MOESM9_ESM.zip › SD EV figures/Supplementary Figure 7 - EV7/Panel S7B/C1-Primary_CNs_04_08_21_PA50-GFP_MAP2_ATF4_512_1_5-MaxIP.tif]

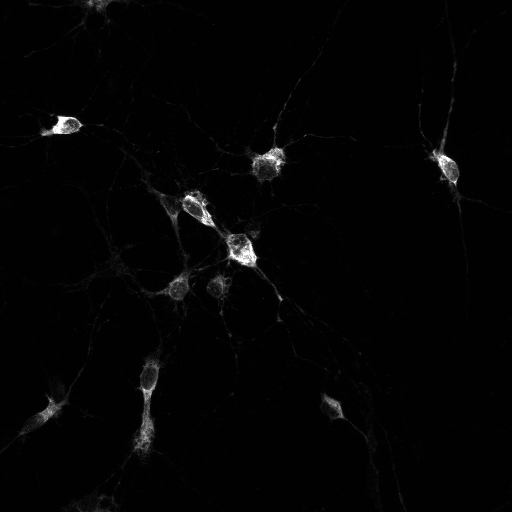

Supplement: Supplementary file 9 — Figure EVs Source Data [file 44319_2024_140_MOESM9_ESM.zip › SD EV figures/Supplementary Figure 7 - EV7/Panel S7B/C2-Primary_CNs_04_08_21_GR50-GFP_MAP2_ATF4_512_1_4-MaxIP.tif]

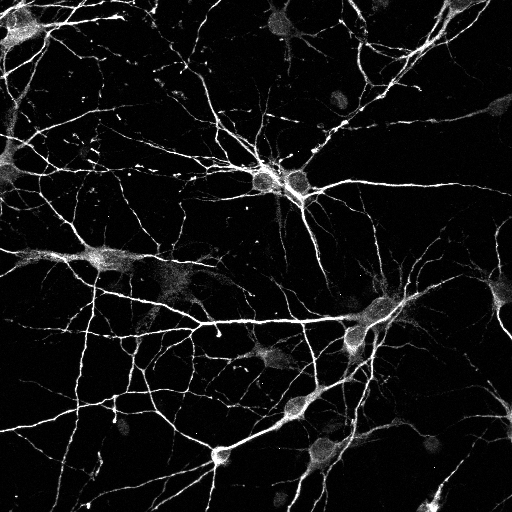

Supplement: Supplementary file 9 — Figure EVs Source Data [file 44319_2024_140_MOESM9_ESM.zip › SD EV figures/Supplementary Figure 7 - EV7/Panel S7B/C4-Primary_CNs_04_08_21_GA50-GFP_MAP2_ATF4_512_1_1-MaxIP.tif]

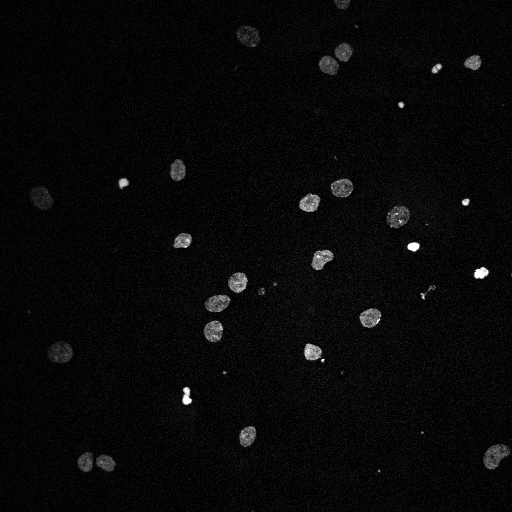

Supplement: Supplementary file 9 — Figure EVs Source Data [file 44319_2024_140_MOESM9_ESM.zip › SD EV figures/Supplementary Figure 7 - EV7/Panel S7B/C1-Primary_CNs_04_08_21_GFP_MAP2_ATF4_512_1_2-MaxIP.tif]

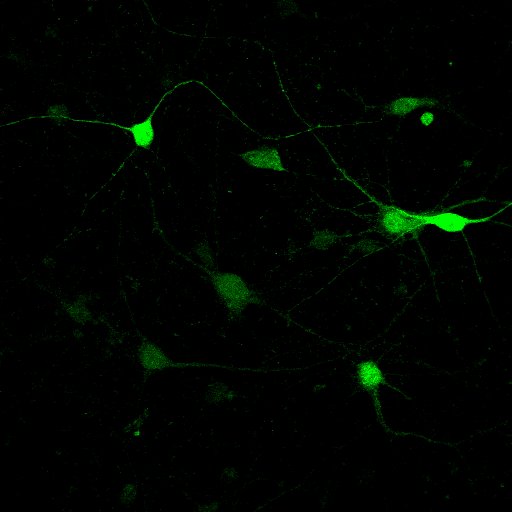

Supplement: Supplementary file 9 — Figure EVs Source Data [file 44319_2024_140_MOESM9_ESM.zip › SD EV figures/Supplementary Figure 7 - EV7/Panel S7B/C2-Primary_CNs_04_08_21_PA50-GFP_MAP2_ATF4_512_1_5-MaxIP.jpg]

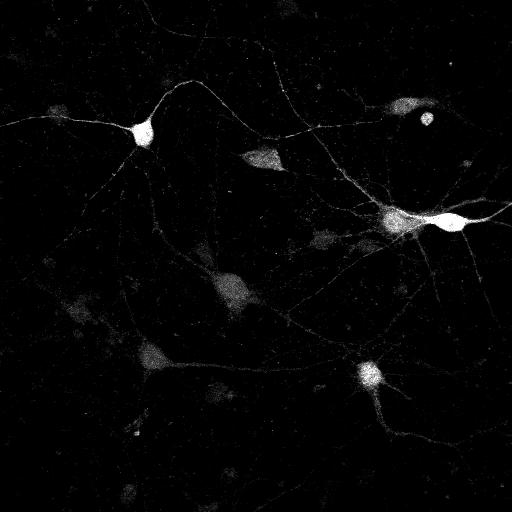

Supplement: Supplementary file 9 — Figure EVs Source Data [file 44319_2024_140_MOESM9_ESM.zip › SD EV figures/Supplementary Figure 7 - EV7/Panel S7B/merge PA.tif]

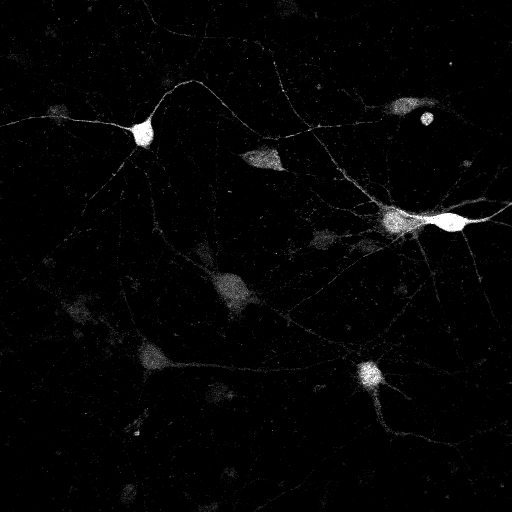

Supplement: Supplementary file 9 — Figure EVs Source Data [file 44319_2024_140_MOESM9_ESM.zip › SD EV figures/Supplementary Figure 7 - EV7/Panel S7B/C2-Primary_CNs_04_08_21_PA50-GFP_MAP2_ATF4_512_1_5-MaxIP.tif]

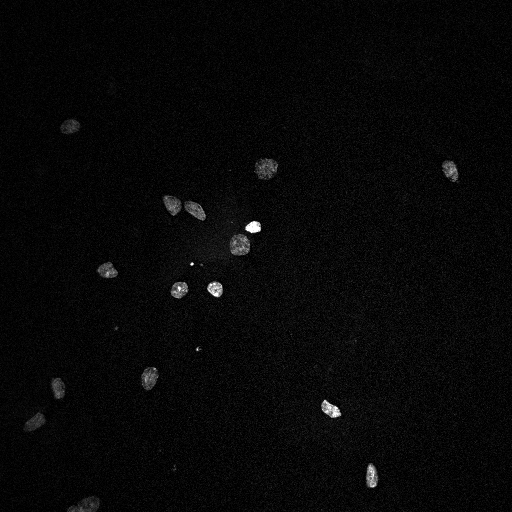

Supplement: Supplementary file 9 — Figure EVs Source Data [file 44319_2024_140_MOESM9_ESM.zip › SD EV figures/Supplementary Figure 7 - EV7/Panel S7B/C1-Primary_CNs_04_08_21_GR50-GFP_MAP2_ATF4_512_1_4-MaxIP.tif]

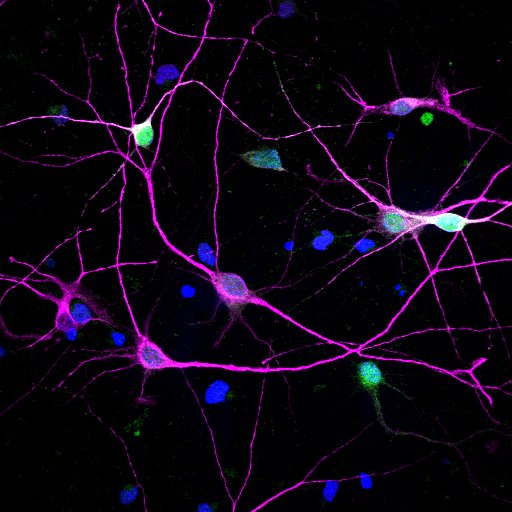

Supplement: Supplementary file 9 — Figure EVs Source Data [file 44319_2024_140_MOESM9_ESM.zip › SD EV figures/Supplementary Figure 7 - EV7/Panel S7B/merge PA.jpg]

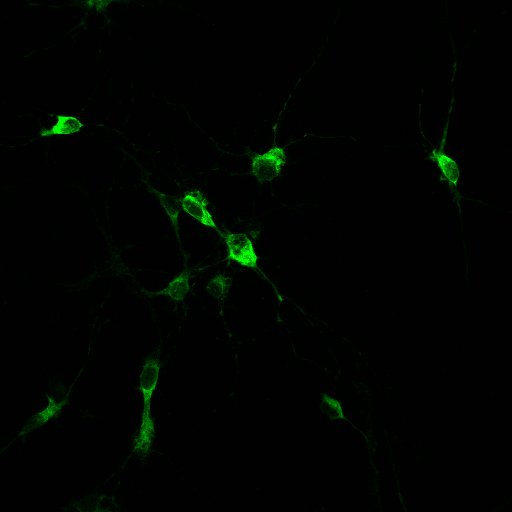

Supplement: Supplementary file 9 — Figure EVs Source Data [file 44319_2024_140_MOESM9_ESM.zip › SD EV figures/Supplementary Figure 7 - EV7/Panel S7B/C2-Primary_CNs_04_08_21_GR50-GFP_MAP2_ATF4_512_1_4-MaxIP.jpg]

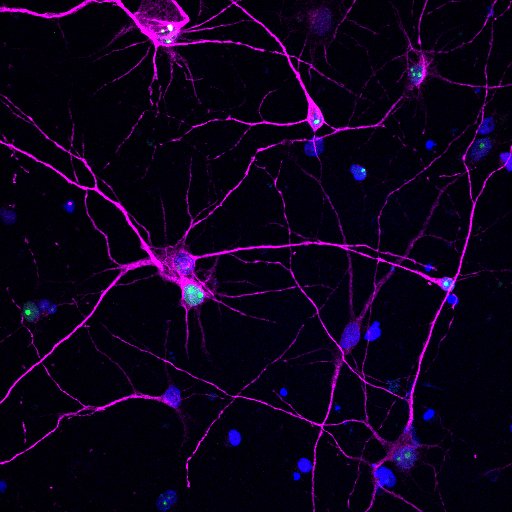

Supplement: Supplementary file 9 — Figure EVs Source Data [file 44319_2024_140_MOESM9_ESM.zip › SD EV figures/Supplementary Figure 7 - EV7/Panel S7B/merge PR.jpg]

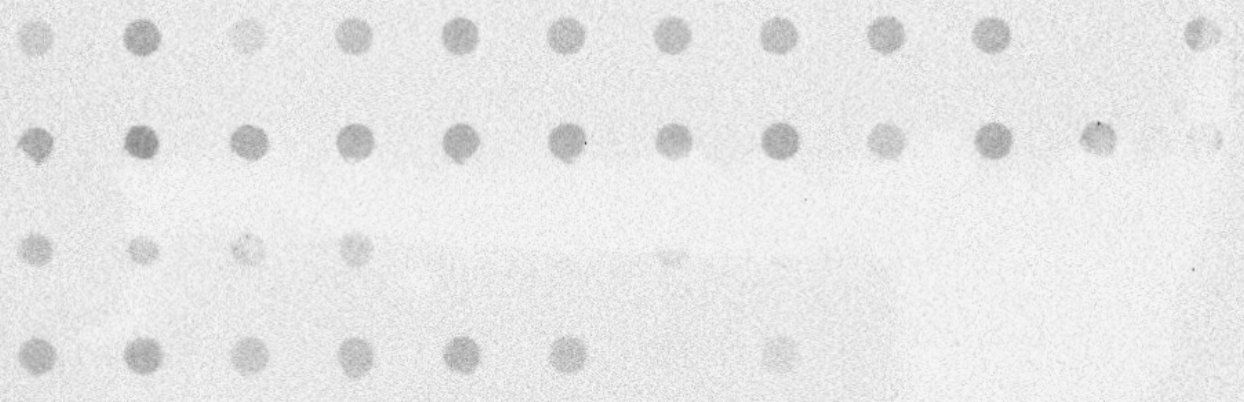

Supplement: Supplementary file 9 — Figure EVs Source Data [file 44319_2024_140_MOESM9_ESM.zip › SD EV figures/Supplementary Figure 5 - EV5/Panel S5E-F/M5_GP_otBlot copy.tif]

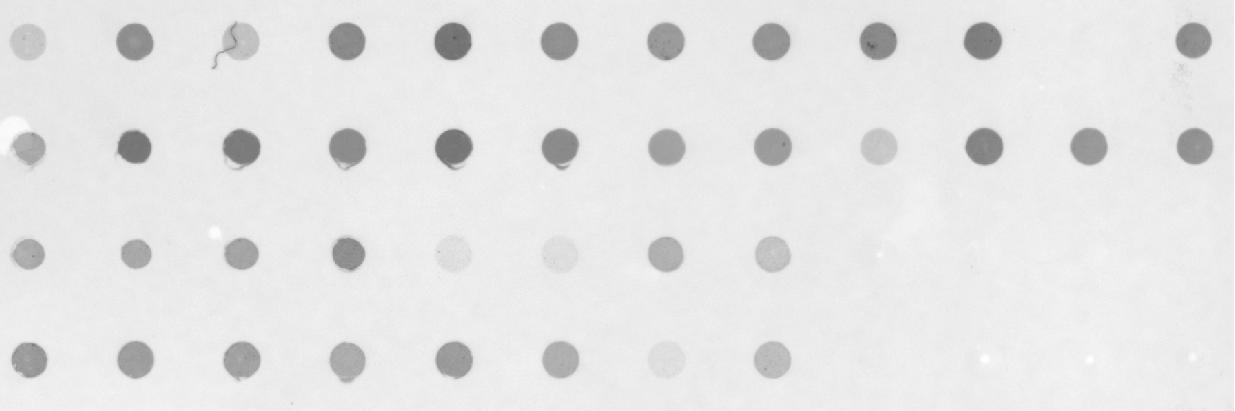

Supplement: Supplementary file 9 — Figure EVs Source Data [file 44319_2024_140_MOESM9_ESM.zip › SD EV figures/Supplementary Figure 5 - EV5/Panel S5E-F/M5_Ponceau copy.tif]

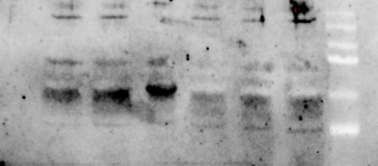

Supplement: Supplementary file 9 — Figure EVs Source Data [file 44319_2024_140_MOESM9_ESM.zip › SD EV figures/Supplementary Figure 3 - EV3/Panel S3A/ATF4 ALS SC tissue.tif]

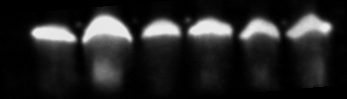

Supplement: Supplementary file 9 — Figure EVs Source Data [file 44319_2024_140_MOESM9_ESM.zip › SD EV figures/Supplementary Figure 3 - EV3/Panel S3A/Histone 3 ALS SC tissue (1) subbed and rotated.tif]

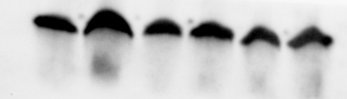

Supplement: Supplementary file 9 — Figure EVs Source Data [file 44319_2024_140_MOESM9_ESM.zip › SD EV figures/Supplementary Figure 3 - EV3/Panel S3A/Histone 3 ALS SC tissue.tif]

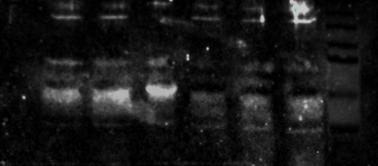

Supplement: Supplementary file 9 — Figure EVs Source Data [file 44319_2024_140_MOESM9_ESM.zip › SD EV figures/Supplementary Figure 3 - EV3/Panel S3A/ATF4 ALS SC tissue (1) subbed.tif]

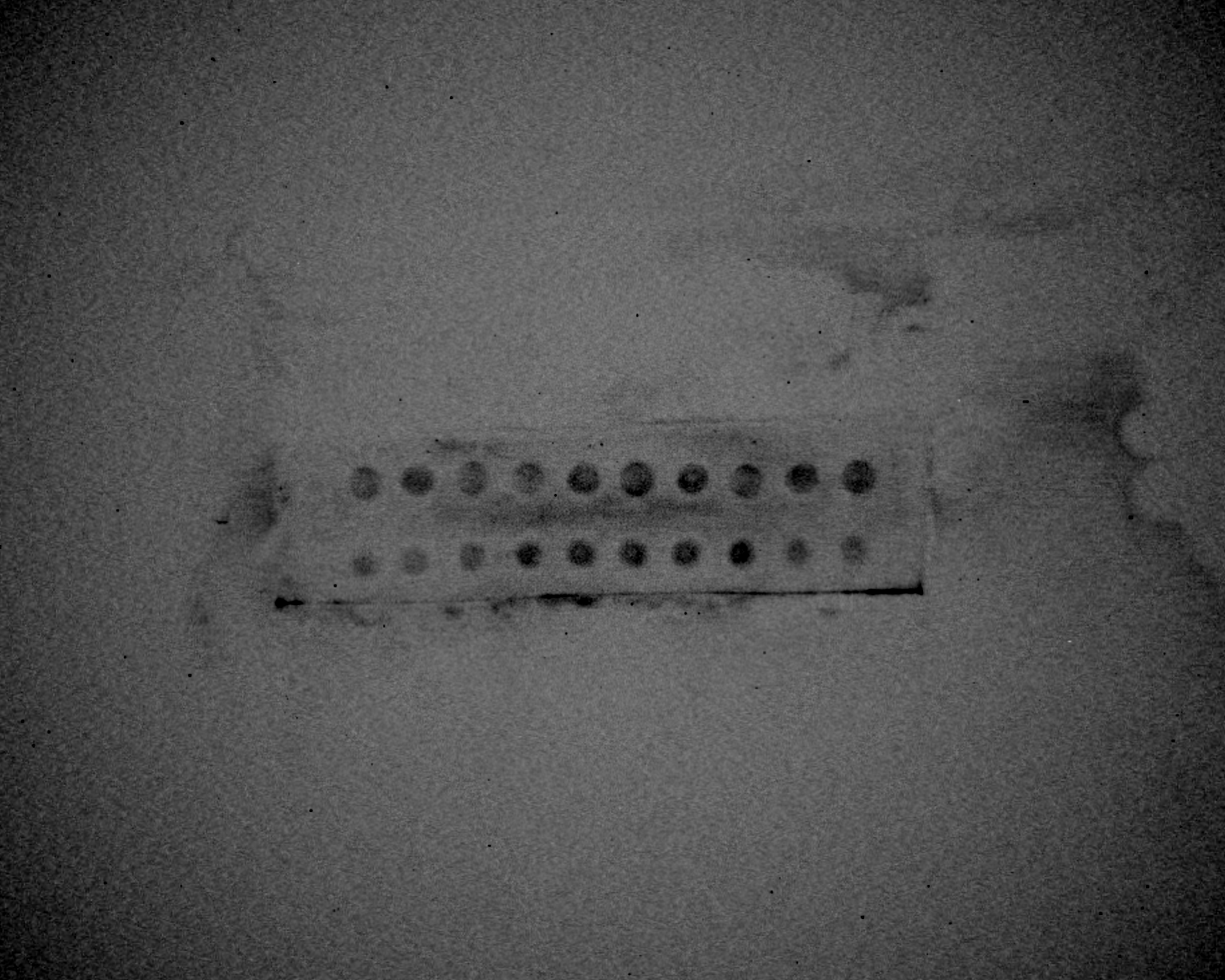

Supplement: Supplementary file 9 — Figure EVs Source Data [file 44319_2024_140_MOESM9_ESM.zip › SD EV figures/Supplementary Figure 6 - EV6/Panel S6E/additional repeat/Admin1 2022-10-10 20h39m01s(Chemiluminescence).tif]

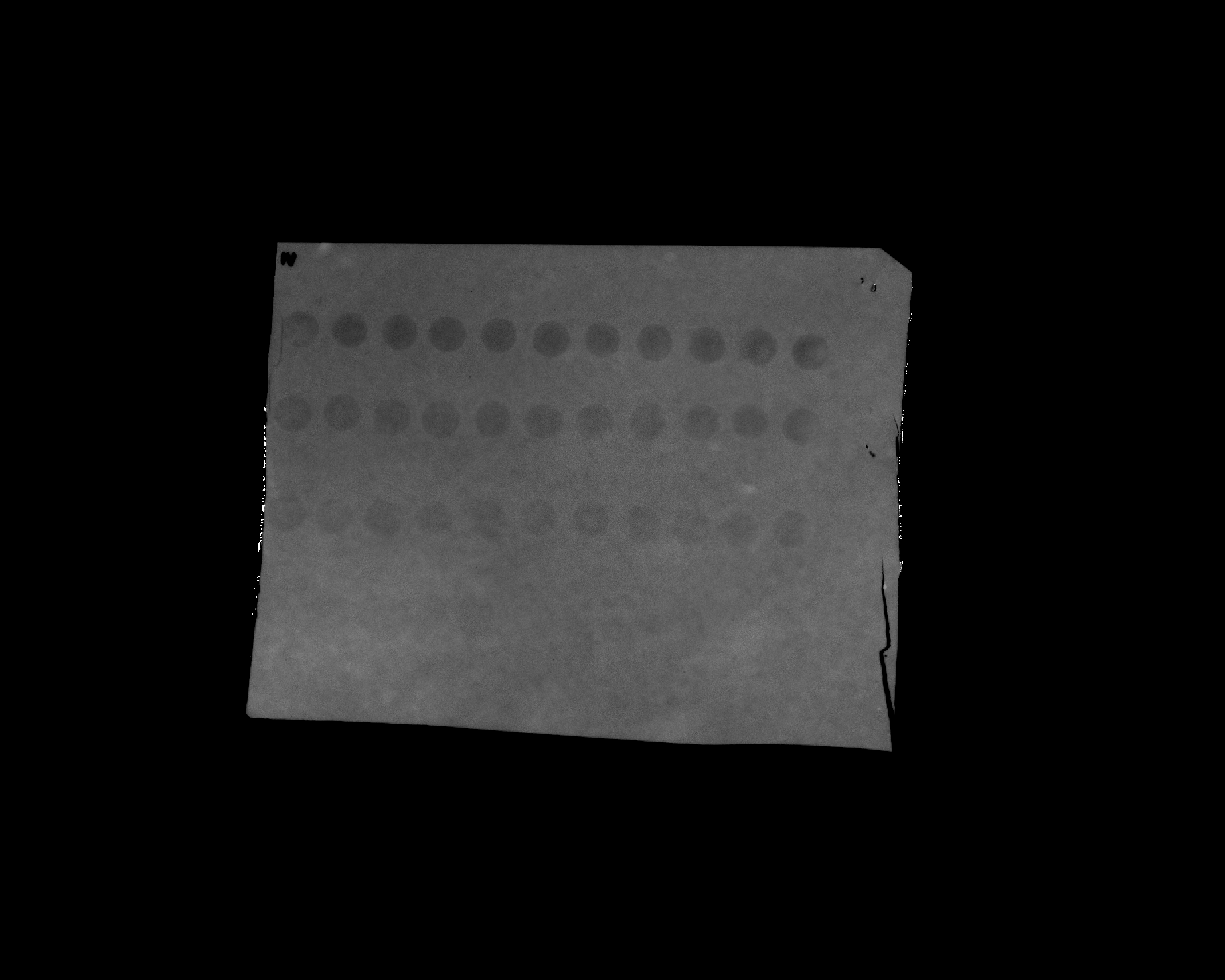

Supplement: Supplementary file 9 — Figure EVs Source Data [file 44319_2024_140_MOESM9_ESM.zip › SD EV figures/Supplementary Figure 6 - EV6/Panel S6E/additional repeat/Admin1 2022-10-10 15h15m14s(Colorimetric).tif]

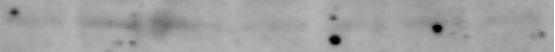

Supplement: Supplementary file 9 — Figure EVs Source Data [file 44319_2024_140_MOESM9_ESM.zip › SD EV figures/Supplementary Figure 6 - EV6/Panel S6F/MEC Figure EV 6 F/eifa m1-m4_6.tif]

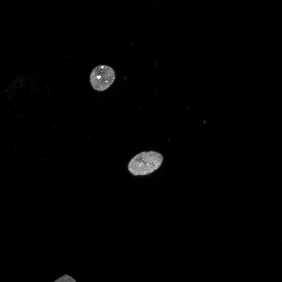

Supplement: Supplementary file 9 — Figure EVs Source Data [file 44319_2024_140_MOESM9_ESM.zip › SD EV figures/Supplementary Figure 4 - EV4/Panel S4D/representative images/C1-i3Neurons_10_25_21_Normo-glucose_3-MaxIP-1-1.tif]

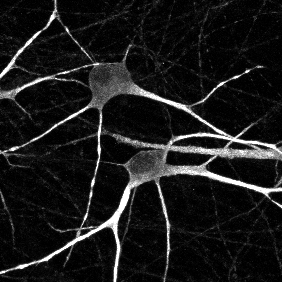

Supplement: Supplementary file 9 — Figure EVs Source Data [file 44319_2024_140_MOESM9_ESM.zip › SD EV figures/Supplementary Figure 4 - EV4/Panel S4D/representative images/C3-i3Neurons_10_25_21_Normo-glucose_3-MaxIP-1-1.tif]

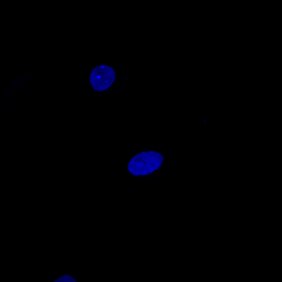

Supplement: Supplementary file 9 — Figure EVs Source Data [file 44319_2024_140_MOESM9_ESM.zip › SD EV figures/Supplementary Figure 4 - EV4/Panel S4D/representative images/C1-i3Neurons_10_25_21_Normo-glucose_3-MaxIP-1-1.jpg]

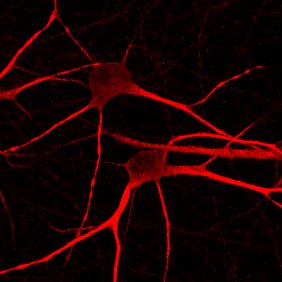

Supplement: Supplementary file 9 — Figure EVs Source Data [file 44319_2024_140_MOESM9_ESM.zip › SD EV figures/Supplementary Figure 4 - EV4/Panel S4D/representative images/C3-i3Neurons_10_25_21_Normo-glucose_3-MaxIP-1-1.jpg]

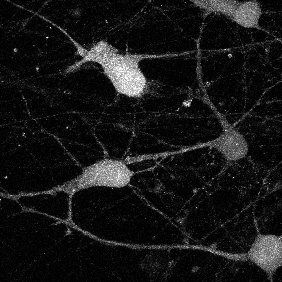

Supplement: Supplementary file 9 — Figure EVs Source Data [file 44319_2024_140_MOESM9_ESM.zip › SD EV figures/Supplementary Figure 4 - EV4/Panel S4D/representative images/C2-i3Neurons_11_22_21_Glucose-deprived_7-MaxIP-1.tif]

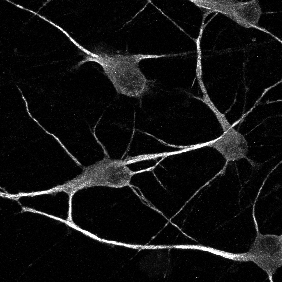

Supplement: Supplementary file 9 — Figure EVs Source Data [file 44319_2024_140_MOESM9_ESM.zip › SD EV figures/Supplementary Figure 4 - EV4/Panel S4D/representative images/C3-i3Neurons_11_22_21_Glucose-deprived_7-MaxIP-1.tif]

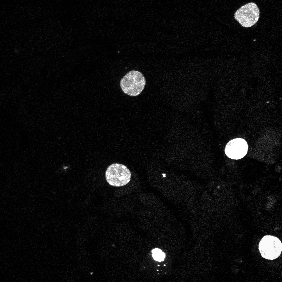

Supplement: Supplementary file 9 — Figure EVs Source Data [file 44319_2024_140_MOESM9_ESM.zip › SD EV figures/Supplementary Figure 4 - EV4/Panel S4D/representative images/C1-i3Neurons_11_22_21_Glucose-deprived_7-MaxIP-1.tif]

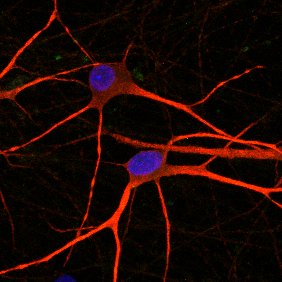

Supplement: Supplementary file 9 — Figure EVs Source Data [file 44319_2024_140_MOESM9_ESM.zip › SD EV figures/Supplementary Figure 4 - EV4/Panel S4D/representative images/MERGE-i3Neurons_10_25_21_Normo-glucose_3-MaxIP-1-1.jpg]

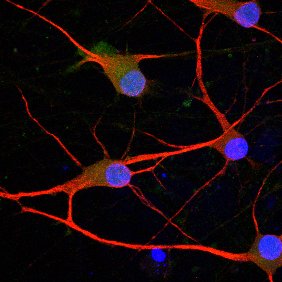

Supplement: Supplementary file 9 — Figure EVs Source Data [file 44319_2024_140_MOESM9_ESM.zip › SD EV figures/Supplementary Figure 4 - EV4/Panel S4D/representative images/MERGE-i3Neurons_11_22_21_Glucose-deprived_7-MaxIP-1.jpg]

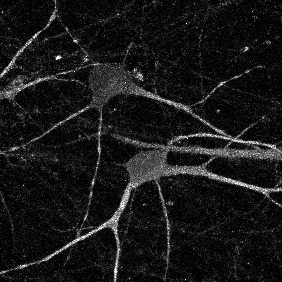

Supplement: Supplementary file 9 — Figure EVs Source Data [file 44319_2024_140_MOESM9_ESM.zip › SD EV figures/Supplementary Figure 4 - EV4/Panel S4D/representative images/C2-i3Neurons_10_25_21_Normo-glucose_3-MaxIP-1-1.tif]

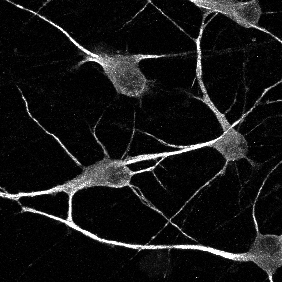

Supplement: Supplementary file 9 — Figure EVs Source Data [file 44319_2024_140_MOESM9_ESM.zip › SD EV figures/Supplementary Figure 4 - EV4/Panel S4D/representative images/MERGE-i3Neurons_11_22_21_Glucose-deprived_7-MaxIP-1.tif]

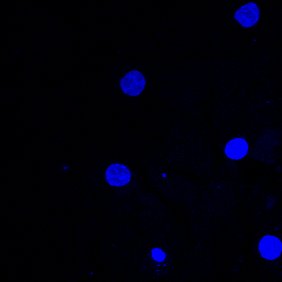

Supplement: Supplementary file 9 — Figure EVs Source Data [file 44319_2024_140_MOESM9_ESM.zip › SD EV figures/Supplementary Figure 4 - EV4/Panel S4D/representative images/C1-i3Neurons_11_22_21_Glucose-deprived_7-MaxIP-1.jpg]

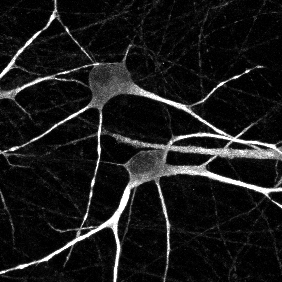

Supplement: Supplementary file 9 — Figure EVs Source Data [file 44319_2024_140_MOESM9_ESM.zip › SD EV figures/Supplementary Figure 4 - EV4/Panel S4D/representative images/MERGE-i3Neurons_10_25_21_Normo-glucose_3-MaxIP-1-1.tif]

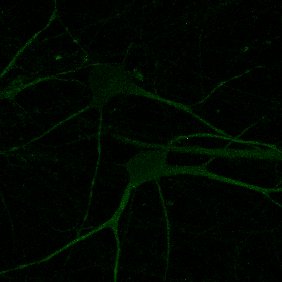

Supplement: Supplementary file 9 — Figure EVs Source Data [file 44319_2024_140_MOESM9_ESM.zip › SD EV figures/Supplementary Figure 4 - EV4/Panel S4D/representative images/C2-i3Neurons_10_25_21_Normo-glucose_3-MaxIP-1-1.jpg]

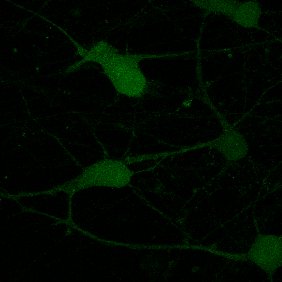

Supplement: Supplementary file 9 — Figure EVs Source Data [file 44319_2024_140_MOESM9_ESM.zip › SD EV figures/Supplementary Figure 4 - EV4/Panel S4D/representative images/C2-i3Neurons_11_22_21_Glucose-deprived_7-MaxIP-1.jpg]

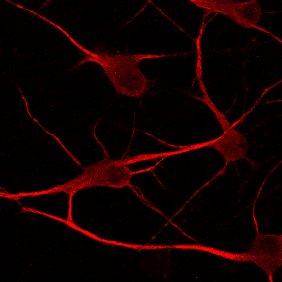

Supplement: Supplementary file 9 — Figure EVs Source Data [file 44319_2024_140_MOESM9_ESM.zip › SD EV figures/Supplementary Figure 4 - EV4/Panel S4D/representative images/C3-i3Neurons_11_22_21_Glucose-deprived_7-MaxIP-1.jpg]

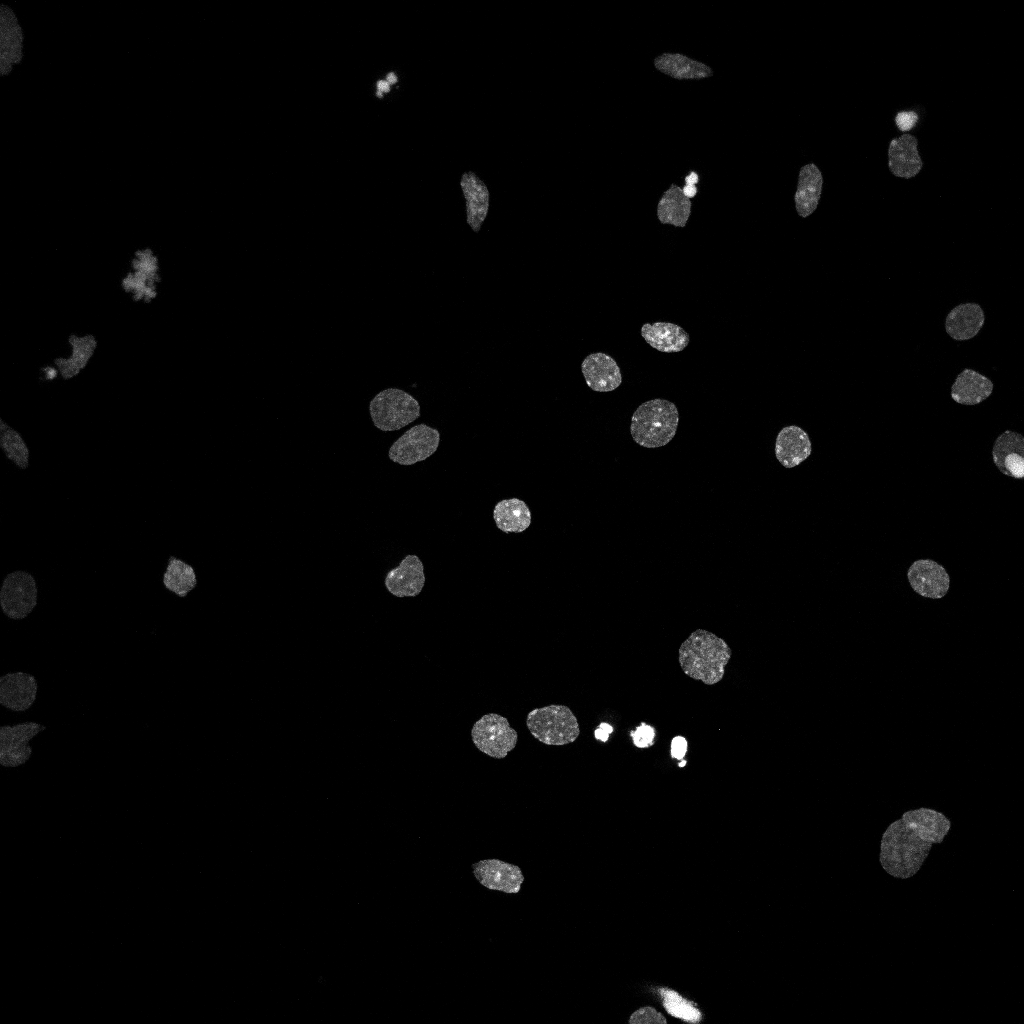

Supplement: Supplementary file 9 — Figure EVs Source Data [file 44319_2024_140_MOESM9_ESM.zip › SD EV figures/Supplementary Figure 3 - EV3/Panel S3C/raw images/MAX_RANT_ISRIB_compl.tif]

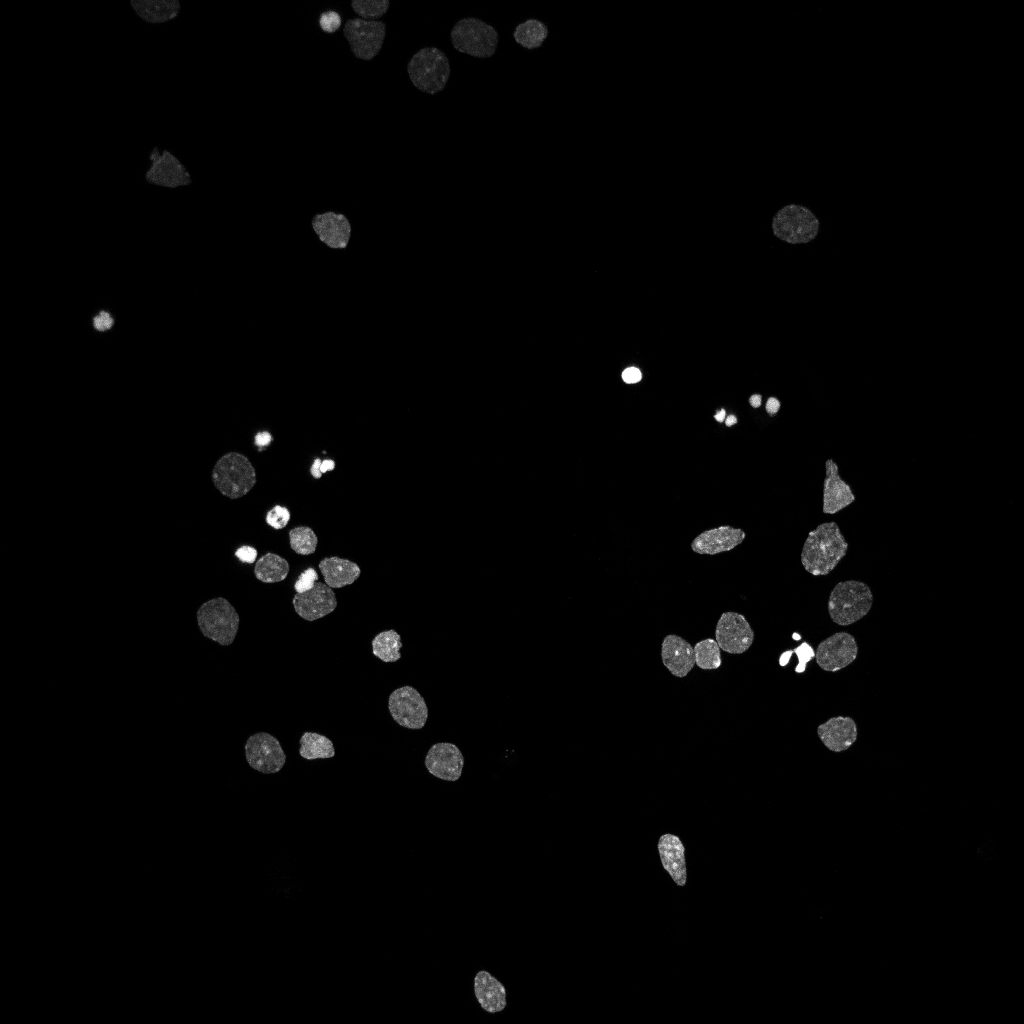

Supplement: Supplementary file 9 — Figure EVs Source Data [file 44319_2024_140_MOESM9_ESM.zip › SD EV figures/Supplementary Figure 3 - EV3/Panel S3C/raw images/MAX_RANT_ISRIB_2DG+ISRIB.tif]

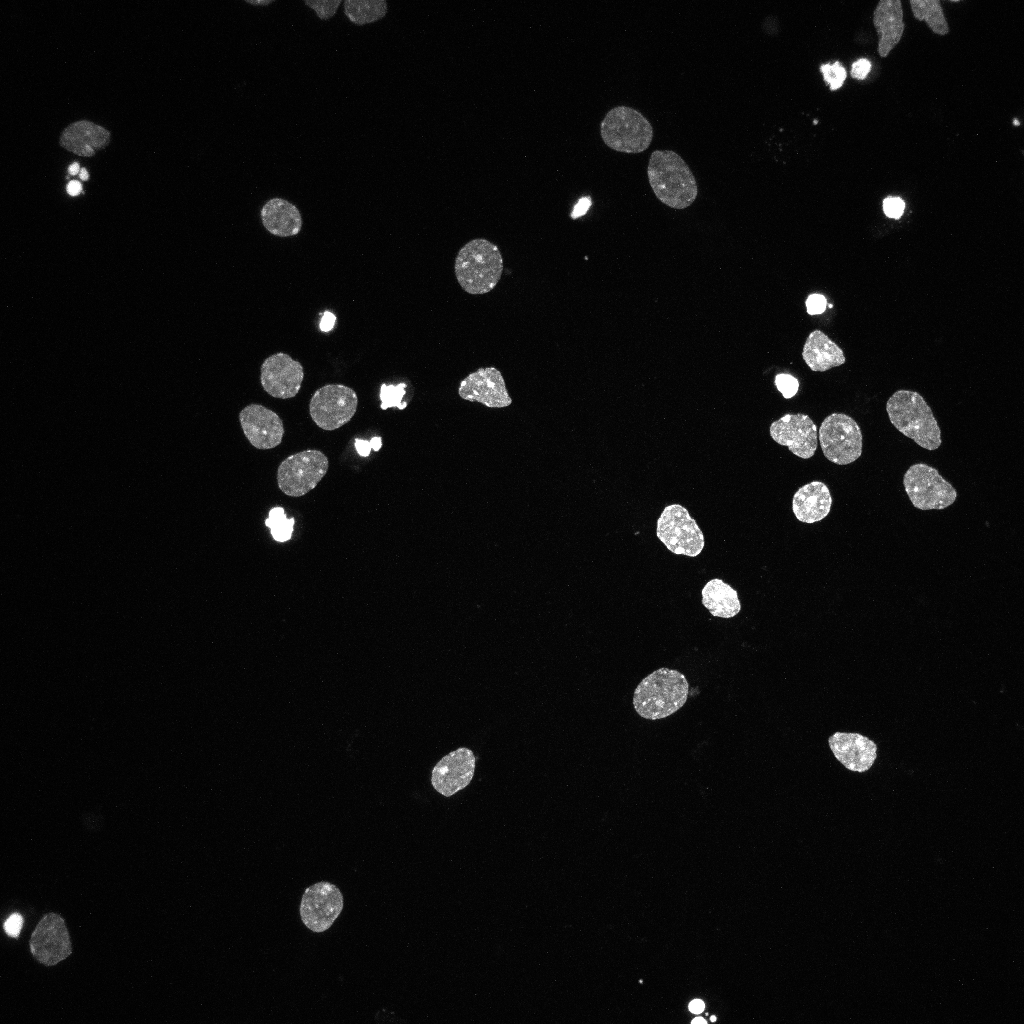

Supplement: Supplementary file 9 — Figure EVs Source Data [file 44319_2024_140_MOESM9_ESM.zip › SD EV figures/Supplementary Figure 3 - EV3/Panel S3C/raw images/MAX_RANT_ISRIB_2DG001.tif]

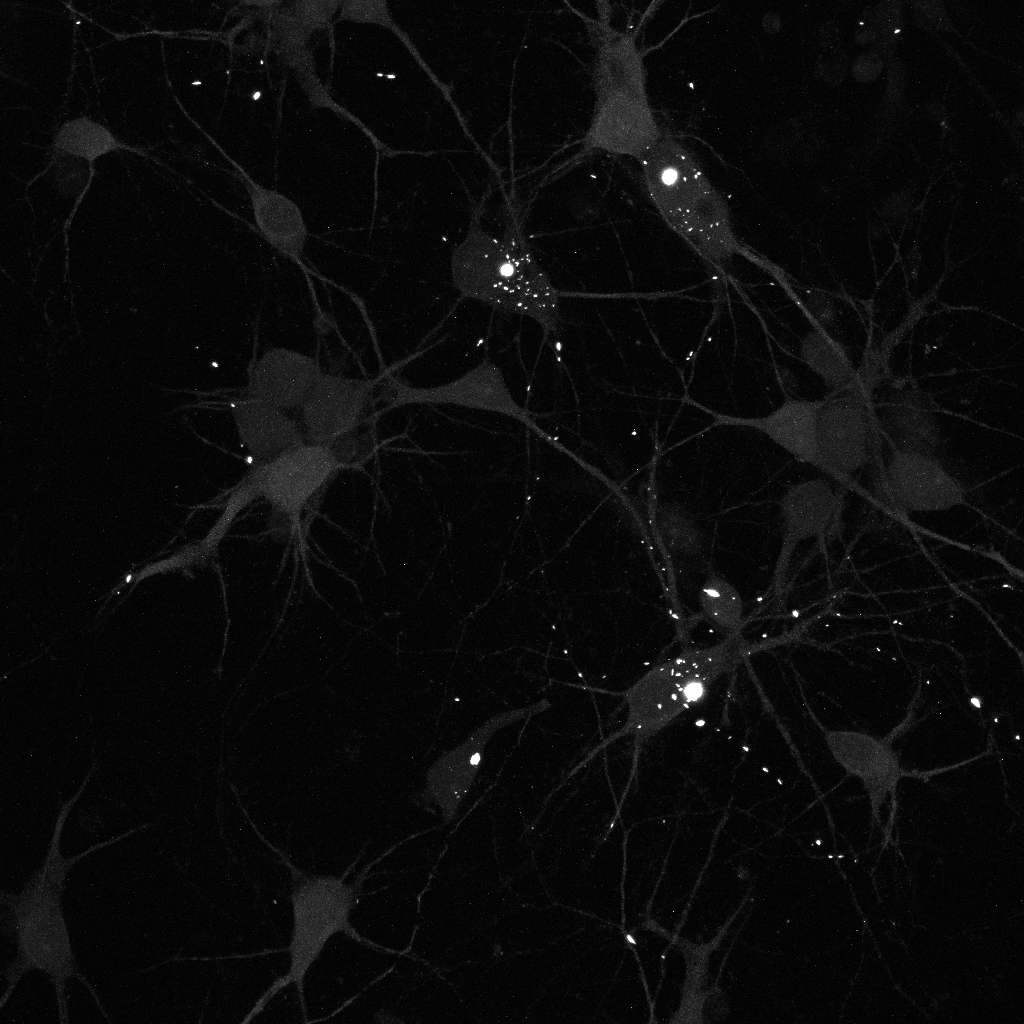

Supplement: Supplementary file 9 — Figure EVs Source Data [file 44319_2024_140_MOESM9_ESM.zip › SD EV figures/Supplementary Figure 3 - EV3/Panel S3C/split images/C2-MAX_RANT_ISRIB_2DG001.tif]

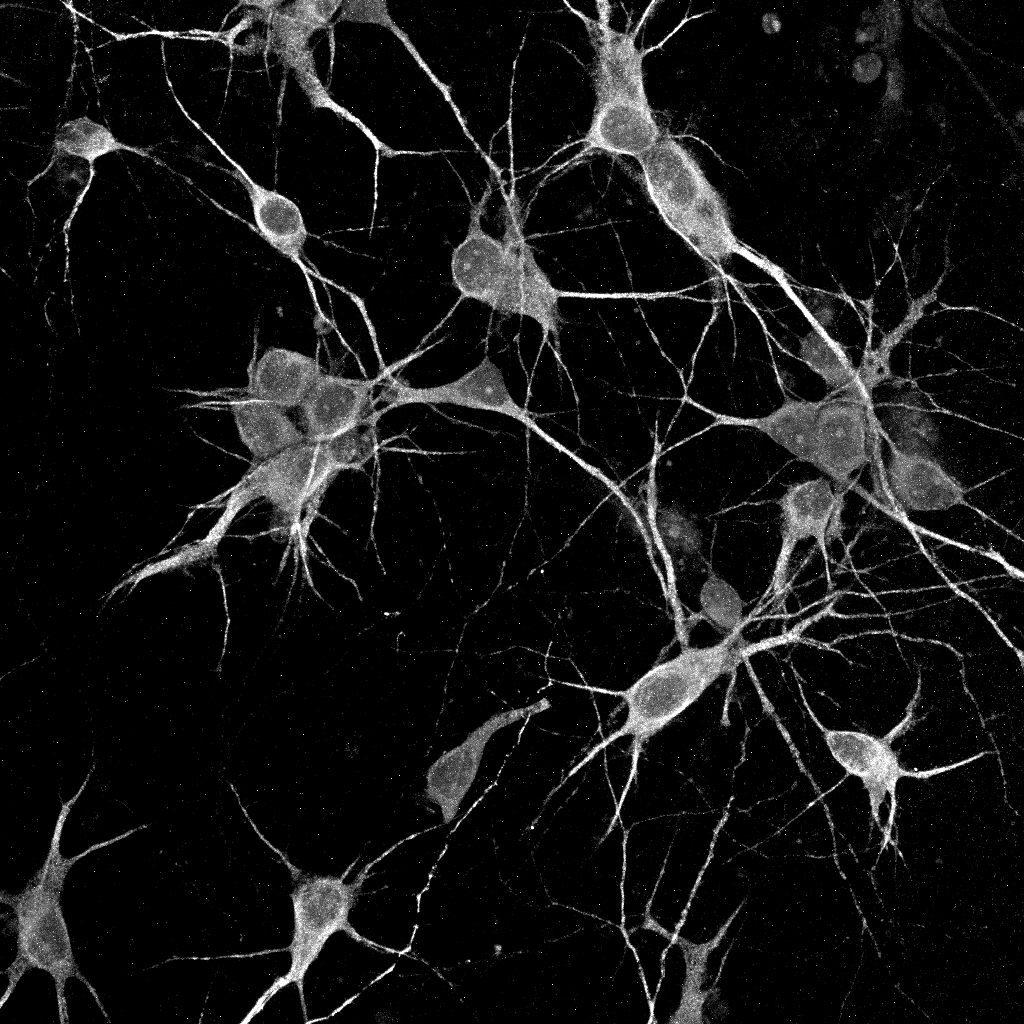

Supplement: Supplementary file 9 — Figure EVs Source Data [file 44319_2024_140_MOESM9_ESM.zip › SD EV figures/Supplementary Figure 3 - EV3/Panel S3C/split images/merge 2DG.tif]

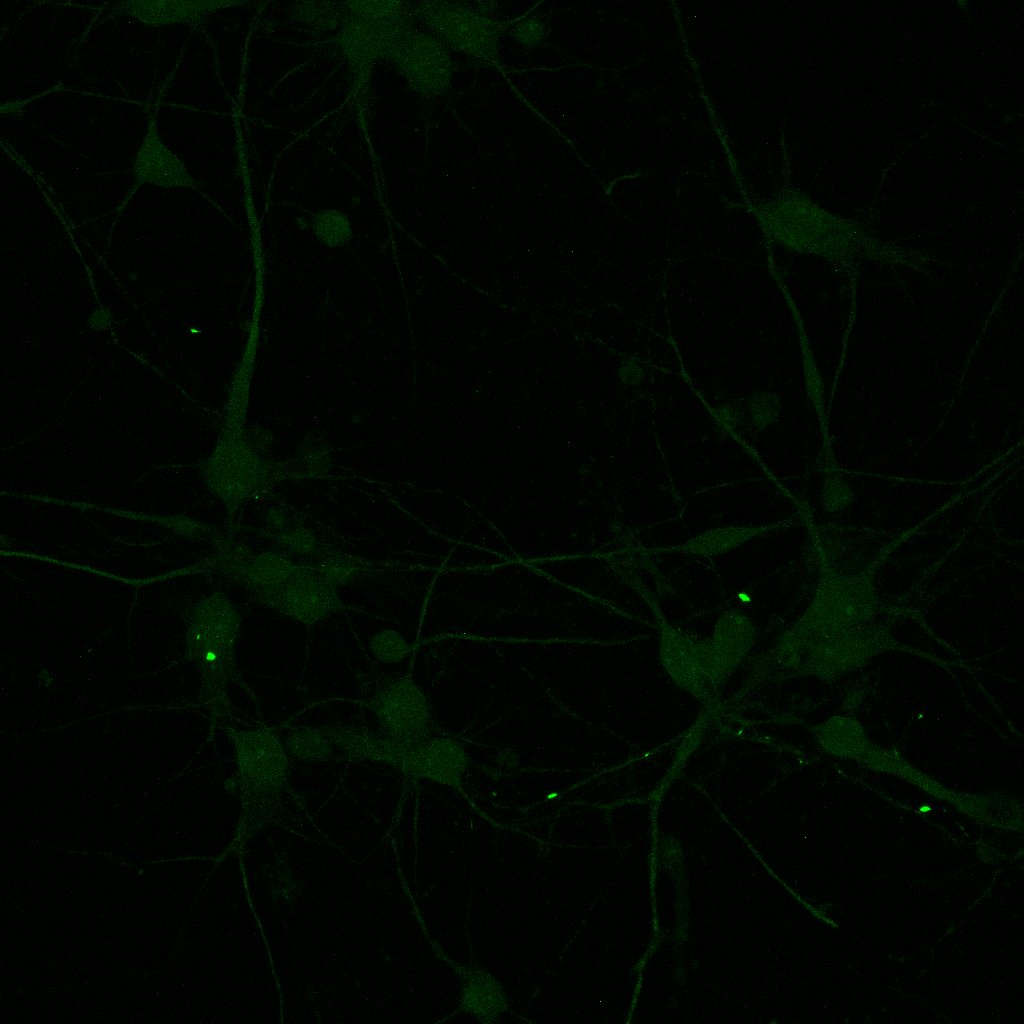

Supplement: Supplementary file 9 — Figure EVs Source Data [file 44319_2024_140_MOESM9_ESM.zip › SD EV figures/Supplementary Figure 3 - EV3/Panel S3C/split images/C2-MAX_RANT_ISRIB_2DG+ISRIB.jpg]

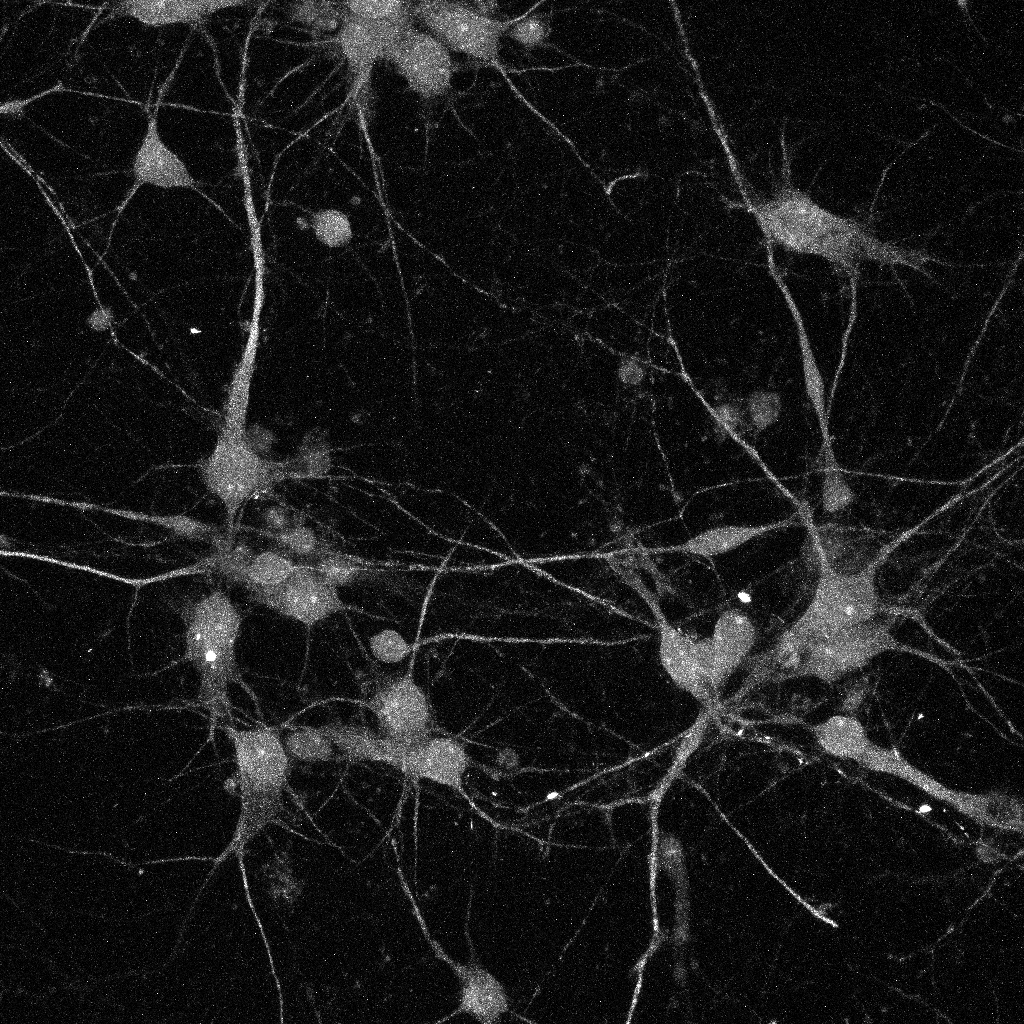

Supplement: Supplementary file 9 — Figure EVs Source Data [file 44319_2024_140_MOESM9_ESM.zip › SD EV figures/Supplementary Figure 3 - EV3/Panel S3C/split images/C2-MAX_RANT_ISRIB_2DG+ISRIB.tif]

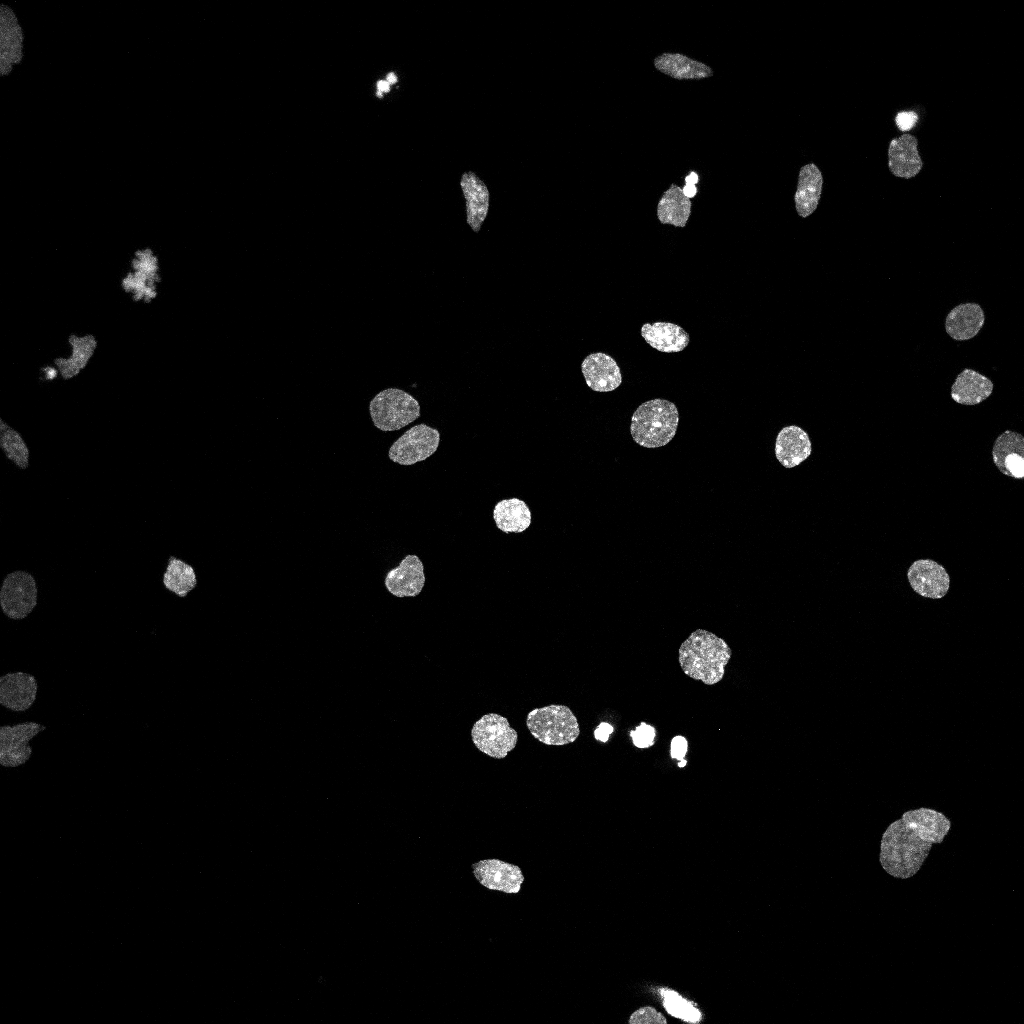

Supplement: Supplementary file 9 — Figure EVs Source Data [file 44319_2024_140_MOESM9_ESM.zip › SD EV figures/Supplementary Figure 3 - EV3/Panel S3C/split images/C1-MAX_RANT_ISRIB_compl.tif]

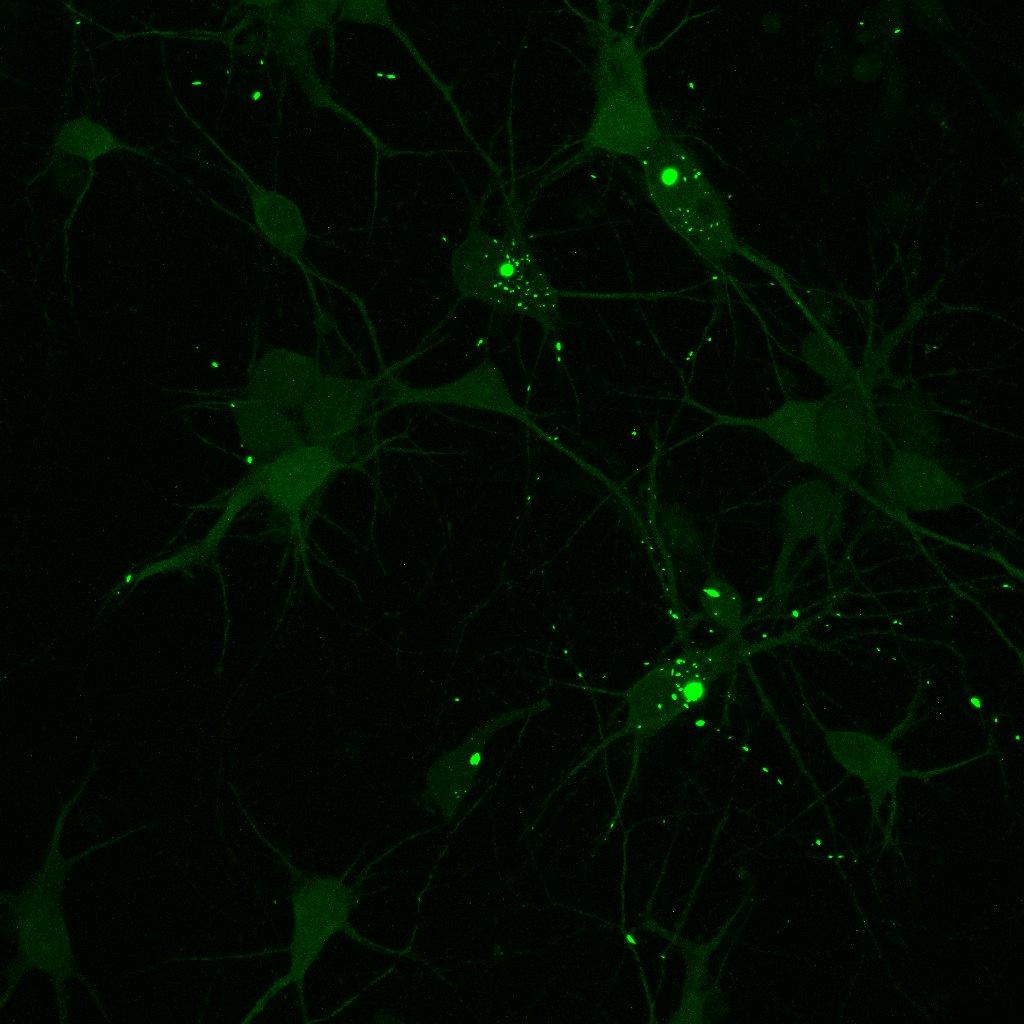

Supplement: Supplementary file 9 — Figure EVs Source Data [file 44319_2024_140_MOESM9_ESM.zip › SD EV figures/Supplementary Figure 3 - EV3/Panel S3C/split images/C2-MAX_RANT_ISRIB_2DG001.jpg]

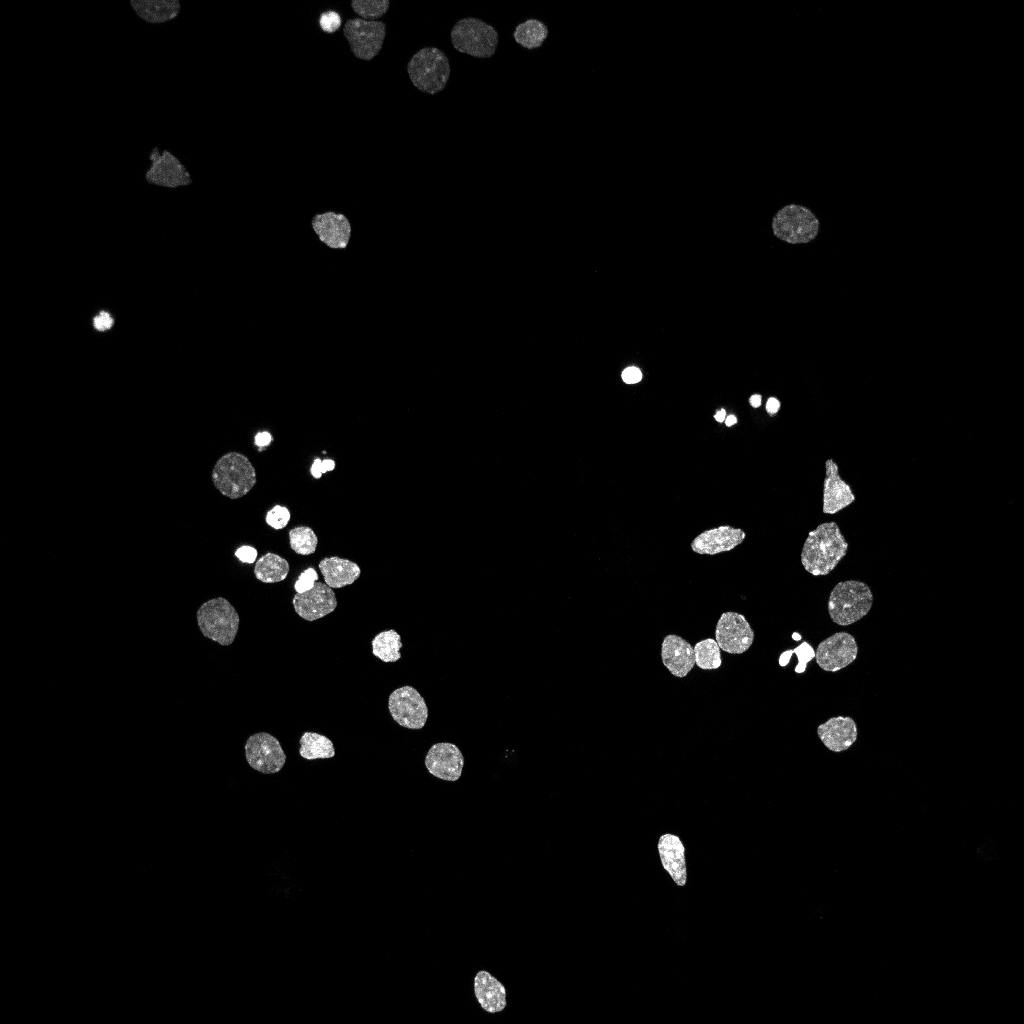

Supplement: Supplementary file 9 — Figure EVs Source Data [file 44319_2024_140_MOESM9_ESM.zip › SD EV figures/Supplementary Figure 3 - EV3/Panel S3C/split images/C1-MAX_RANT_ISRIB_2DG+ISRIB.tif]

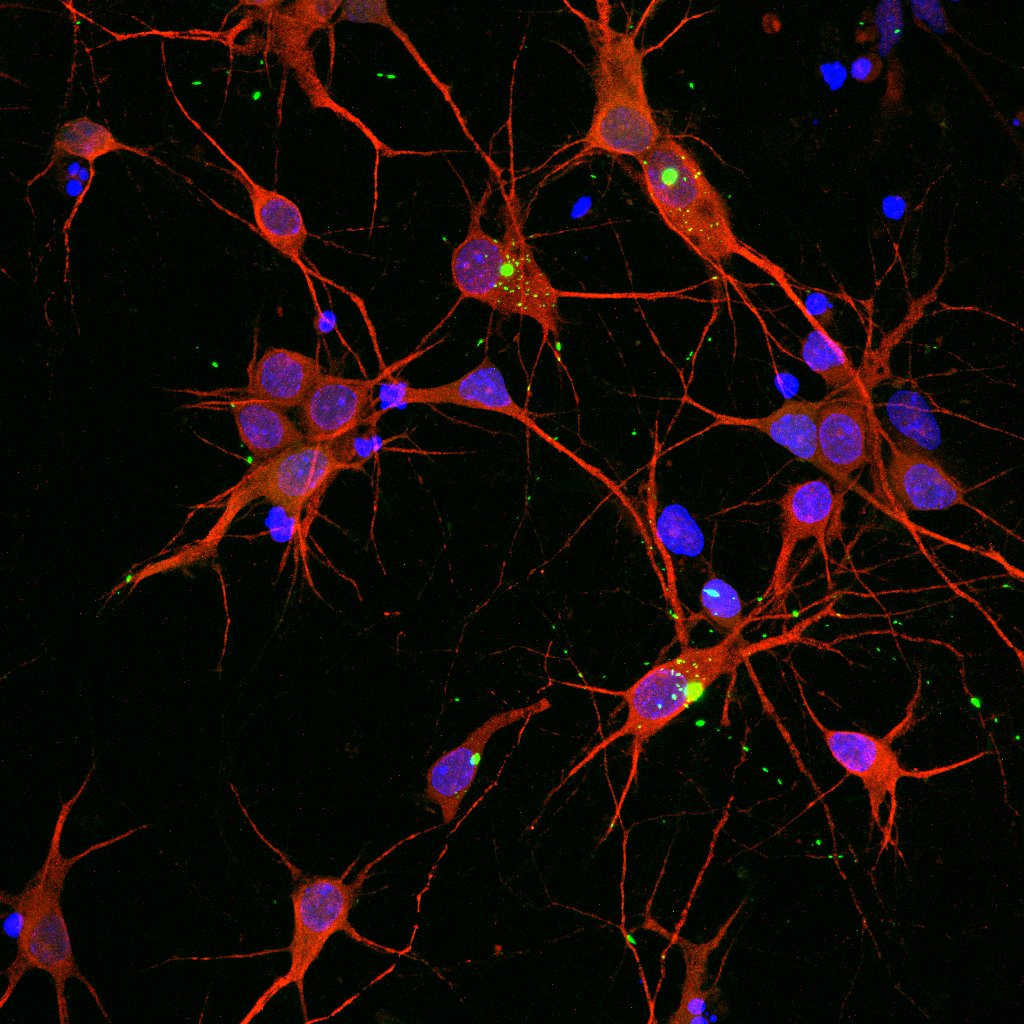

Supplement: Supplementary file 9 — Figure EVs Source Data [file 44319_2024_140_MOESM9_ESM.zip › SD EV figures/Supplementary Figure 3 - EV3/Panel S3C/split images/merge 2DG.jpg]

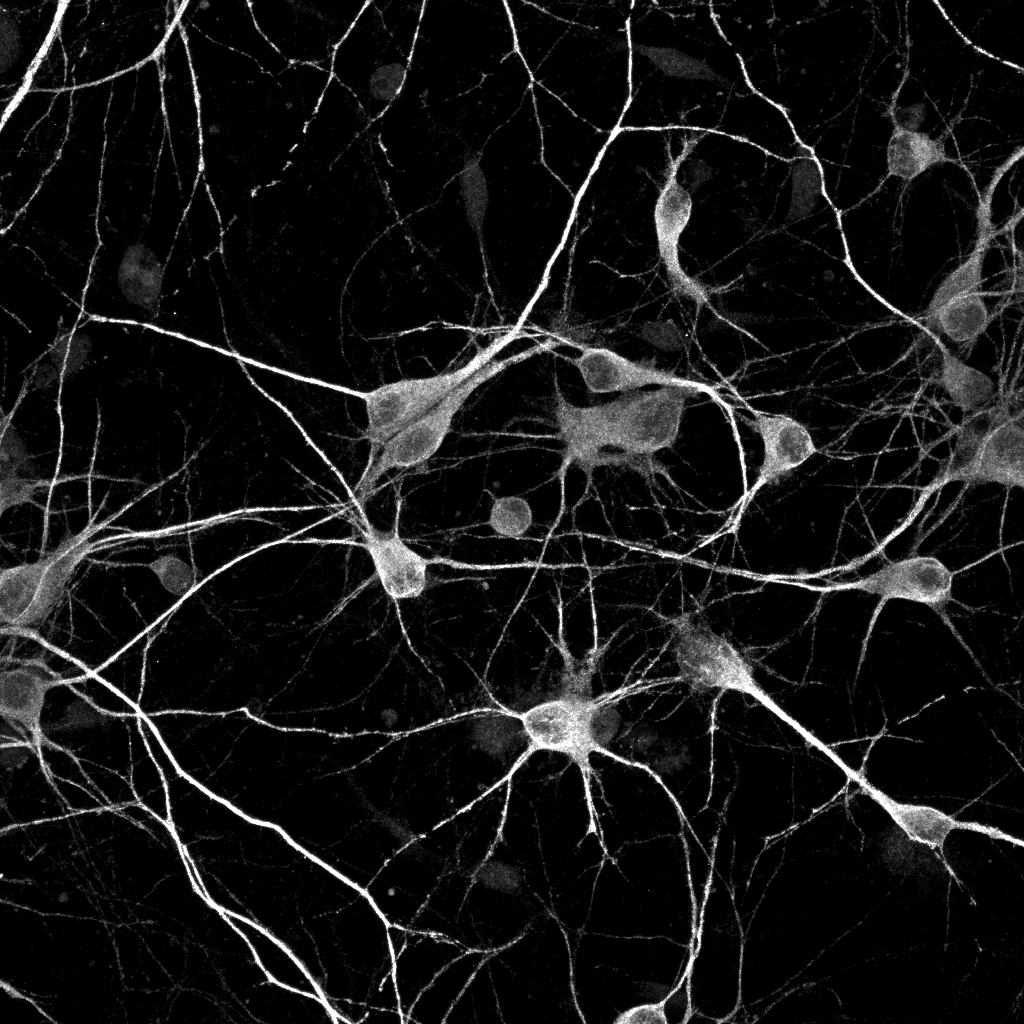

Supplement: Supplementary file 9 — Figure EVs Source Data [file 44319_2024_140_MOESM9_ESM.zip › SD EV figures/Supplementary Figure 3 - EV3/Panel S3C/split images/C3-MAX_RANT_ISRIB_compl.tif]

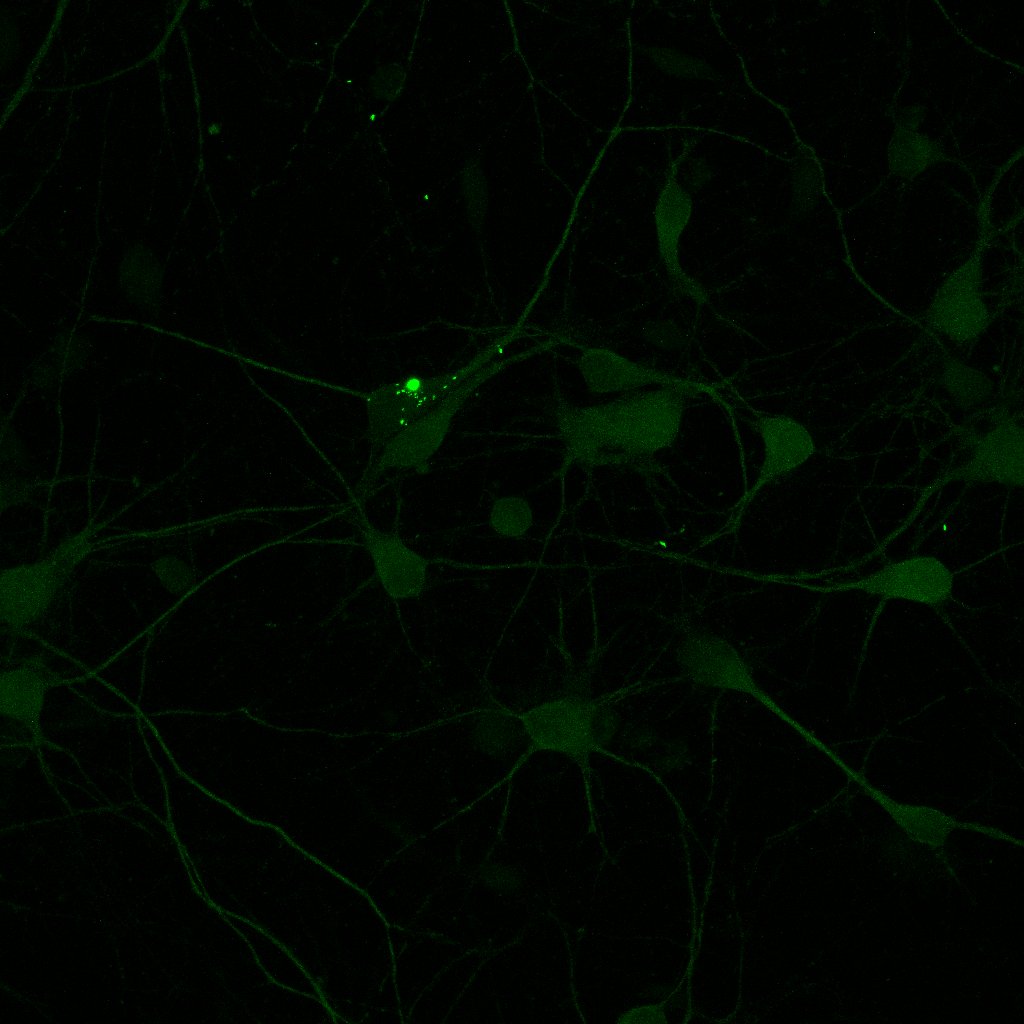

Supplement: Supplementary file 9 — Figure EVs Source Data [file 44319_2024_140_MOESM9_ESM.zip › SD EV figures/Supplementary Figure 3 - EV3/Panel S3C/split images/C2-MAX_RANT_ISRIB_compl.jpg]

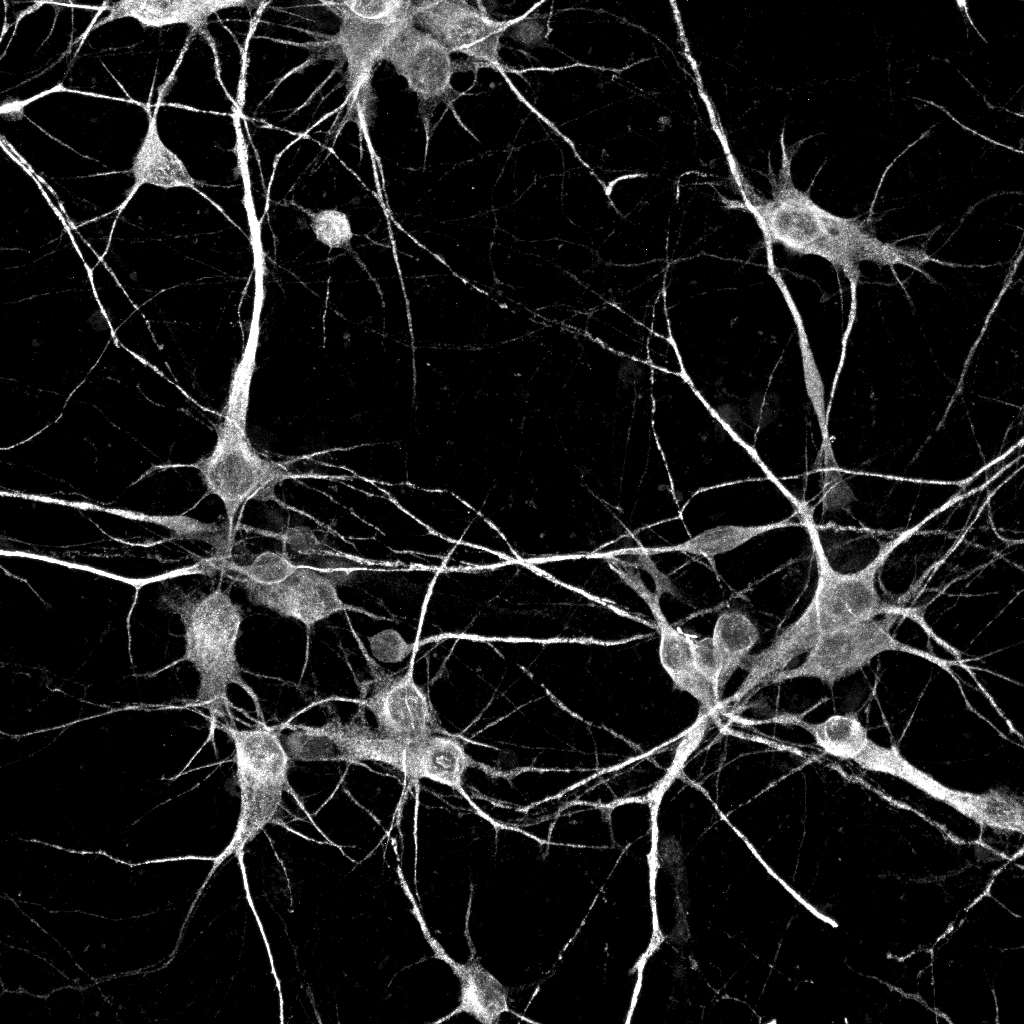

Supplement: Supplementary file 9 — Figure EVs Source Data [file 44319_2024_140_MOESM9_ESM.zip › SD EV figures/Supplementary Figure 3 - EV3/Panel S3C/split images/merge 2DG+ISRIB.tif]

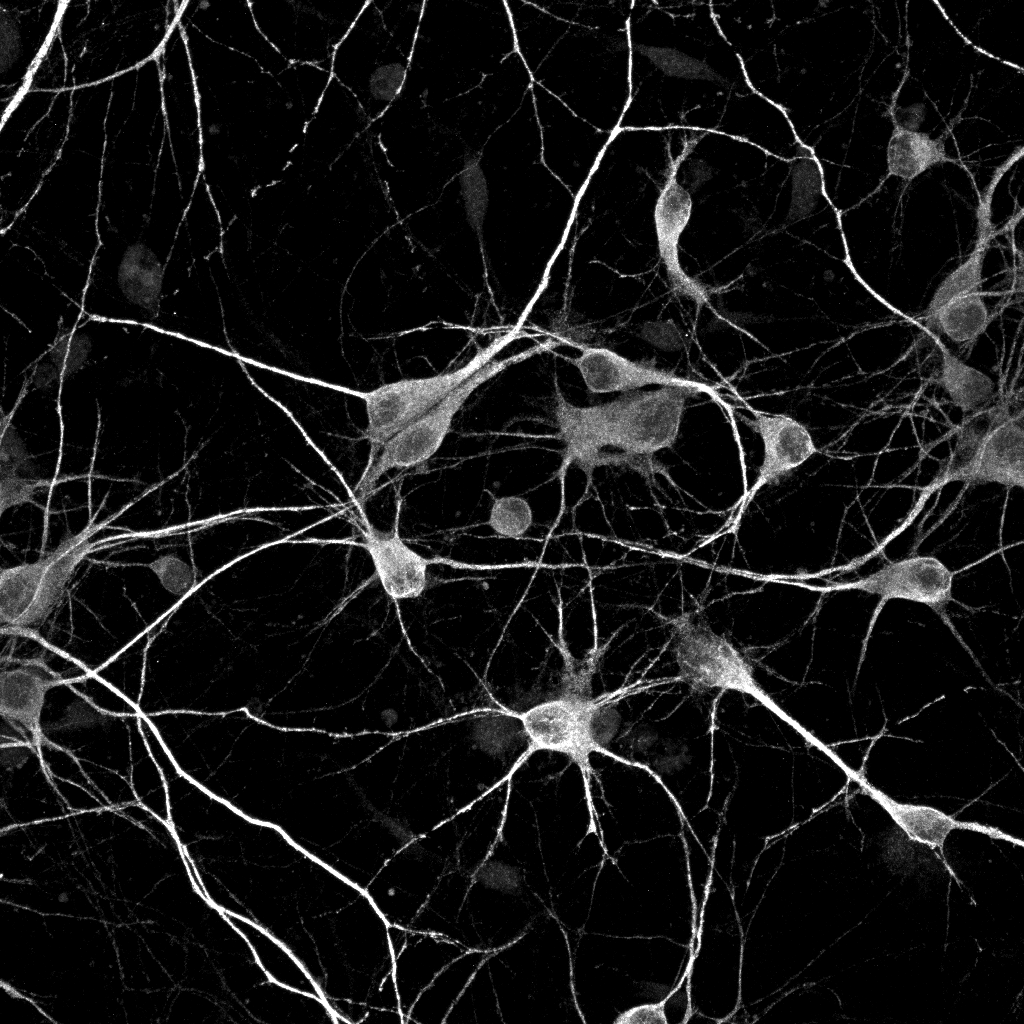

Supplement: Supplementary file 9 — Figure EVs Source Data [file 44319_2024_140_MOESM9_ESM.zip › SD EV figures/Supplementary Figure 3 - EV3/Panel S3C/split images/merge complete.tif]

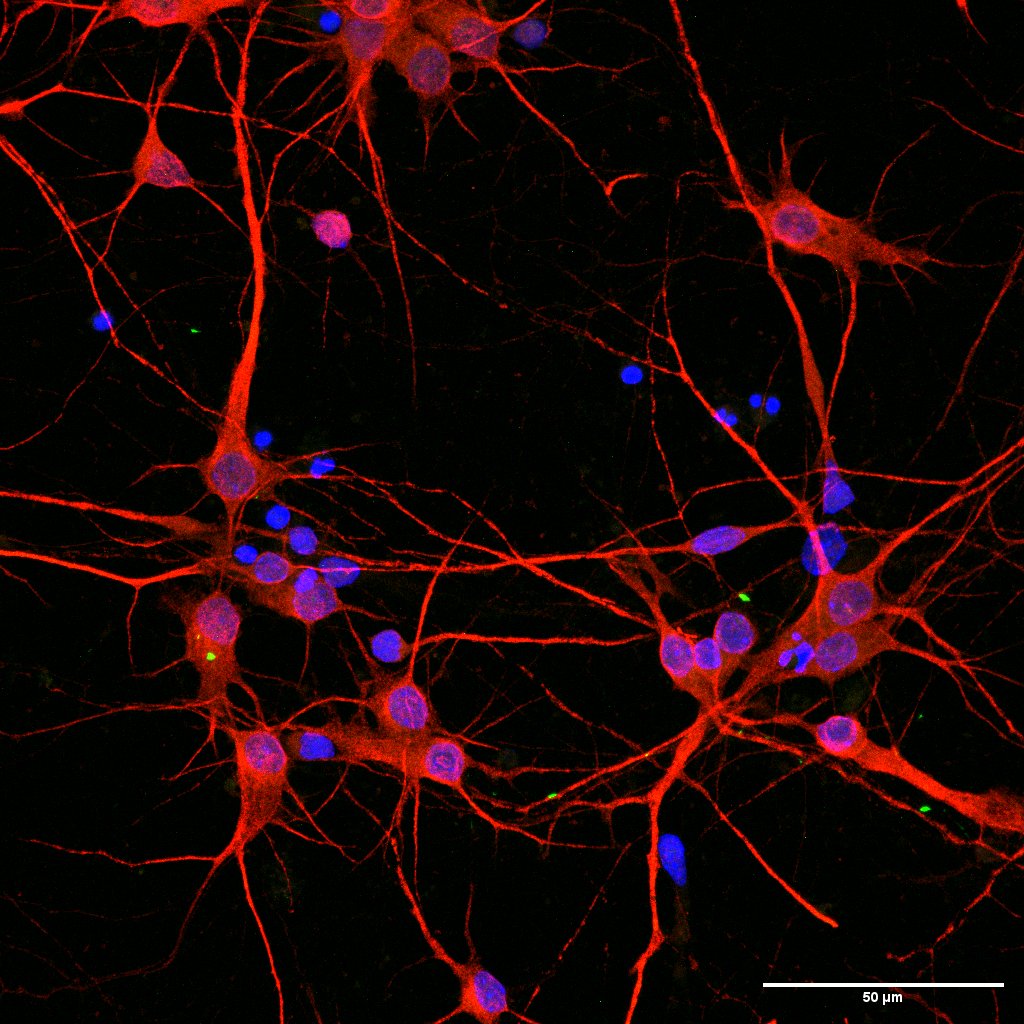

Supplement: Supplementary file 9 — Figure EVs Source Data [file 44319_2024_140_MOESM9_ESM.zip › SD EV figures/Supplementary Figure 3 - EV3/Panel S3C/split images/merge 2DG+ISRIB_SB.jpg]

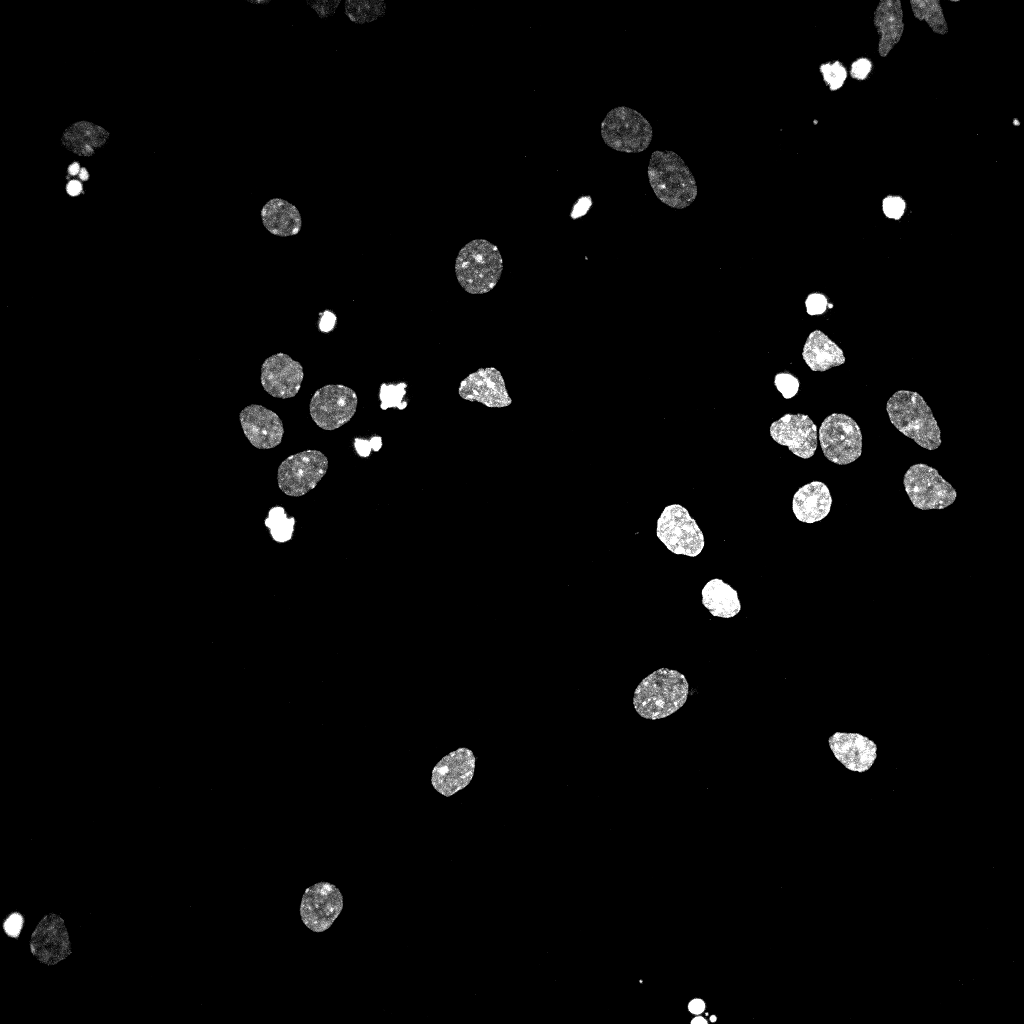

Supplement: Supplementary file 9 — Figure EVs Source Data [file 44319_2024_140_MOESM9_ESM.zip › SD EV figures/Supplementary Figure 3 - EV3/Panel S3C/split images/C1-MAX_RANT_ISRIB_2DG001.tif]

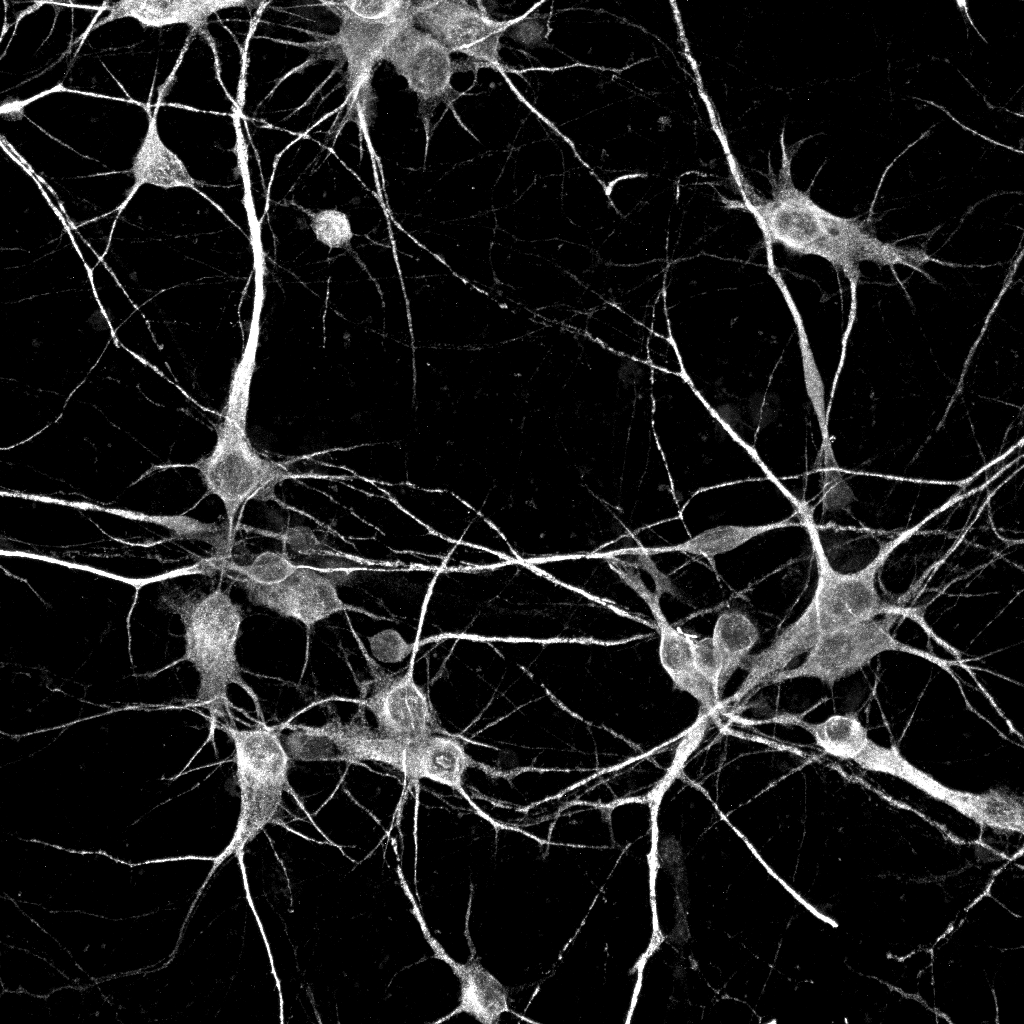

Supplement: Supplementary file 9 — Figure EVs Source Data [file 44319_2024_140_MOESM9_ESM.zip › SD EV figures/Supplementary Figure 3 - EV3/Panel S3C/split images/C3-MAX_RANT_ISRIB_2DG+ISRIB.tif]

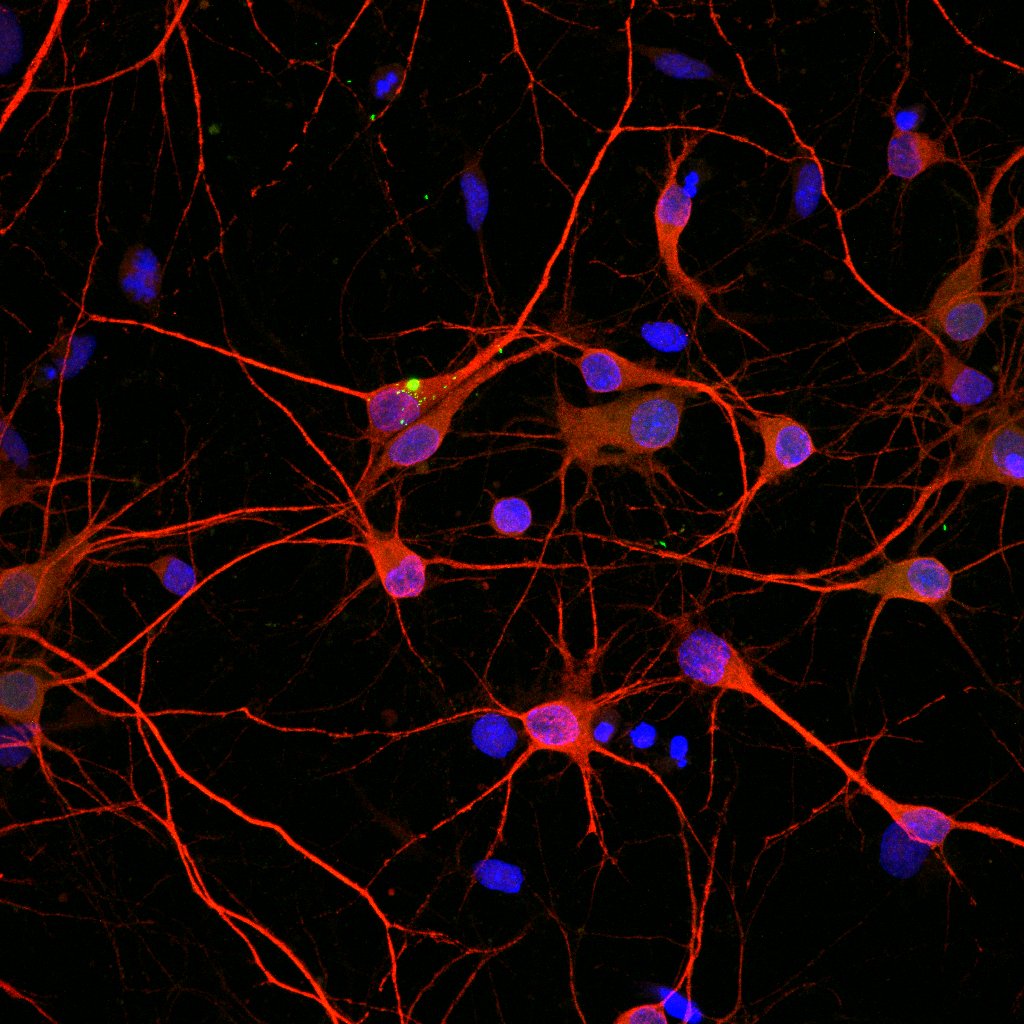

Supplement: Supplementary file 9 — Figure EVs Source Data [file 44319_2024_140_MOESM9_ESM.zip › SD EV figures/Supplementary Figure 3 - EV3/Panel S3C/split images/merge complete.jpg]

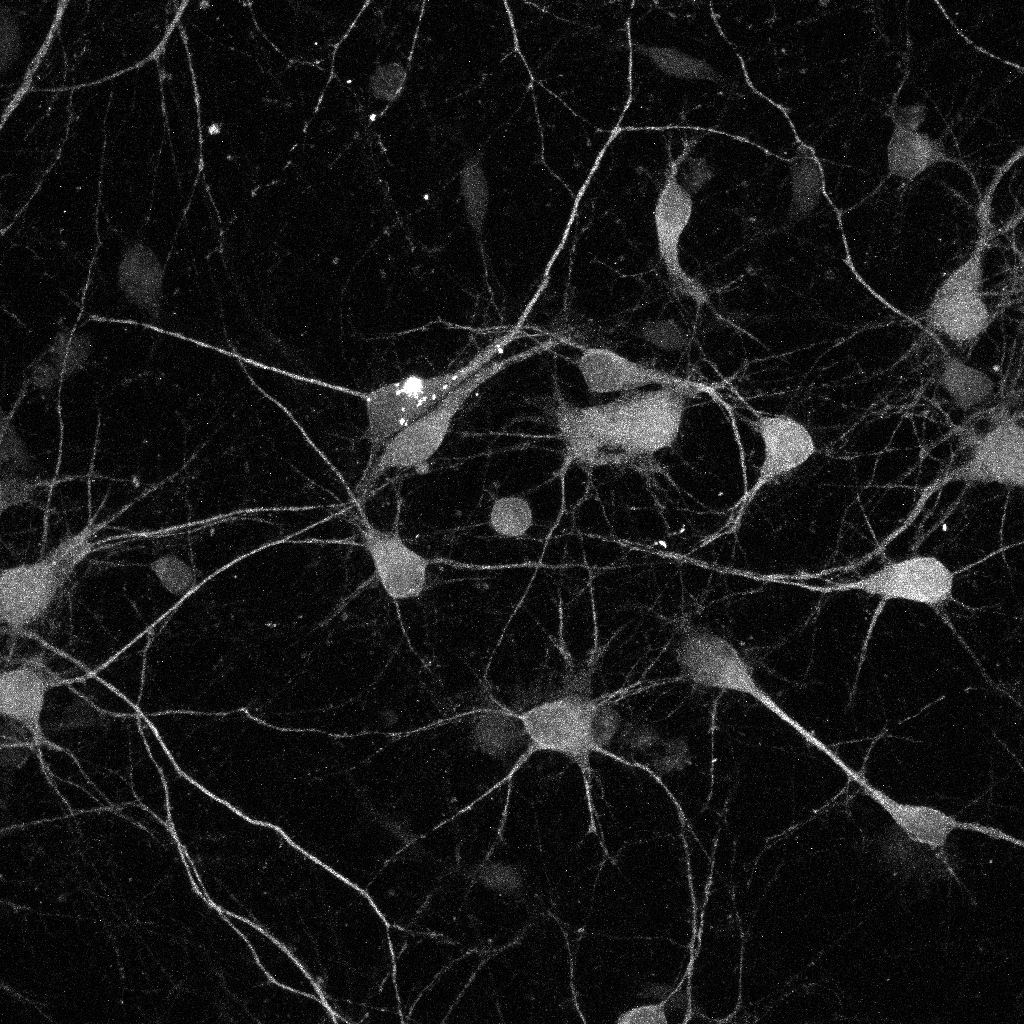

Supplement: Supplementary file 9 — Figure EVs Source Data [file 44319_2024_140_MOESM9_ESM.zip › SD EV figures/Supplementary Figure 3 - EV3/Panel S3C/split images/C2-MAX_RANT_ISRIB_compl.tif]

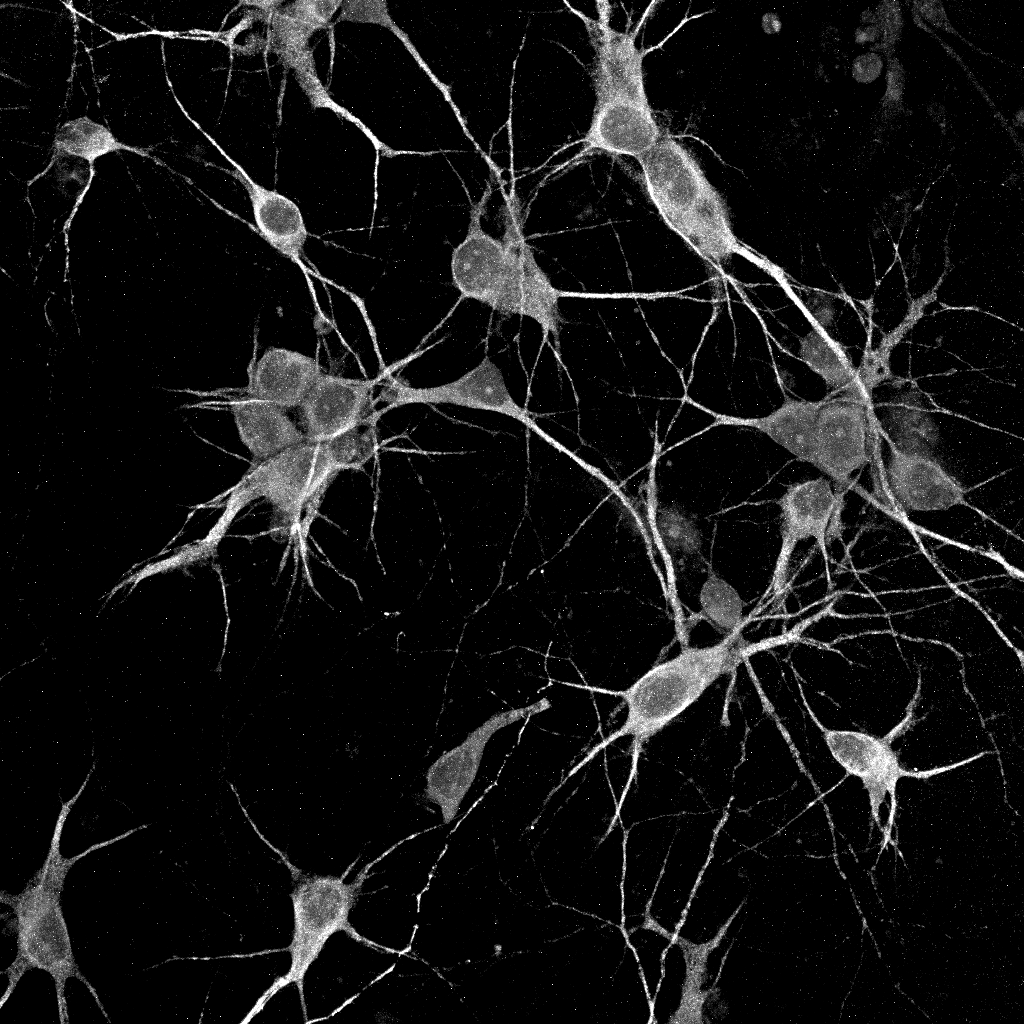

Supplement: Supplementary file 9 — Figure EVs Source Data [file 44319_2024_140_MOESM9_ESM.zip › SD EV figures/Supplementary Figure 3 - EV3/Panel S3C/split images/C3-MAX_RANT_ISRIB_2DG001.tif]

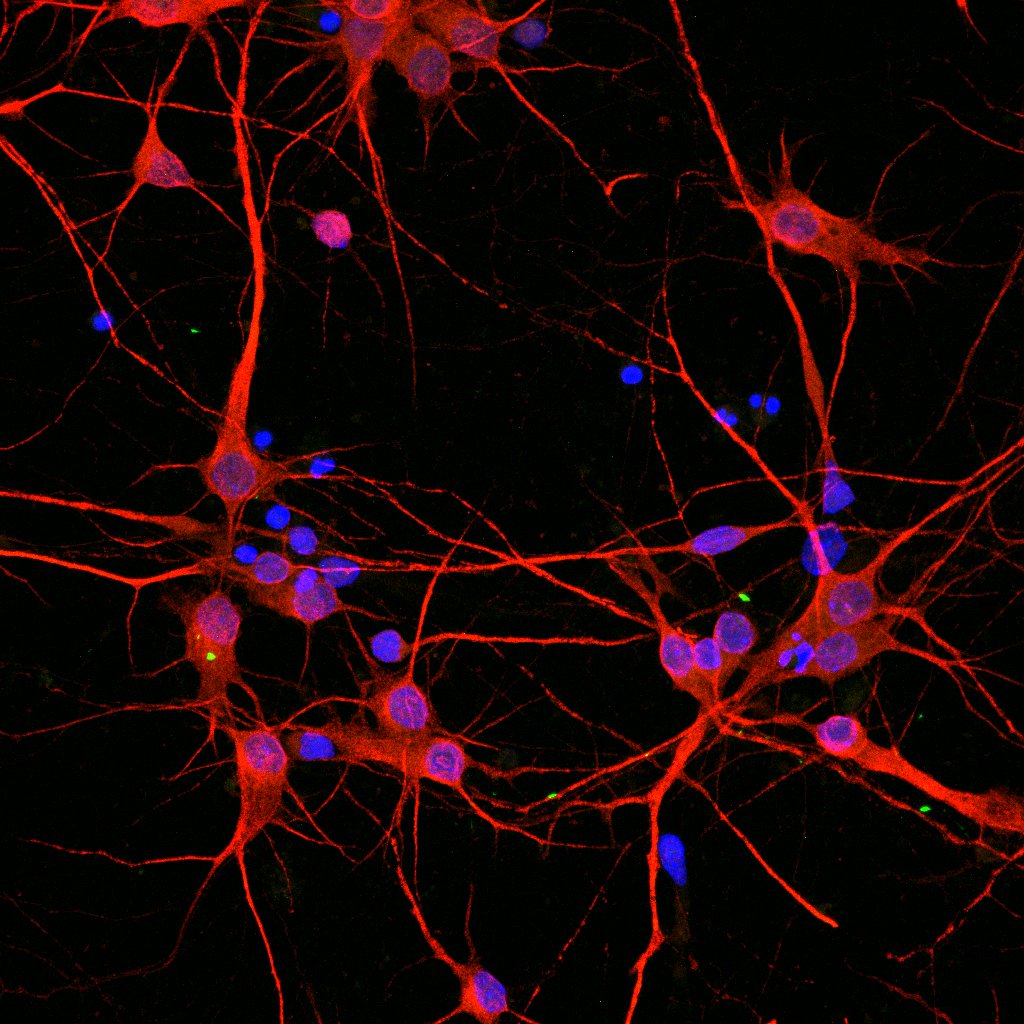

Supplement: Supplementary file 9 — Figure EVs Source Data [file 44319_2024_140_MOESM9_ESM.zip › SD EV figures/Supplementary Figure 3 - EV3/Panel S3C/split images/merge 2DG+ISRIB.jpg]

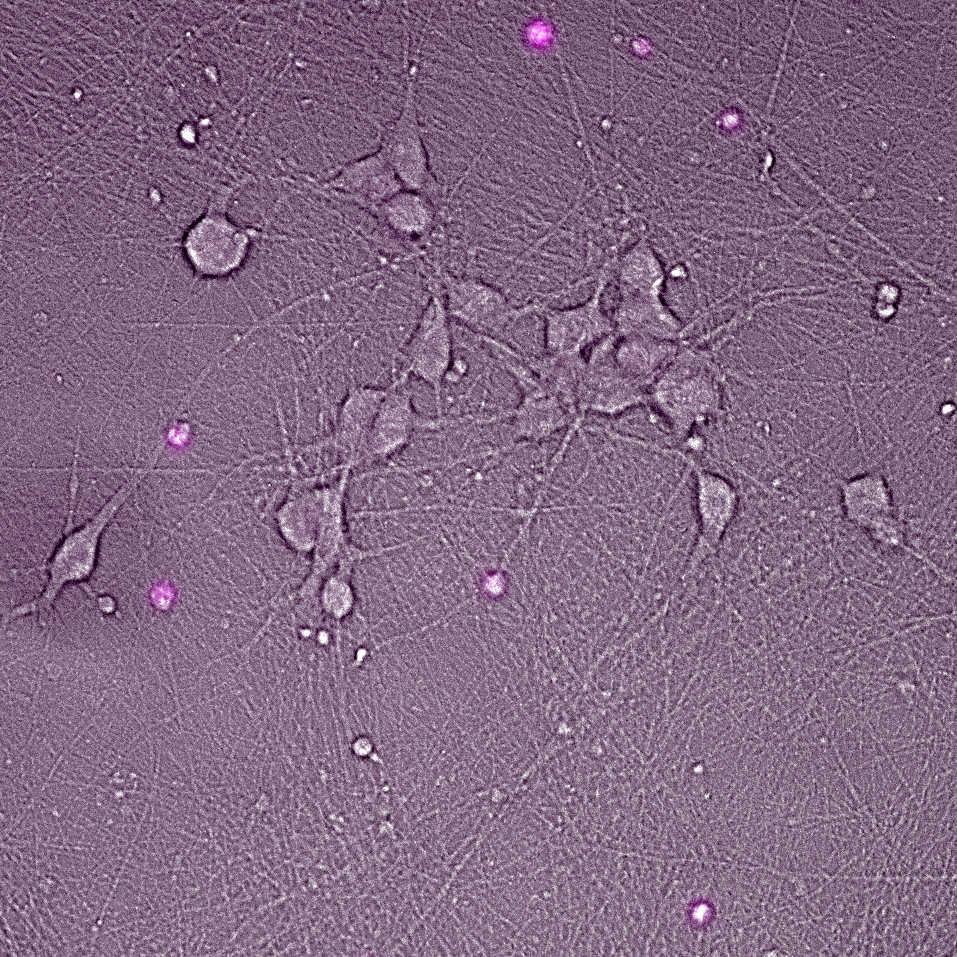

Supplement: Supplementary file 9 — Figure EVs Source Data [file 44319_2024_140_MOESM9_ESM.zip › SD EV figures/Supplementary Figure 3 - EV3/Panel S3B/representative images/G3_DMSO_48h.tif]

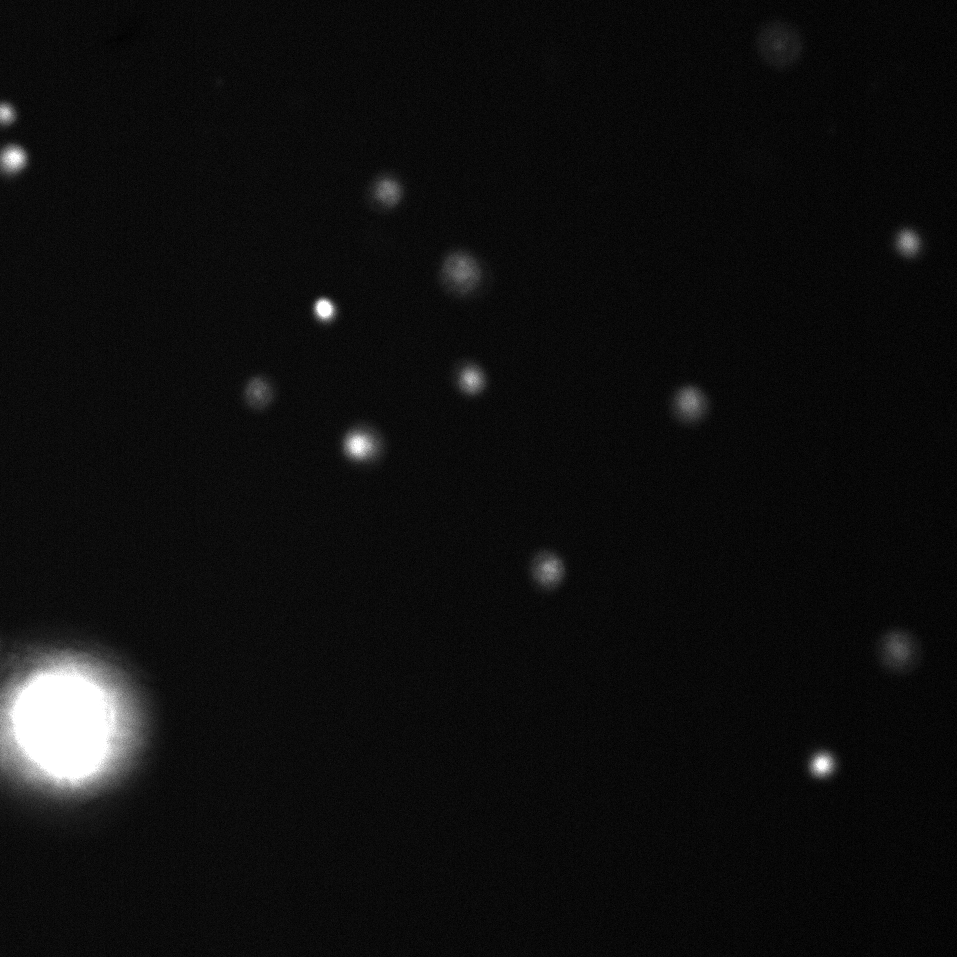

Supplement: Supplementary file 9 — Figure EVs Source Data [file 44319_2024_140_MOESM9_ESM.zip › SD EV figures/Supplementary Figure 3 - EV3/Panel S3B/representative images/scale bar.tif]

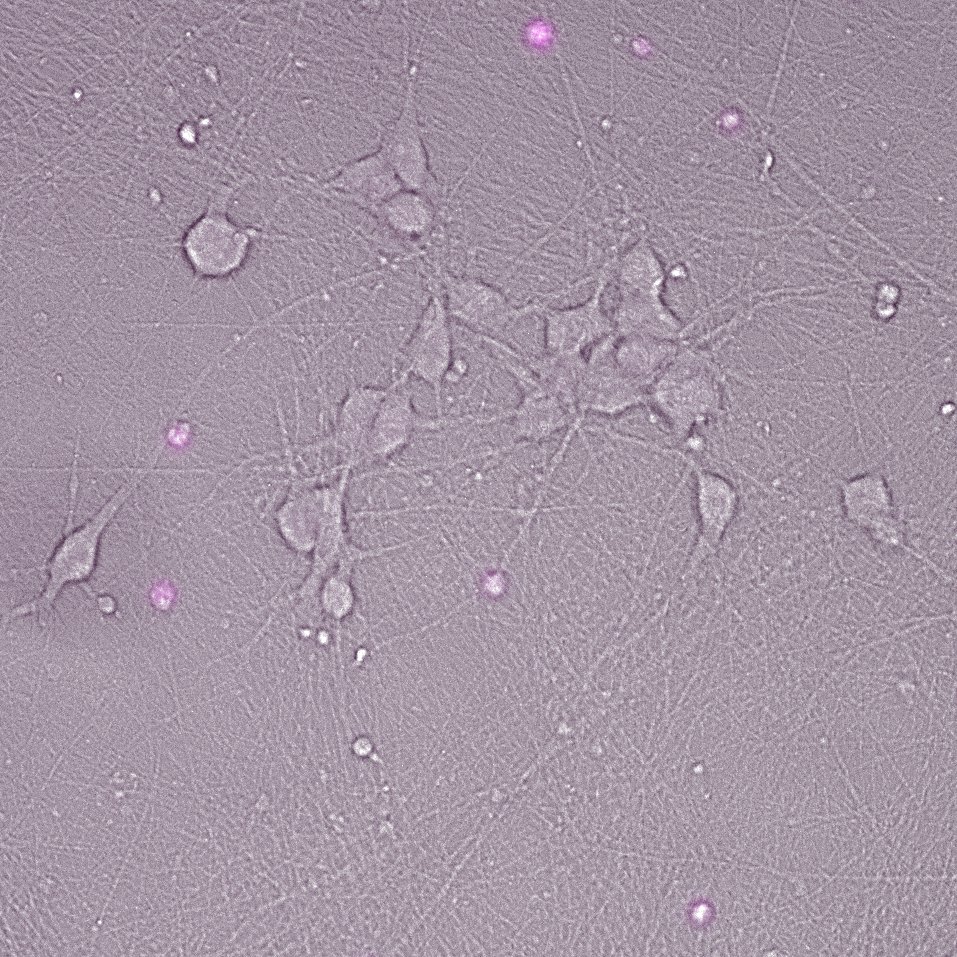

Supplement: Supplementary file 9 — Figure EVs Source Data [file 44319_2024_140_MOESM9_ESM.zip › SD EV figures/Supplementary Figure 3 - EV3/Panel S3B/representative images/G3_DMSO_48h.jpg]

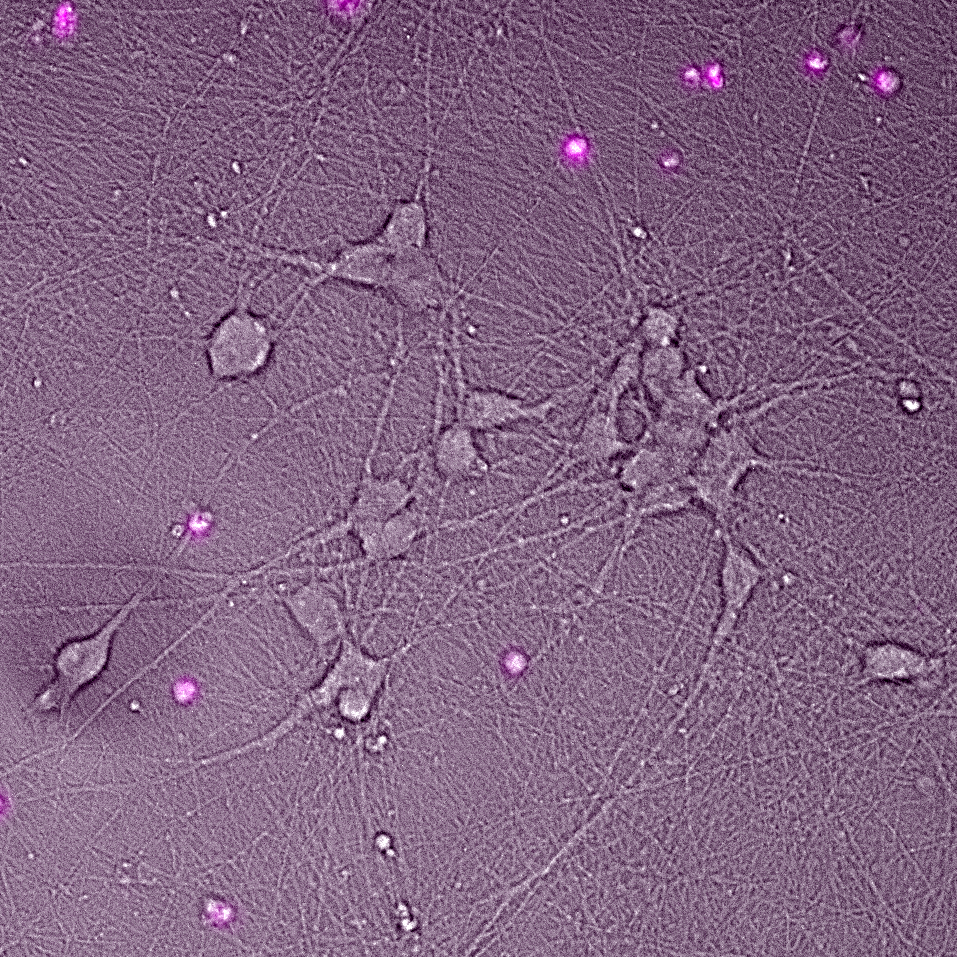

Supplement: Supplementary file 9 — Figure EVs Source Data [file 44319_2024_140_MOESM9_ESM.zip › SD EV figures/Supplementary Figure 3 - EV3/Panel S3B/representative images/G3_DMSO_0h.tif]

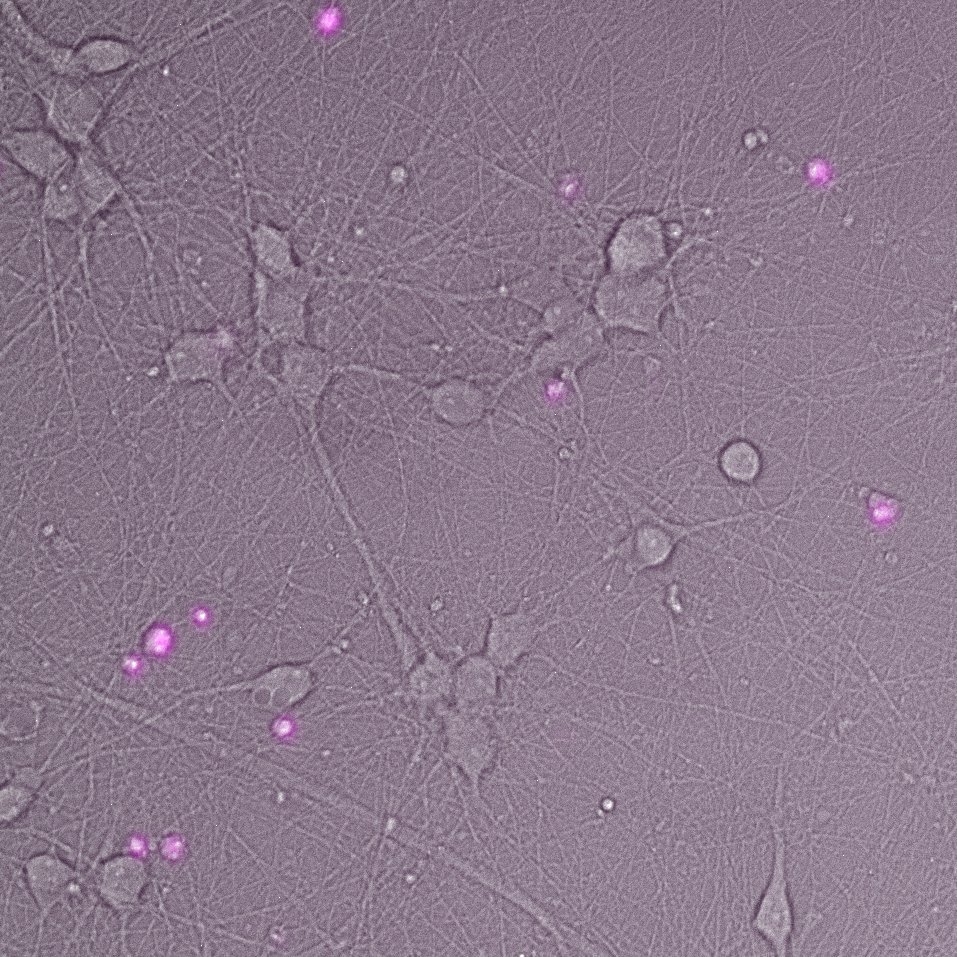

Supplement: Supplementary file 9 — Figure EVs Source Data [file 44319_2024_140_MOESM9_ESM.zip › SD EV figures/Supplementary Figure 3 - EV3/Panel S3B/representative images/G3_A92_0h.jpg]

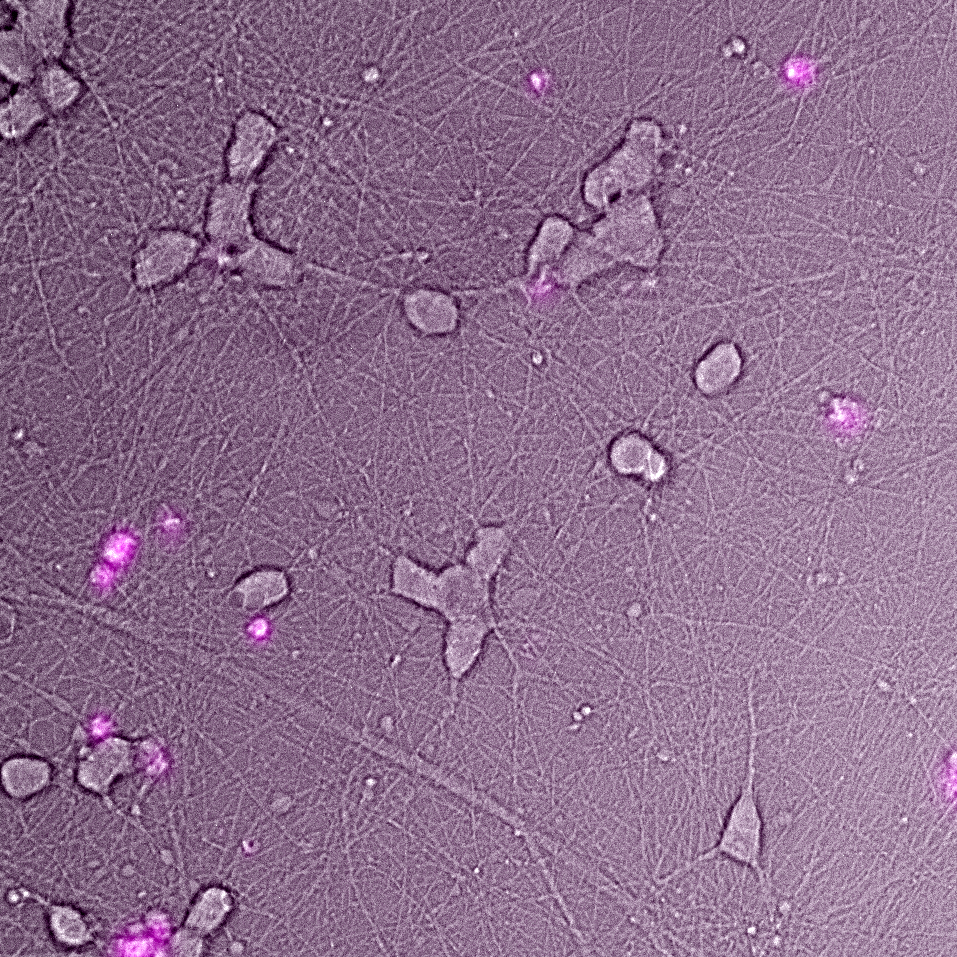

Supplement: Supplementary file 9 — Figure EVs Source Data [file 44319_2024_140_MOESM9_ESM.zip › SD EV figures/Supplementary Figure 3 - EV3/Panel S3B/representative images/G3_A92_48h.tif]

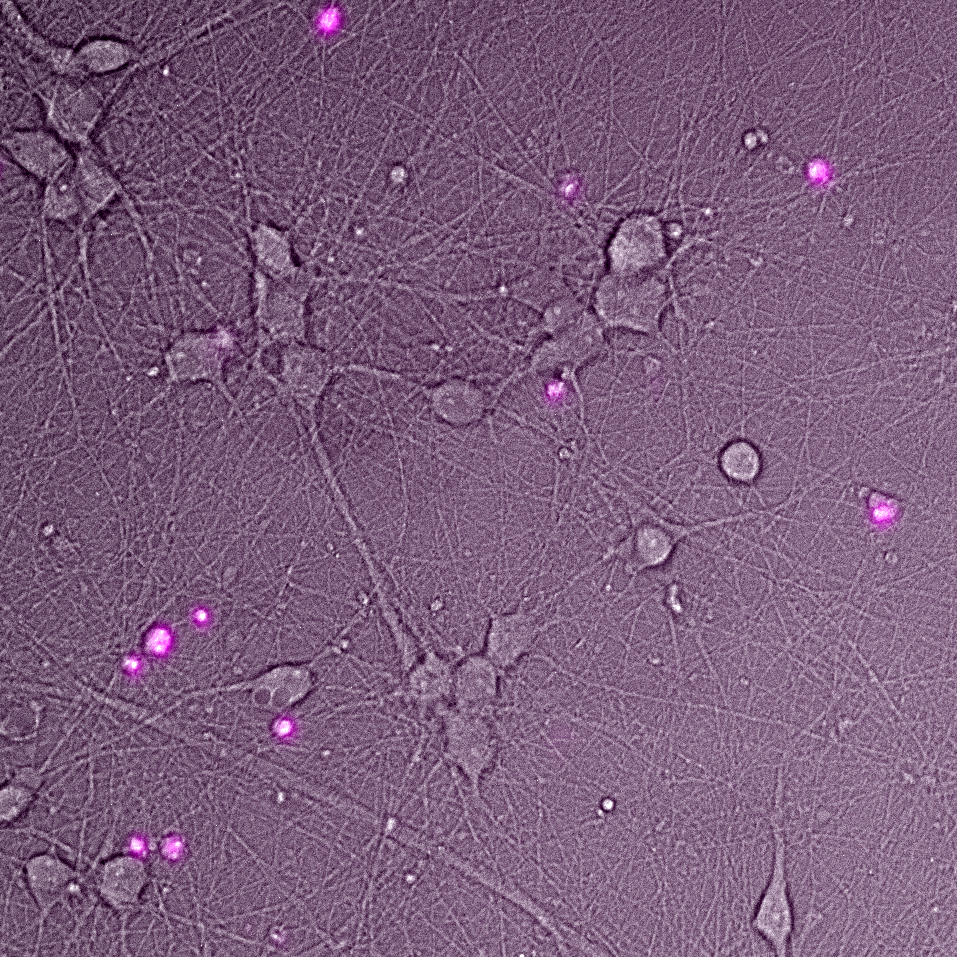

Supplement: Supplementary file 9 — Figure EVs Source Data [file 44319_2024_140_MOESM9_ESM.zip › SD EV figures/Supplementary Figure 3 - EV3/Panel S3B/representative images/G3_A92_0h.tif]

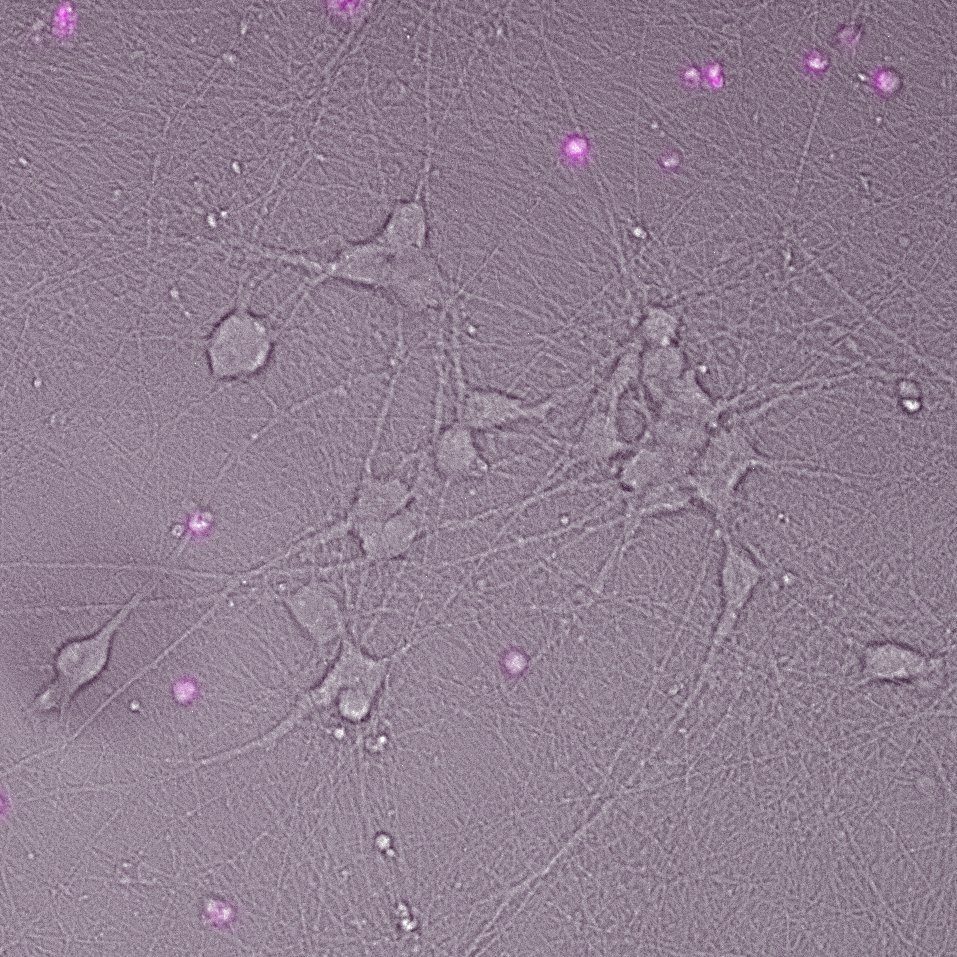

Supplement: Supplementary file 9 — Figure EVs Source Data [file 44319_2024_140_MOESM9_ESM.zip › SD EV figures/Supplementary Figure 3 - EV3/Panel S3B/representative images/G3_DMSO_0h.jpg]

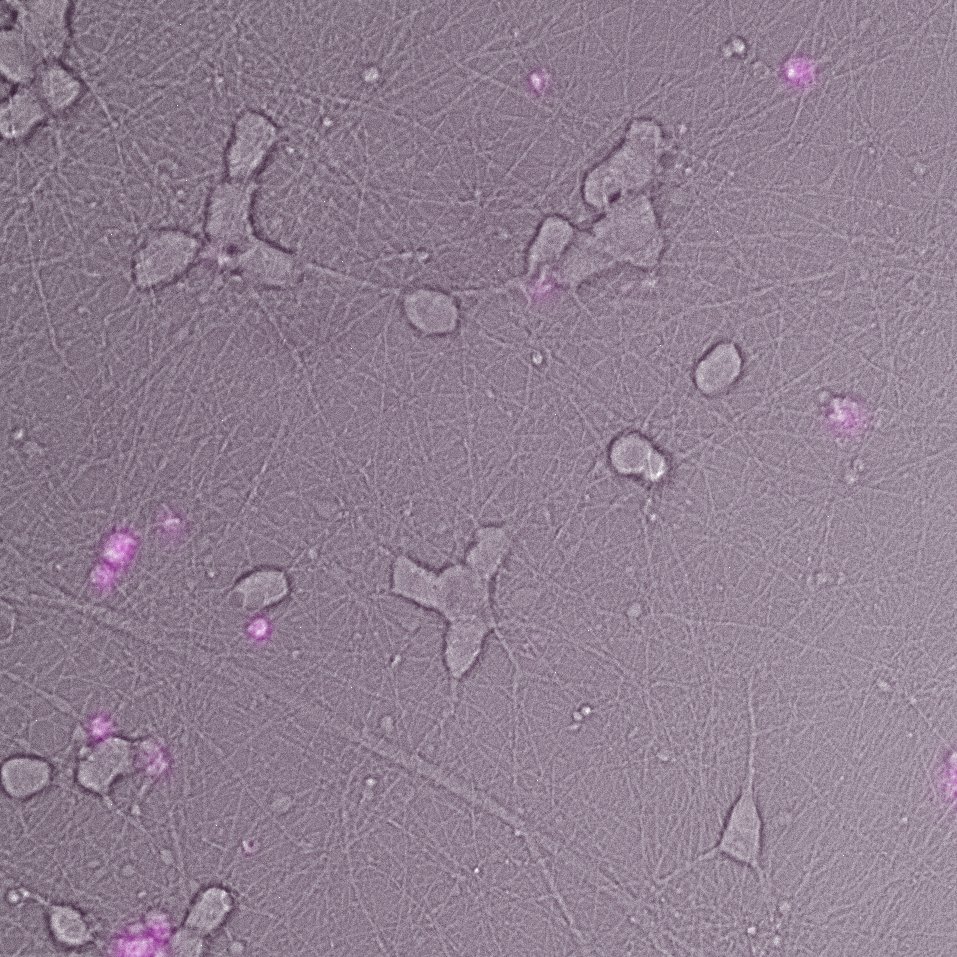

Supplement: Supplementary file 9 — Figure EVs Source Data [file 44319_2024_140_MOESM9_ESM.zip › SD EV figures/Supplementary Figure 3 - EV3/Panel S3B/representative images/G3_A92_48h.jpg]

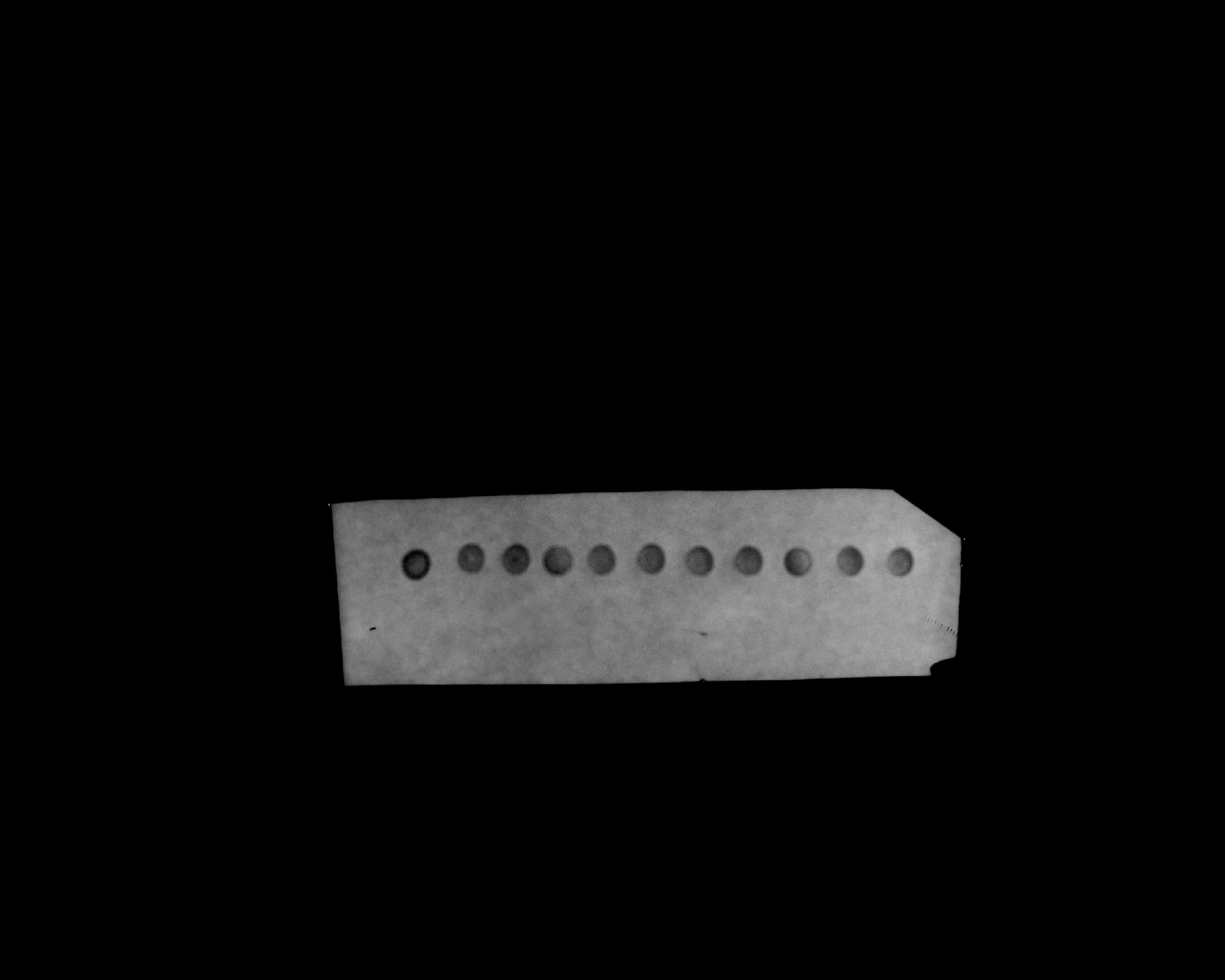

Supplement: Supplementary file 9 — Figure EVs Source Data [file 44319_2024_140_MOESM9_ESM.zip › SD EV figures/Supplementary Figure 6 - EV6/Panel S6E/blot images/full-sized TIFFs/Admin1 2022-10-18 15h10m44s(Colorimetric).tif]

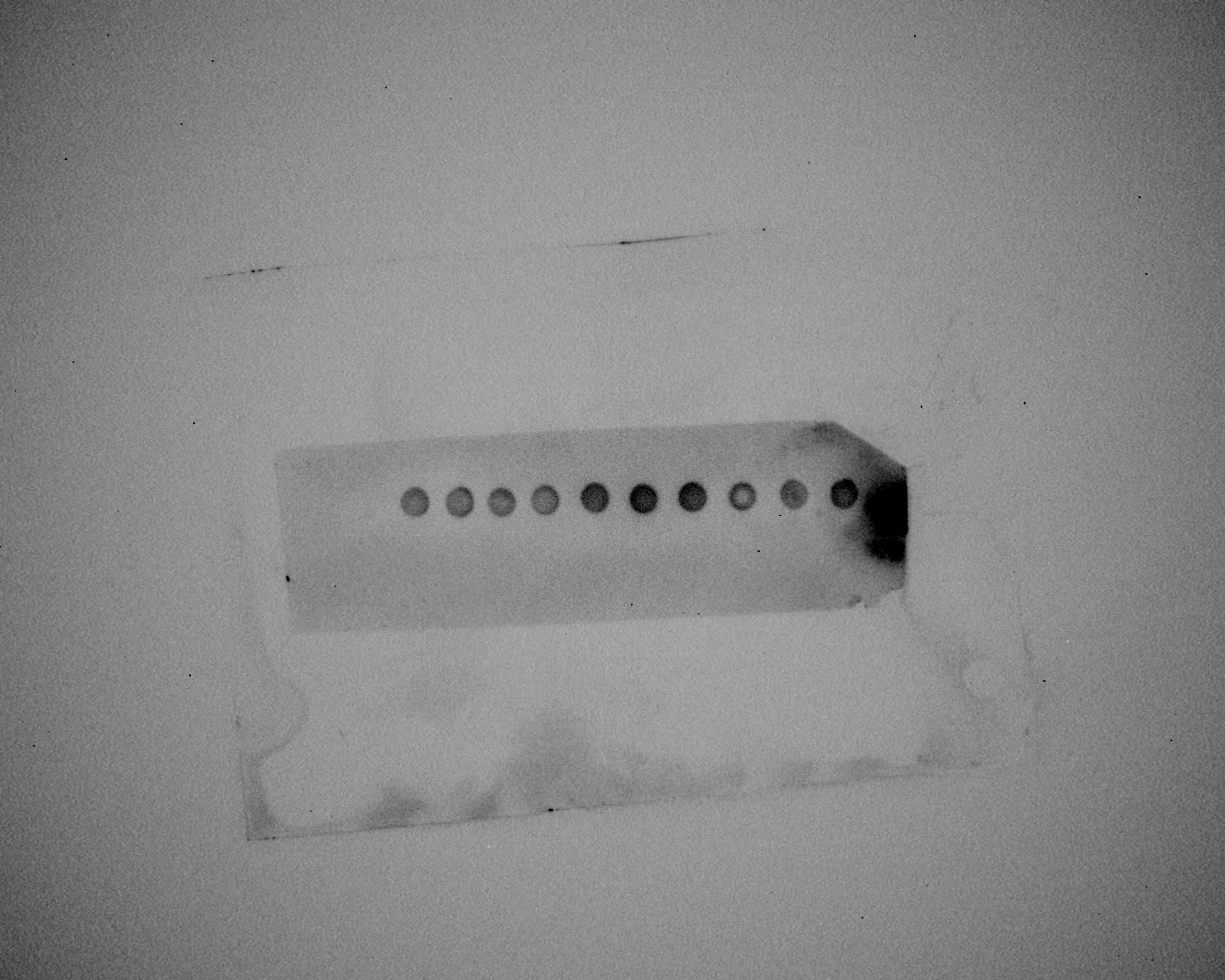

Supplement: Supplementary file 9 — Figure EVs Source Data [file 44319_2024_140_MOESM9_ESM.zip › SD EV figures/Supplementary Figure 6 - EV6/Panel S6E/blot images/full-sized TIFFs/Admin1 2022-10-18 18h26m31s(Chemiluminescence).tif]

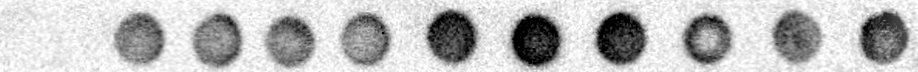

Supplement: Supplementary file 9 — Figure EVs Source Data [file 44319_2024_140_MOESM9_ESM.zip › SD EV figures/Supplementary Figure 6 - EV6/Panel S6E/blot images/cropped JPGs/chemiluminescence (GP).jpg]

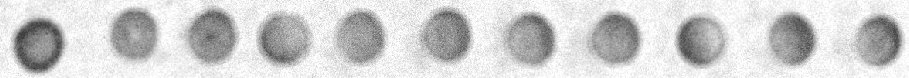

Supplement: Supplementary file 9 — Figure EVs Source Data [file 44319_2024_140_MOESM9_ESM.zip › SD EV figures/Supplementary Figure 6 - EV6/Panel S6E/blot images/cropped JPGs/colorimetric (total protein).jpg]

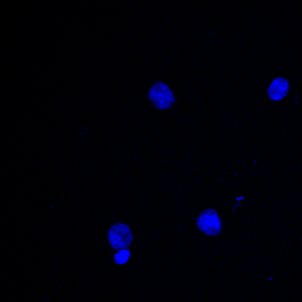

Supplement: Supplementary file 9 — Figure EVs Source Data [file 44319_2024_140_MOESM9_ESM.zip › SD EV figures/Supplementary Figure 4 - EV4/Panel S4C/representative images/JPGs/C1-Control(G3)_i3Ns_01_26_21_Complete_MAP2_ATF4_512_1_4-MaxIP-1.jpg]

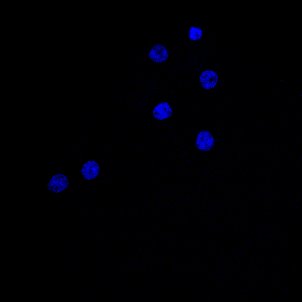

Supplement: Supplementary file 9 — Figure EVs Source Data [file 44319_2024_140_MOESM9_ESM.zip › SD EV figures/Supplementary Figure 4 - EV4/Panel S4C/representative images/JPGs/C1-Control(G3)_i3Ns_03_26_21_No_Glc_MAP2_ATF4_512_2_5-MaxIP-1.jpg]
