# Supplementary material for: Glucose hypometabolism prompts RAN translation and exacerbates C9orf72-related ALS/FTD phenotypes
Source: EMBO Rep. 2024 Apr 29;25(5):21. doi: 10.1038/s44319-024-00140-7 (PMC11094177; doi:10.1038/s44319-024-00140-7)
Supplement: Supplementary file 11 — Expanded View Figures [file 44319_2024_140_MOESM11_ESM.pdf]

Expanded View Figures

Figure EV1. Analysis of brain metabolites of C9-BAC vs. WT mice.

(**A**) Schematic depicting the bacterial artificial chromosome (BAC) transgene used to drive expression of the entire human *C9orf72* gene with a $(GGGGCC)_{100\text{-}1{,}000}$ repeat expansion in intron 1 (C9-BAC). Adapted from O'Rourke et al (2015). (**B**) LC-MS measurement of relative glucose concentrations in the frontal cortex of C9-BAC versus WT animals. (**C**) Schematic depicting the tricarboxylic acid (TCA) cycle pathway with all metabolic intermediates. LC-MS measurement of relative α-ketoglutarate concentrations in the frontal cortex of C9-BAC animals versus WT animals. (**D**) LC-MS measurement of the relative concentrations of four amino acids (isoleucine, cysteine, lysine, and ornithine) in the frontal cortex of C9-BAC vs. WT animals. (**E**) LC-MS measurement of relative $NADP+$, NADH, and NADPH concentrations in the frontal cortex of C9-BAC vs WT. All individual metabolite data are shown as median-normalized and log-transformed values (abbreviated as "Normalized conc."). For box and whisker plots, box edges denote upper and lower quartiles, horizontal lines within each box denote median values, whiskers denote maximum and minimum values, and shaded circles denote individual values for each animal. Student's two-tailed t-test, *$p < 0.05$, **$p < 0.01$. Source data are available online for this figure.

© The Author(s)

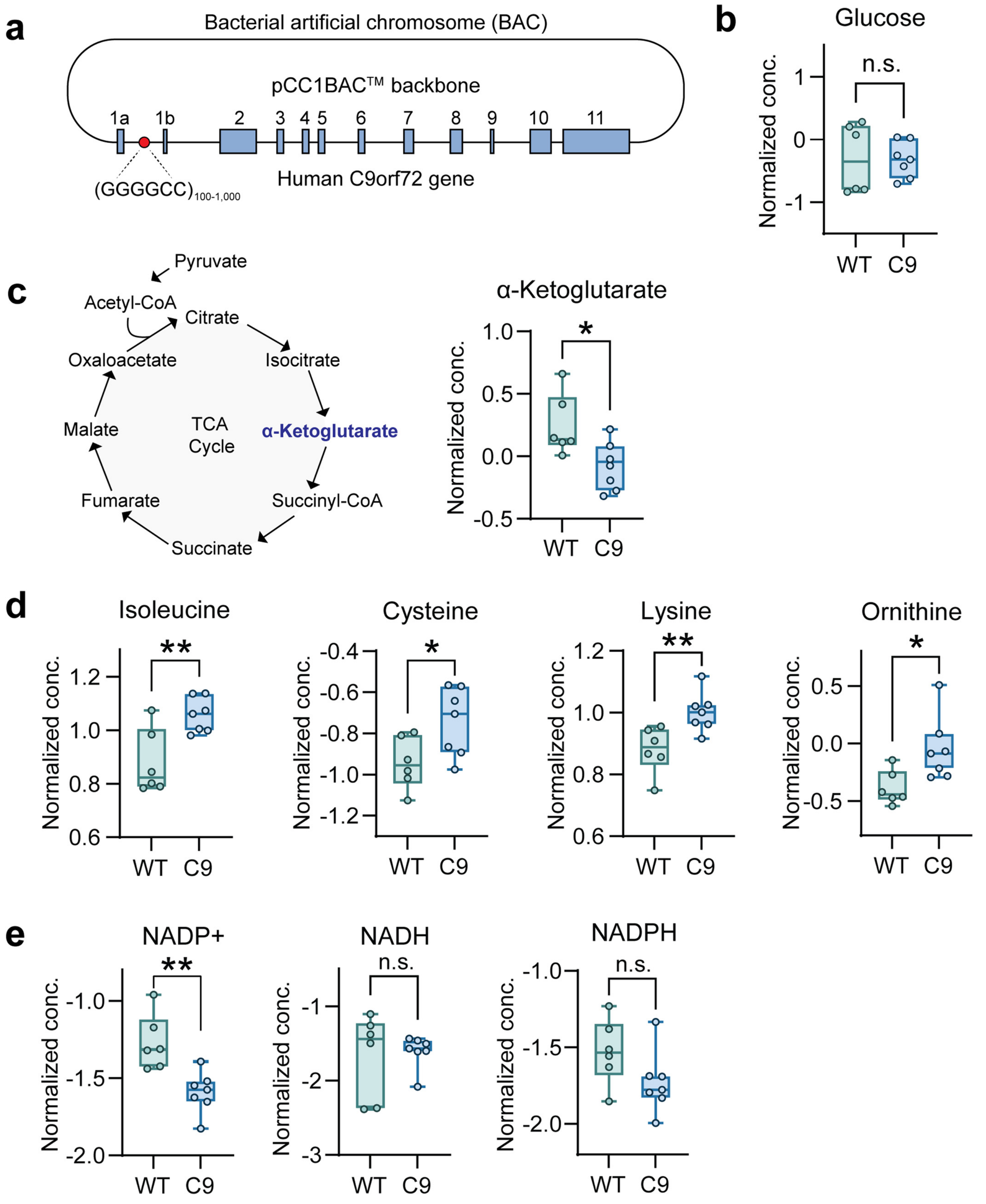
a
Bacterial artificial chromosome (BAC)
pCC1BAC™ backbone
1a 1b 2 3 4 5 6 7 8 9 10 11
(GGGGCC)100-1,000
Human C9orf72 gene
b
Glucose
n.s.
Normalized conc.
WT C9
c
Pyruvate
Acetyl-CoA
Citrate
Isocitrate
Oxaloacetate
Malate
TCA Cycle
α-Ketoglutarate
Fumarate
Succinyl-CoA
Succinate
α-Ketoglutarate
*
Normalized conc.
WT C9
d
Isoleucine
**
Cysteine
*
Lysine
**
Ornithine
*
Normalized conc.
WT C9
e
NADP+
**
NADH
n.s.
NADPH
n.s.
Normalized conc.
WT C9

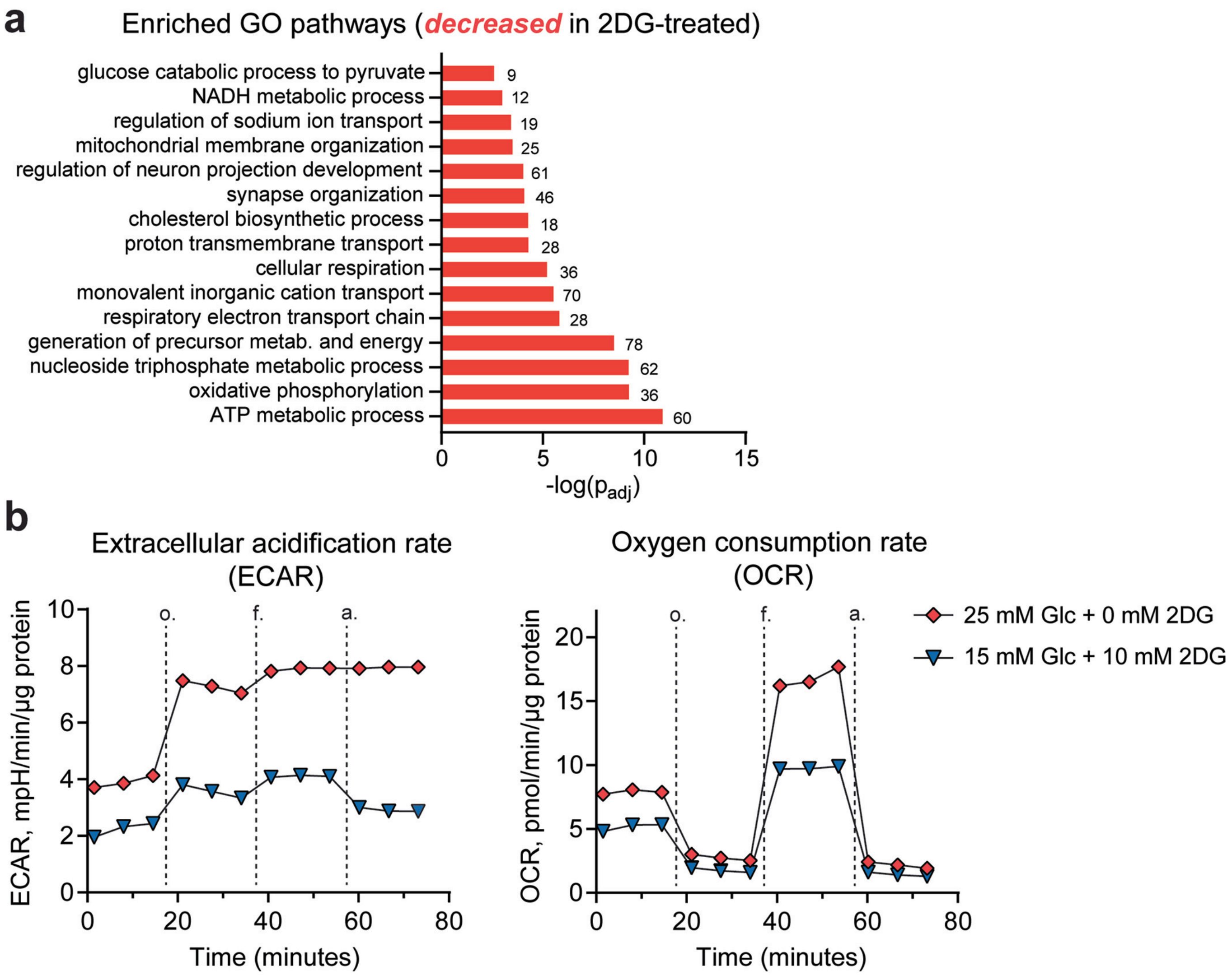

Figure EV2. Effect of 2DG on cellular respiration.

(**A**) RNA sequencing measurement of select downregulated gene ontology (GO) pathways in C9orf72 patient-derived i^{3}Neurons incubated with 10 mM 2DG-containing media versus normo-glucose media for 48 h ($n = 2$ individual i^{3}Neuron lines with 2 separate differentiations per line). (**B**) Seahorse extracellular flux assay-based measurement of extracellular acidification rate (ECAR) normalized to total protein and oxygen consumption rate (OCR) normalized to total protein in primary neurons incubated with either normo-glucose media or 10 mM 2DG-containing media for 48 h. Cells were sequentially treated with 1.5 μg/ml oligomycin (o.), 3 μM FCCP (f.), and 1 μM antimycin (a.) during the assay ($n = 1$ with 3 technical replicates). For (**A**), the values adjacent to each bar represent the number of altered genes in each GO pathway. The statistical test applied is the Fischer's exact test with a Benjamini–Hochberg False Discovery Rate (FDR). For (**B**) data are presented as mean ± SEM of assay technical replicates. Source data are available online for this figure.

 © The Author(s)

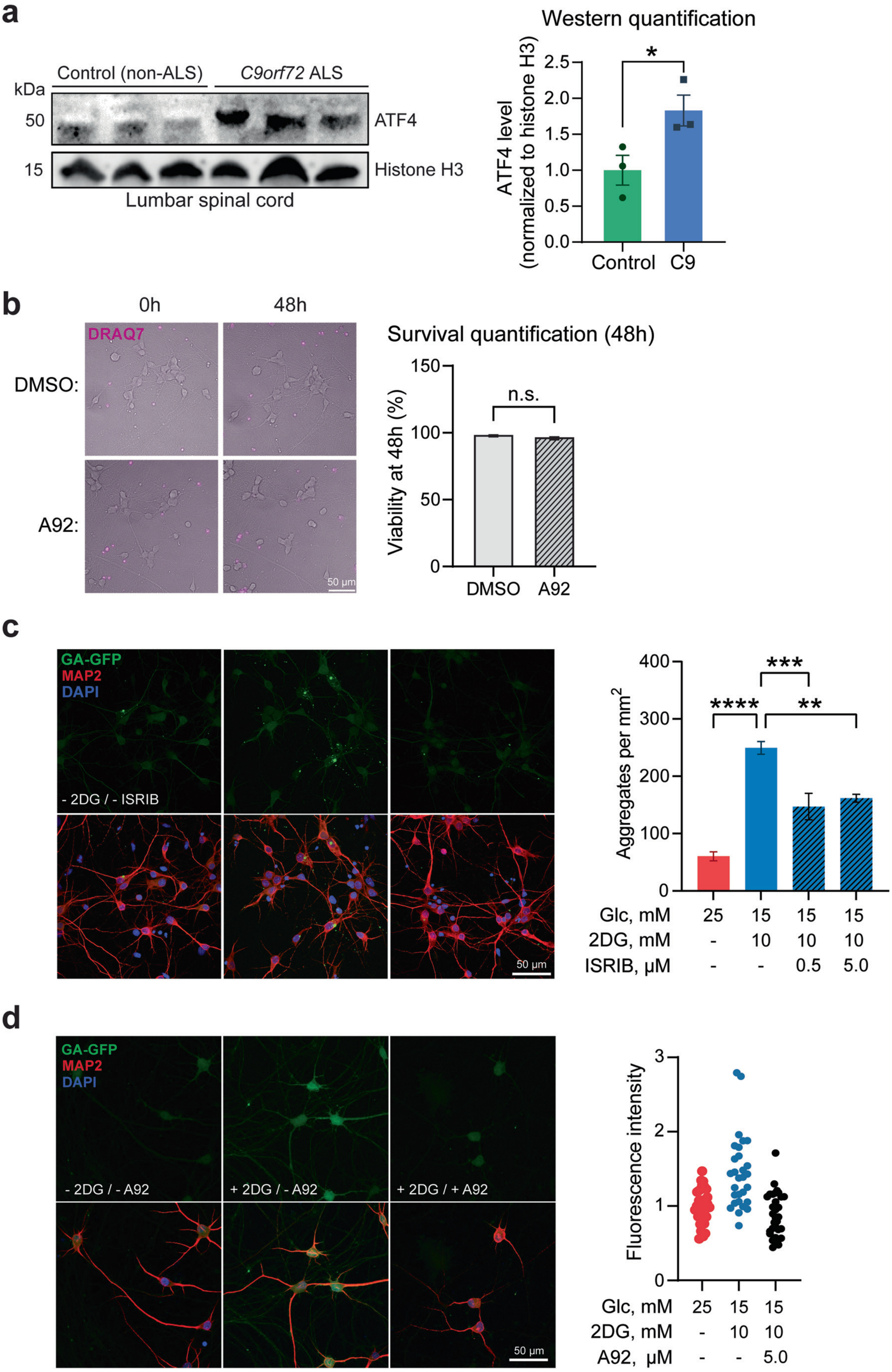
a
Control (non-ALS)
C9orf72 ALS
kDa
50
15
ATF4
Histone H3
Lumbar spinal cord
Western quantification
ATF4 level (normalized to histone H3)
2.5
2.0
1.5
1.0
0.5
0.0
*
Control
C9
b
0h
48h
DRAQ7
DMSO:
A92:
50 µm
Survival quantification (48h)
Viability at 48h (%)
150
100
50
0
n.s.
DMSO
A92
c
GA-GFP
MAP2
DAPI
- 2DG / - ISRIB
50 µm
Aggregates per mm2
400
300
200
100
0
***
****
**
Glc, mM 25 15 15 15
2DG, mM - 10 10 10
ISRIB, µM - - 0.5 5.0
d
GA-GFP
MAP2
DAPI
- 2DG / - A92
+ 2DG / - A92
+ 2DG / + A92
50 µm
Fluorescence intensity
3
2
1
0
Glc, mM 25 15 15
2DG, mM - 10 10
A92, µM - - 5.0

Figure EV3. Inhibition of ISR rescues DPR aggregation.

(**A**) Western blot analysis and quantification of ATF4 expression level relative to histone H3 expression level (as a loading control) in lumbar spinal cord tissue homogenates from either healthy control subjects or C9orf72-ALS patients ($n = 3$ subjects per genotype). (**B**) Live-cell imaging of human i^{3}Neurons treated with either 0.1% DMSO or 5.0 μM A92 and 0.1% DMSO for 48 h, with corresponding quantification of survival proportions. DRAQ7 was used as a fluorescent dead cell indicator ($n = 2$ i^{3}Neuron lines with 2 independent differentiations per line). Neurons were imaged live across 5 randomly selected fields of view/conditions. The same field of view was imaged daily. More than 100 neurons were tracked/condition/experiment. (**C**) Fluorescent confocal imaging and quantification of DPR aggregate formation in primary neurons transduced with RAN translation vector, then incubated with either normo-glucose media, 10 mM 2DG-containing media, or 10 mM 2DG-containing media with 0.5 μM or 5 μM ISRIB (all in the presence of 0.1% DMSO; $n = 4$). (**D**) Fluorescent confocal imaging and quantification of DPR formation in human i^{3}Neurons transduced with RAN translation vector, then incubated with either normo-glucose media, 10 mM 2DG-containing media, or 10 mM 2DG-containing media with 2.5 μM A92 (all in the presence of 0.1% DMSO; $n = 1$). At least 30 neurons were analyzed/condition. For (**A**, **B**), student's two-tailed t-test. For (**C**), one-way ANOVA with Dunnett's test for multiple comparisons. All data are presented as mean ± SEM. *$p < 0.05$, **$p < 0.01$, ***$p < 0.001$, ****$p < 0.0001$. Source data are available online for this figure.

 © The Author(s)

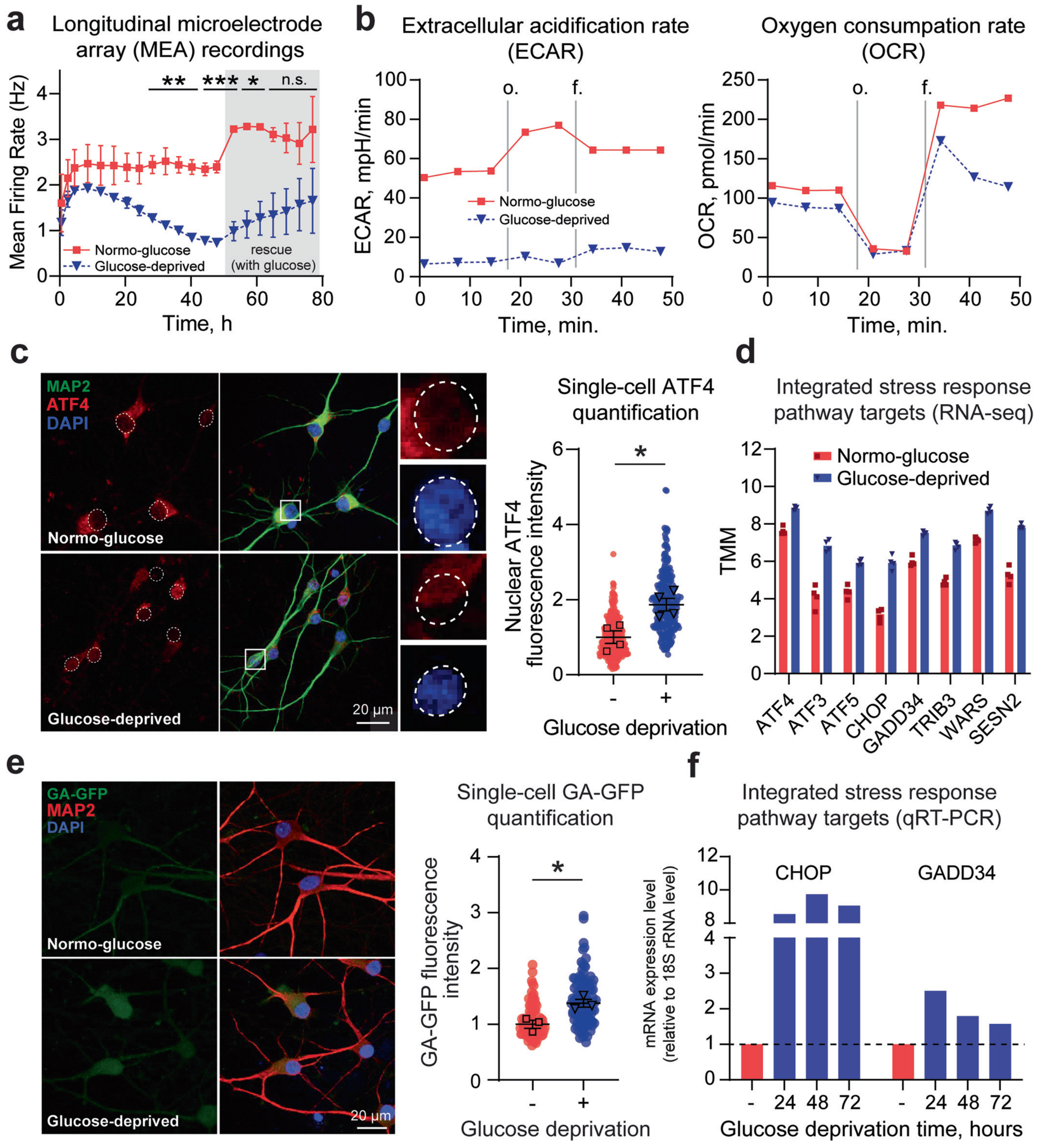
a
Longitudinal microelectrode array (MEA) recordings
Mean Firing Rate (Hz)
Time, h
Normo-glucose
Glucose-deprived
rescue (with glucose)
** *** * n.s.
b
Extracellular acidification rate (ECAR)
ECAR, mpH/min
Oxygen consumption rate (OCR)
OCR, pmol/min
Time, min.
o.
f.
c
MAP2
ATF4
DAPI
Normo-glucose
Glucose-deprived
20 µm
Single-cell ATF4 quantification
Nuclear ATF4 fluorescence intensity
Glucose deprivation
d
Integrated stress response pathway targets (RNA-seq)
TMM
ATF4
ATF3
ATF5
CHOP
GADD34
TRIB3
WARS
SESN2
e
GA-GFP
MAP2
DAPI
Single-cell GA-GFP quantification
GA-GFP fluorescence intensity
f
Integrated stress response pathway targets (qRT-PCR)
mRNA expression level (relative to 18S rRNA level)
CHOP
GADD34
Glucose deprivation time, hours

Figure EV4. Effect of glucose deprivation in I³neurons.

(**A**) Longitudinal microelectrode array (MEA)-based measurement of spontaneous neuronal activity of i³Neurons (healthy control line) incubated in either normo-glucose or glucose-deprived media over a 48-h period ($n = 3$ biological replicates). Glucose deprivation was initiated at time = 0 h. (**B**) Seahorse extracellular flux assay-based measurement of extracellular acidification rate (ECAR) normalized to total protein and oxygen consumption rate (OCR) normalized to total protein of primary neurons immediately following incubation with either normo-glucose media or glucose-deprived media for 48 h ($n = 1$ with 3 technical replicates). Cells were sequentially treated 1.5 µg/ml oligomycin (o.) and 3 µM FCCP (f.) during the assay. (**C**) Immunofluorescence-based measurement and quantification of nuclear ATF4 expression level in MAP2-positive primary neurons immediately following incubation with either normo-glucose media or glucose-deprived media for 48 h ($n = 4$ biological replicates). Neurons were imaged live across >5 randomly selected fields of view/conditions. The same field of view was imaged daily. More than 100 neurons were tracked/condition/experiment. (**D**) RNA sequencing analysis of select individual ISR target transcripts (all $p_{adj} < 0.05$) in i³Neurons immediately following exposure to either normo-glucose or glucose-deprived media for 48 h ($n = 2$ i³Neuron lines with 2 differentiations per line). (**E**) Representative images of i³Neurons transduced with RAN translation vector and then cultured in either normo-glucose or glucose-deprived media for 48 h, with corresponding quantification of GA-GFP fluorescence intensity ($n = 3$ biological replicates). Neurons were imaged live across 5 randomly selected fields of view/conditions. The same field of view was imaged daily. More than 100 neurons were tracked/condition/experiment. (**F**) qRT-PCR measurement of mRNA levels of two ISR transcriptional targets (CHOP and GADD34) in i³Neurons immediately following incubation with either normo-glucose or glucose-deprived media for either 24, 48, or 72 h ($n = 1$). For (**A**), multiple student's two-tailed t-tests. For (**C–E**), student's two-tailed t-test. All data (except **D** and **F**) are presented as mean ± SEM. *$p < 0.05$, **$p < 0.01$, ***$p < 0.001$. Source data are available online for this figure.

© The Author(s)

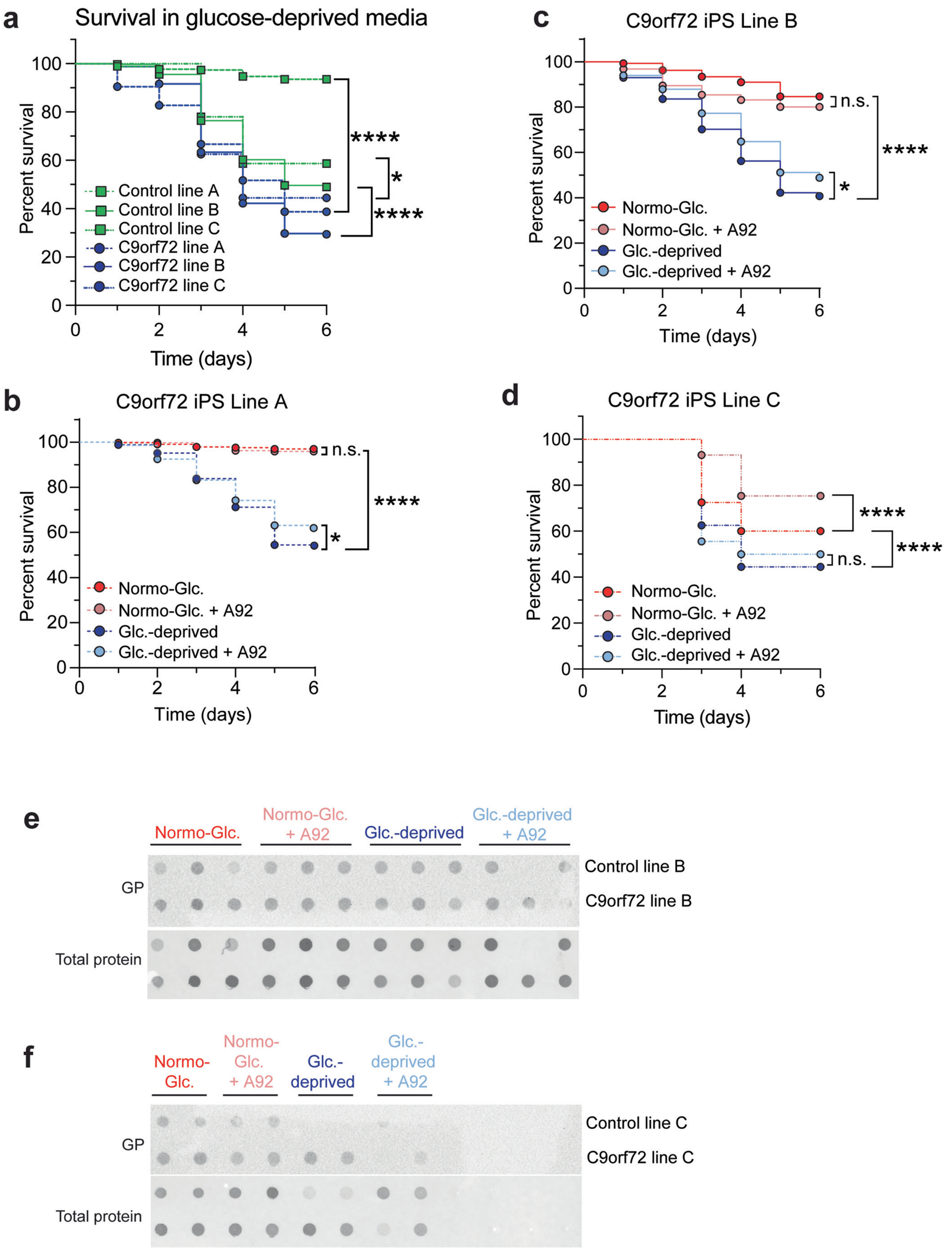
a
Survival in glucose-deprived media
Percent survival
Time (days)
Control line A
Control line B
Control line C
C9orf72 line A
C9orf72 line B
C9orf72 line C
****
*
****
b
C9orf72 iPS Line A
Percent survival
Time (days)
n.s.
****
*
Normo-Glc.
Normo-Glc. + A92
Glc.-deprived
Glc.-deprived + A92
c
C9orf72 iPS Line B
Percent survival
Time (days)
n.s.
****
*
Normo-Glc.
Normo-Glc. + A92
Glc.-deprived
Glc.-deprived + A92
d
C9orf72 iPS Line C
Percent survival
Time (days)
****
n.s.
****
Normo-Glc.
Normo-Glc. + A92
Glc.-deprived
Glc.-deprived + A92
e
Normo-Glc.
Normo-Glc. + A92
Glc.-deprived
Glc.-deprived + A92
GP
Total protein
Control line B
C9orf72 line B
f
Normo-Glc.
Normo-Glc. + A92
Glc.-deprived
Glc.-deprived + A92
GP
Total protein
Control line C
C9orf72 line C

Figure EV5. Glucose deprivation in control and C9orf72 derived I^{3}neurons.

(**A**) Kaplan–Meier survival analysis of i^{3}Neurons derived from either C9orf72 patients or healthy controls and maintained in glucose-deprived media ($n = 3$ i^{3}Neuron lines per genotype with 3 independent differentiations per line). Line A, B, C. ***p<. (**B**) Kaplan–Meier survival analysis of C9orf72 patient-derived i^{3}Neurons maintained in either normo-glucose or glucose-deprived media and treated with either 2.5 μM A92 with 0.1% DMSO or 0.1% DMSO only as vehicle control (3 independent differentiations per line). Line A. (**C**) Kaplan–Meier survival analysis of C9orf72 patient-derived i^{3}Neurons maintained in either normo-glucose or glucose-deprived media and treated with either 2.5 μM A92 with 0.1% DMSO or 0.1% DMSO only as vehicle control (3 independent differentiations per line). Line B. (**D**) Kaplan–Meier survival analysis of C9orf72 patient-derived i^{3}Neurons maintained in either normo-glucose or glucose-deprived media and treated with either 2.5 μM A92 with 0.1% DMSO or 0.1% DMSO only as vehicle control (3 independent differentiations per line). Line C. (**E**, **F**). Dot blot assessment of 8 M urea-soluble GP levels and relative total protein levels in i^{3}Neurons derived from either C9orf72 patients or healthy controls maintained in either normo-glucose or glucose-deprived media and treated with either 2.5 μM A92 with 0.1% DMSO or 0.1% DMSO only as vehicle control. (n = two independent lines per genotype with 2 or 3 technical replicates per treatment) Kaplan–Meier log-rank survival test. *$p < 0.05$, **$p < 0.01$, ****$p < 0.0001$. Neurons were imaged live across >5 randomly selected fields of view/conditions. The same field of view was imaged daily. More than 100 neurons were tracked/condition/experiment. All data are presented as mean ± SEM ($n = 3$ biological replicates). *$p < 0.05$, **$p < 0.01$. Source data are available online for this figure.

 © The Author(s)

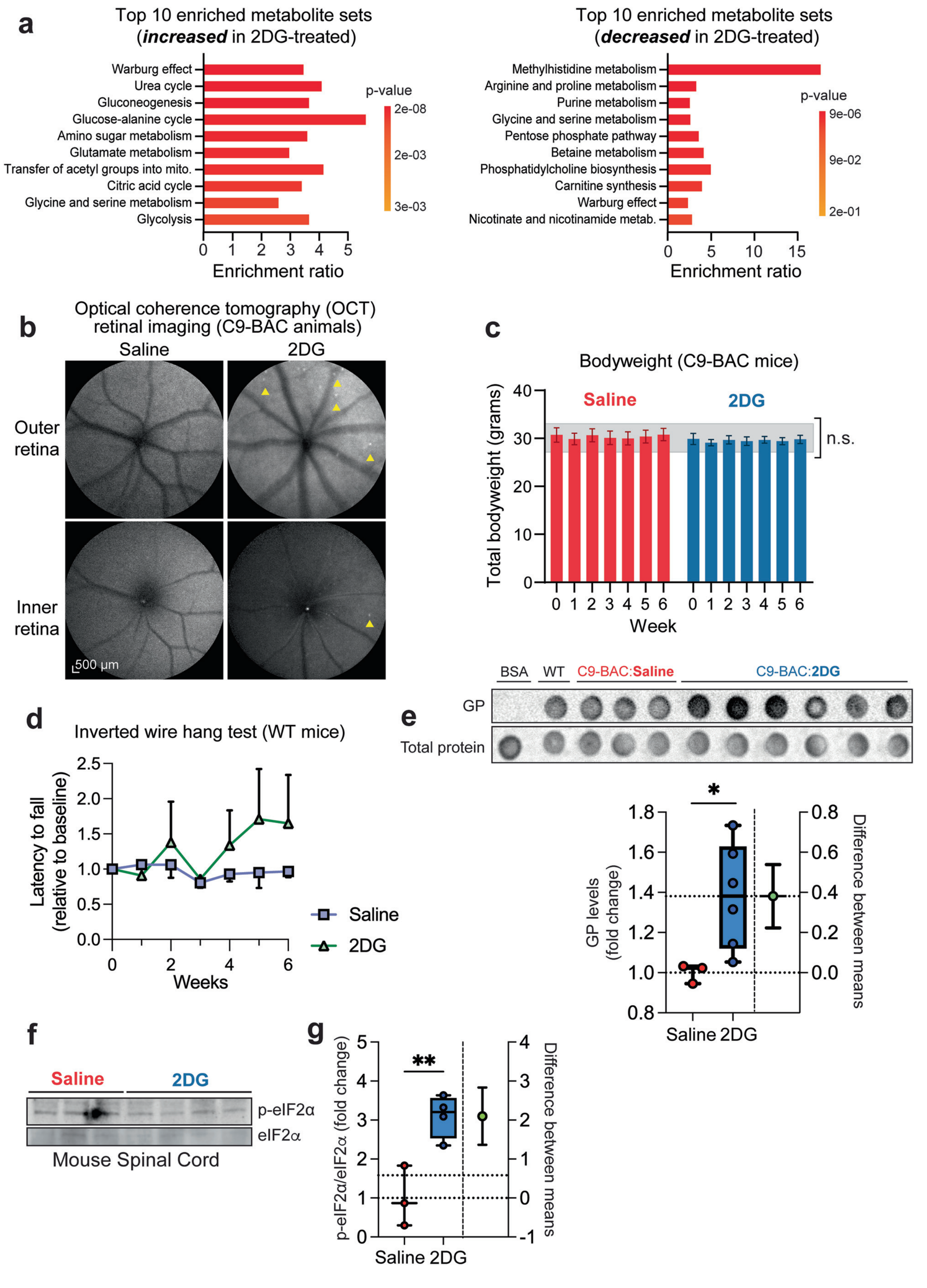
a
Top 10 enriched metabolite sets (increased in 2DG-treated)
Warburg effect
Urea cycle
Gluconeogenesis
Glucose-alanine cycle
Amino sugar metabolism
Glutamate metabolism
Transfer of acetyl groups into mito.
Citric acid cycle
Glycine and serine metabolism
Glycolysis
Enrichment ratio
p-value
2e-08
2e-03
3e-03
Top 10 enriched metabolite sets (decreased in 2DG-treated)
Methylhistidine metabolism
Arginine and proline metabolism
Purine metabolism
Glycine and serine metabolism
Pentose phosphate pathway
Betaine metabolism
Phosphatidylcholine biosynthesis
Carnitine synthesis
Warburg effect
Nicotinate and nicotinamide metab.
Enrichment ratio
p-value
9e-06
9e-02
2e-01
b
Optical coherence tomography (OCT) retinal imaging (C9-BAC animals)
Saline
2DG
Outer retina
Inner retina
500 µm
c
Bodyweight (C9-BAC mice)
Saline
2DG
n.s.
Total bodyweight (grams)
Week
d
Inverted wire hang test (WT mice)
Latency to fall (relative to baseline)
Weeks
Saline
2DG
e
BSA
WT
C9-BAC:Saline
C9-BAC:2DG
GP
Total protein
GP levels (fold change)
Difference between means
Saline 2DG
f
Saline
2DG
p-eIF2α
eIF2α
Mouse Spinal Cord
g
p-eIF2α/eIF2α (fold change)
Difference between means
Saline 2DG

Figure EV6. Effect of 2DG treatment in rodents.

(**A**) Enrichment analysis of significantly altered metabolite sets in the frontal cortex of C9-BAC animals immediately following chronic exposure to either 4 g/kg/week 2DG or saline ($n = 7$ animals per condition). Enrichment ratio represents the number of metabolites within each metabolite set that are either increased (on the left) or decreased (on the right). (**B**) Optical coherence tomography (OCT) retinal imaging of C9-BAC animals immediately following chronic exposure to either 4 g/kg/week 2DG or saline. Yellow arrows indicate hyperreflective foci ($n = 2$–3 animals per condition). (**C**) Longitudinal measurement of body weight during chronic exposure of C9-BAC animals to 4 g/kg/week 2DG or saline ($n = 7$ animals per condition). (**D**). Longitudinal assessment of inverted wire hang performance of wild-type littermate control animals chronically exposed to 4 g/kg/week 2DG ($n = 2$ animals) or saline ($n = 3$ animals). (**E**) Dot blot assessment and corresponding quantification of 8 M urea-soluble GP levels relative to total protein levels in the spinal cord C9-BAC animals treated with either saline ($n = 3$ animals) or 2DG ($n = 6$ animals). Bovine serum albumin (BSA) and spinal cord lysate from a wild-type animal were used as controls. (**F**) Western Blot analysis for p-eIF2α and eIF2α of mouse brain cortices treated with saline or 2DG. (**G**) Quantification of the ratio between pEif2α and Eif2α protein of the WB in Fig EV6f ($n = 3$ biological replicates). For (**A**), the statistical test applied is the Fischer's exact test with a Benjamini–Hochberg False Discovery Rate (FDR). For (**C**, **D**), two-way ANOVA. For (**E**–**G**), two-tailed t-test with Welch's correction. All data (except **A**) are presented as mean ± SEM. *$p < 0.05$, n.s. $p > 0.05$. For box and whisker plots, box edges denote upper and lower quartiles, horizontal lines within each box denote median values, whiskers denote maximum and minimum values, and shaded circles denote individual values for each replicate. Source data are available online for this figure.

 © The Author(s)

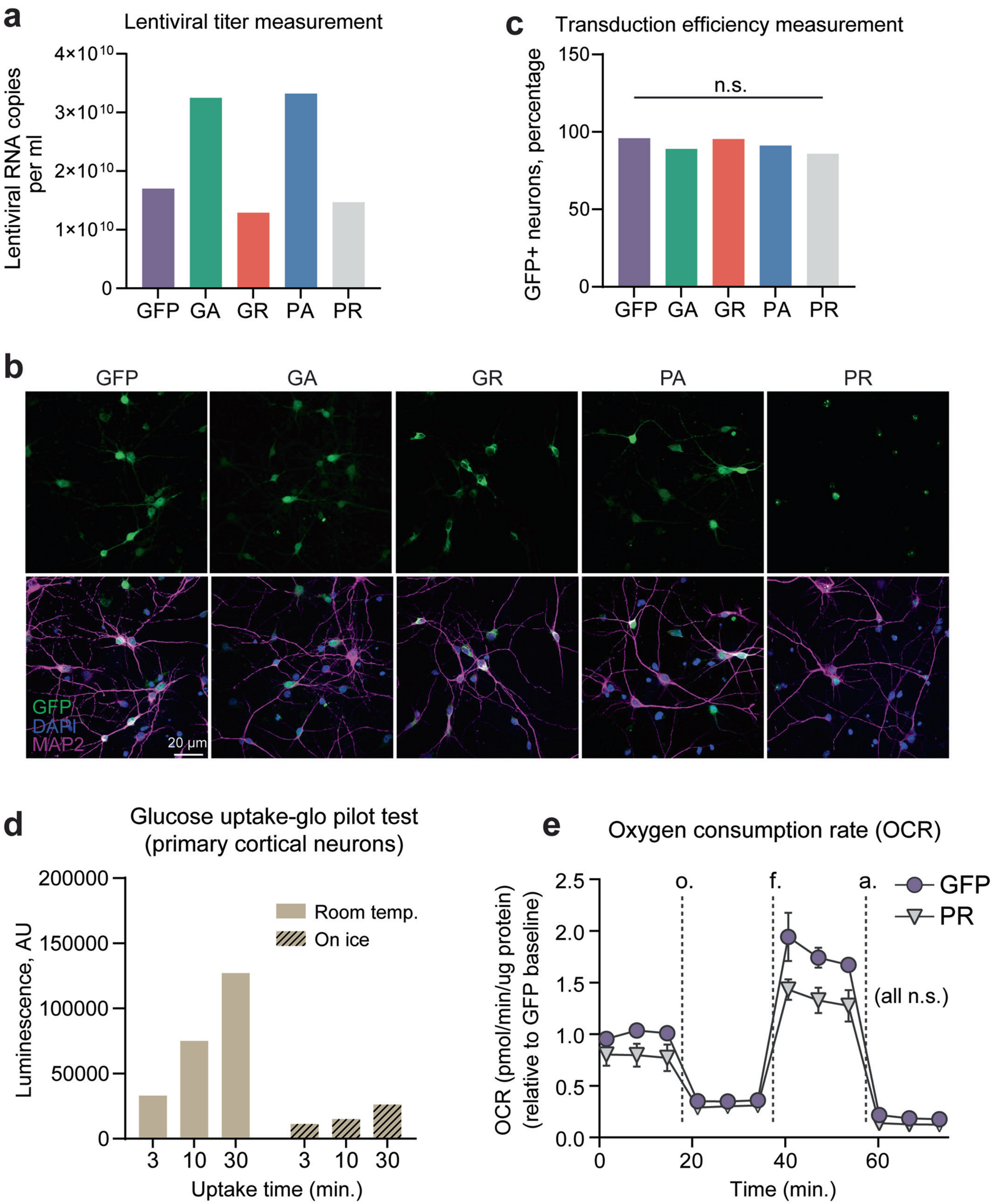
a
Lentiviral titer measurement
Lentiviral RNA copies per ml
4×10^10
3×10^10
2×10^10
1×10^10
0
GFP GA GR PA PR
c
Transduction efficiency measurement
GFP+ neurons, percentage
150
100
50
0
n.s.
GFP GA GR PA PR
b
GFP GA GR PA PR
GFP
DAPI
MAP2
20 μm
d
Glucose uptake-glo pilot test (primary cortical neurons)
Luminescence, AU
200000
150000
100000
50000
0
Room temp.
On ice
3 10 30 3 10 30
Uptake time (min.)
e
Oxygen consumption rate (OCR)
OCR (pmol/min/ug protein) (relative to GFP baseline)
2.5
2.0
1.5
1.0
0.5
0.0
o.
f.
a.
GFP
PR
(all n.s.)
0 20 40 60
Time (min.)

Figure EV7. DPRs transduction in rat cortical neurons.

(**A**) qRT-PCR measurement of DPR and GFP-only vector lentiviral titers ($n = 1$ technical replicate). (**B**) Representative images of primary neurons transduced with DPR or GFP-only lentiviral vectors, then stained for MAP2. (**C**) Quantification of the percentage of MAP2-positive cells also positive for GFP from (**B**) ($n = 1$ with 3 technical replicates). (**D**) Validation of luminescent assay (time- and temperature dependence) for measurement of glucose uptake in primary neurons ($n = 1$ with 3 technical replicates). (**E**) Seahorse extracellular flux assay measurement of oxygen consumption rate (OCR normalized to total protein) of primary neurons transduced with either the PR or GFP-only vector ($n = 3$ biological replicates). Cells were sequentially treated with 1.5 μg/ml oligomycin (o.), 3 μM FCCP (f.), and 1 μM antimycin (a.) during the assay. All data (except **A**) are presented as mean ± SEM. For (**C**), one-way ANOVA with Dunnett's multiple comparisons tests. For (**E**), multiple student's two-tailed t-test. n.s. $p > 0.05$. Source data are available online for this figure.

© The Author(s)
